# Multimodal prognostic features of seizure freedom in epilepsy surgery

**DOI:** 10.1136/jnnp-2021-327119

**Published:** 2022-03-04

**Authors:** Ali Alim-Marvasti, Vejay Niranjan Vakharia, John Sidney Duncan

**Affiliations:** 1 Department of Clinical and Experimental Epilepsy, Queen Square Institute of Neurology, University College London Faculty of Brain Sciences, London, UK; 2 Wellcome/EPSRC Centre for Interventional and Surgical Sciences, Department of Medical Physics and Biomedical Engineering, University College London, London, UK

**Keywords:** epilepsy, surgery, meta-analysis, neurosurgery

## Abstract

**Objective:**

Accurate preoperative predictions of seizure freedom following surgery for focal drug resistant epilepsy remain elusive. Our objective was to systematically evaluate all meta-analyses of epilepsy surgery with seizure freedom as the primary outcome, to identify clinical features that are consistently prognostic and should be included in the future models.

**Methods:**

We searched PubMed and Cochrane using free-text and Medical Subject Heading (MeSH) terms according to Preferred Reporting Items for Systematic Reviews and Meta-Analyses. This study was registered on PROSPERO. We classified features as prognostic, non-prognostic and uncertain and into seven subcategories: ‘clinical’, ‘imaging’, ‘neurophysiology’, ‘multimodal concordance’, ‘genetic’, ‘surgical technique’ and ‘pathology’. We propose a structural causal model based on these features.

**Results:**

We found 46 features from 38 meta-analyses over 22 years. The following were consistently prognostic across meta-analyses: febrile convulsions, hippocampal sclerosis, focal abnormal MRI, Single-Photon Emission Computed Tomography (SPECT) coregistered to MRI, focal ictal/interictal EEG, EEG-MRI concordance, temporal lobe resections, complete excision, histopathological lesions, tumours and focal cortical dysplasia type IIb. Severe learning disability was predictive of poor prognosis. Others, including sex and side of resection, were non-prognostic. There were limited meta-analyses investigating genetic contributions, structural connectivity or multimodal concordance and few adjusted for known confounders or performed corrections for multiple comparisons.

**Significance:**

Seizure-free outcomes have not improved over decades of epilepsy surgery and despite a multitude of models, none prognosticate accurately. Our list of multimodal population-invariant prognostic features and proposed structural causal model may serve as an objective foundation for statistical adjustments of plausible confounders for use in high-dimensional models.

**PROSPERO registration number:**

CRD42021185232.

**Key messages**

**What is already known on this topic**

- Surgery can be curative for some individuals with focal drug-resistant epilepsy but not others. Although various clinical prognostic features - such as unifocal temporal lobe lesions carrying a favourbale prognosis - are well-known, there are discrepancies in the scientific literature with regards to whether other features have prognostic value or not. Additionally, we have no accurate method to prognosticate. Therefore, this study reviewed meta-analyses that evaluated prognostic features of postsurgical seizure freedom.

**What this study adds**

- This study defines a list of ‘Essential Prognostic Features’ that were consistently prognostic across 38 evaluated meta-analyses of epilepsy surgery that had seizure-freedom as the primary outcome. We outline a structural causal model for statistical adjustments of plausible confounders and use in high-dimensional models. We propose a five-step plan for personalised seizure-freedom predictions, including collaborative multi-variable modelling.

**How this study might affect research, practice or policy**

- Our list of essential prognostic features might be especially useful in machine learning models of big-data on postsurgical seizure freedom. The proposed structrual causal model could be used in future research to adjust for known confounders. Instead of more meta-analyses, an international collaboration pursuing our proposed five-step plan may impel us towards attaining accurate personalised prognostication for epilepsy surgery.

## Introduction

Epilepsy surgery can be curative for focal drug-resistant epilepsy, yet in over half of individuals, seizures eventually relapse.^1 2^ Postsurgical outcomes include seizure freedom, discontinuation of antiseizure medications, neuropsychological and psychiatric outcomes or morbidity. Seizure freedom is the strongest predictor of improved health-related quality of life^3^ and is classified according to the ILAE or Engel systems.^4^ These outcomes can be used as ordinal scales, binarised into seizure-free and not seizure-free categories at specified postoperative time points or binarised at each year following surgery to build proportional Hazards models.^1 2^

Prognostic features can be related to patient characteristics (eg, age, seizure semiology, variability of seizures and genetics), investigation findings (focal lesion on MRI and localising epileptic activity on EEG), surgical factors (resection margins or technique) and combinations of the above (concordance of imaging with neurophysiology). Favourable clinically relevant prognostic features have been identified from many individual studies, including clearly localising and lateralising semiology, well-circumscribed unilateral, unifocal and temporal lesions, EEG-MRI concordance and complete excision of the evaluated epileptogenic zone.^5 6^

Other features are prognostic in some studies but not in others such as focal to bilateral tonic-clonic seizures (FBTCS)^5–10^ and age at seizure onset.^7–9 11^ A feature may erroneously appear prognostic in a single-centre study due to publication bias or overfitting from investigating many unadjusted variables. Conversely, a feature may appear falsely non-prognostic in small studies due to low statistical power. Most individual studies are small retrospective observational studies from single centres and are prone to such biases.

Meta-analyses aggregate data while accounting for different levels of heterogeneity among patients and between studies. Their strength lies in combining data to achieve greater statistical power while adjusting for heterogeneity and confounders, and attributing weights to studies resulting in summary effect size estimates with wider CIs than unweighted methods.

Nevertheless, accurately predicting seizure freedom prior to surgery has remained elusive. Machine learning models show promise, but have almost entirely been trained on temporal lobe (TL) surgeries.^12^ Other recent developments, such as the Epilepsy Surgery Nomogram and the modified Seizure Freedom score,^10^ are not better than clinical heuristics^13^ which have not resulted in improved surgical outcomes over recent decades.^14 15^ This highlights the need for a review of the evidence in epilepsy surgery, which we present here by evaluating meta-analyses for prognostic features of postsurgical seizure freedom. In the search for clinical features with robust prognostic value, we consider meta-analyses, because they are considered the pinnacle of evidence-based data.

Our objectives are to address these questions:

1. Which features are consistently prognostic, and could be used in models of seizure freedom?
  This list should also preclude the need for further meta-analyses on these features,^16 17^ other than to adjust for potential confounders.
2. Which features do not have prognostic value and could be excluded from future machine-learning models and meta-analyses? This would risk the potential loss of only very weak prognostic variables in exchange for better generalisability.
3. What variables have not been evaluated in meta-analyses and how can we improve postsurgical prognostication?

Methods

### Search strategy and Criteria

The study was registered on international prospective register of systematic reviews. The search was conducted in accordance with Preferred Reporting Items for Systematic Reviews and Meta-Analyses (PRISMA) guidelines on PubMed, MEDLINE and Cochrane updated 1 December 2020, using a combination of free-text and Medical Subject Heading (MeSH) terms. We screened titles and abstracts for inclusion criteria and full texts for exclusion criteria for individual prognostic features. Full search strategy and exclusions are in online supplemental methods.

10.1136/jnnp-2021-327119.supp1 Supplementary data

#### Inclusion criteria

We included studies for full-text review that were meta-analyses of prognostic features for seizure freedom in epilepsy surgery. The neurosurgical resections had to have been performed for patients with drug-resistant focal epilepsy with curative intent.

#### Data collection

Two neurologists and a neurosurgeon independently screened articles for inclusion criteria, then one collected data and checked against exclusion criteria (AA-M) and the other two checked decisions. Disagreements were resolved through discussion.

The following data, where available, were extracted for each meta-analysis: investigated feature(s) (whether prognostic or not), specified population (resected lobe, adults, specified lesion), numbers of patients and individual studies for each feature or their upper bounds, definition and duration of seizure freedom, effect sizes and method used (univariate, multivariate logistic regression, fixed effect, random effects, network analysis, meta-regression or other). Qualitative evaluation of certainty of evidence was performed using Grading of Recommendations Assessment, Development and Evaluation (GRADE) guidelines (online supplemental references 11–17).^18^ Trial sequence analyses were assessed for bias using an additional checklist.^19^ Where possible, we used the current International League against Epilepsy seizure classification.^20^

### Data presentation

Features from the same investigation modality were grouped into seven categories (online supplemental table 2).

Features were further split into essential prognostic features (EPF), uncertain prognostic feature (UPF), and non-prognostic feature (NPF) based on consistency of value across meta-analyses such that if all effect sizes were in the same direction (eg, all favoured postsurgical seizure freedom), then this feature was classified as EPF; whereas, UPF included features that in some meta-analyses favoured seizure freedom, while in others showed no effect or worse outcomes. NPFs were non-significant in all meta-analyses.

### Statistical analysis

Effect sizes were inverted such that OR and relative risks over 1 indicate better outcomes favoured good outcome. If effect sizes or CIs were not quoted, these were estimated from the raw data (online supplemental methods). When quoting effect sizes across meta-analyses for the same feature, we used range of effect sizes (ROES) for both point estimates and 95% CIs (min, max).

## Results

### Overview, PRISMA flowchart and meta-analytical methods

**Figure 1:**
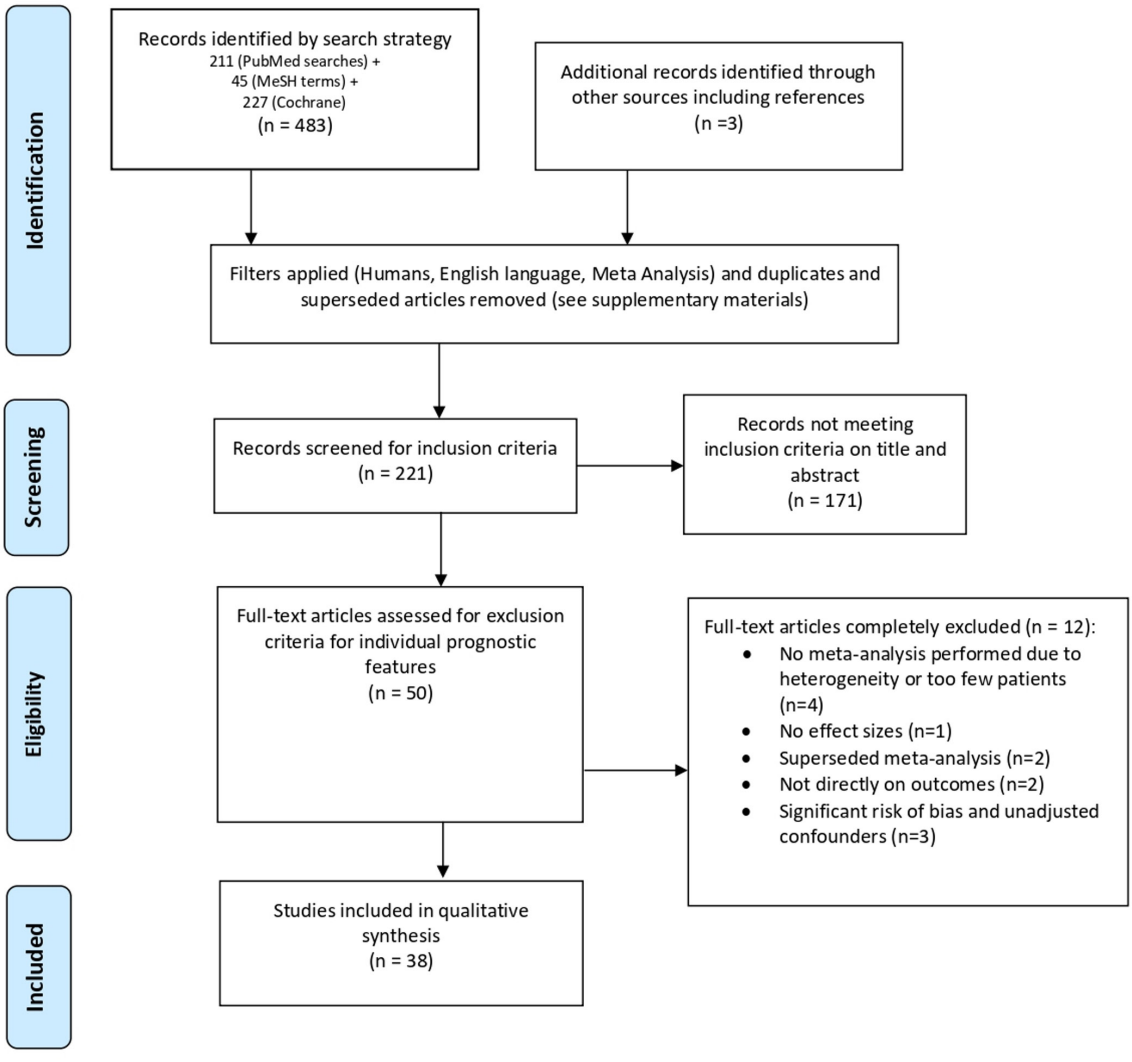
PRISMA flowchart of study selection. PRISMA, Preferred Reporting Items for Systematic Reviews and Meta-Analyses.

From 50 meta-analyses, 12 were excluded on full-text review, leaving 38 from which data were collected (PRISMA flowchart figure 1). Excluded meta-analyses had lower median numbers of individual studies than those from which data were extracted (11 (IQR 7–22) vs 22 (IQR 15–37)), and lower median number of patients (71 (IQR 33–87) vs 1034 (IQR 320–1999)). The largest number of individual studies in any meta-analysis was 258,^21^ and the highest number of included patients was 16 855 from the Cochrane review.^22^ Two multicentre studies were included, one from eight centres and another from 37.^23 24^

The main meta-analytical methods and upper bounds on numbers of studies and patients are summarised in table 1.

Online supplemental table 1 lists features from each meta-analysis with GRADE scoring, and online supplemental table 2 categorises these under seven modalities.

**Table 1:** The main meta-analytical methods for evaluating prognostic features of epilepsy surgery

Type of meta-analysis	Number of meta-analyses	Total number of included individual studies (upper bound)	Total number of patient participants (upper bound)
Univariate (tests of proportions, ANOVA, t-test or crude effect sizes)	9	215	6351
Proportional Hazards models (Cox regression)	1	19	187
Fixed or random (mixed) effects models	17	1122	55 502
Meta-regressions (including logistic regression)	6	372	16 006
(Bayesian) network analyses (NMA)	4	325	6471
Hierarchical/multi-level	0	0	0
Other: partial least squares (projection to latent space)	1	20	186

ANOVA, Analysis of Variance; NMA, Network Meta-Analysis.

Table 2 presents EPF that were consistently prognostic in all meta-analyses, and table 3 shows consistently NPF with individual GRADE scores. Online supplemental tables 3,4 provide more details on EPFs, features with UPFs and NPFs.

**Table 2:** Essential prognostic features for epilepsy surgery (EPF)

EPF		Prognostic value and supporting evidence base				
Feature	Population(s) or subgroup(s)	Range of effect sizes for seizure freedom	Comments	Meta-analytical references	Publication year (first, last)	GRADE score
Clinical features						
Severe developmental delay and IQ≤75	Children and adults, TLE, structural lesions, tuberous sclerosis, hemispherectomies	RR 0.14–0.66 (0.04, 0.94)	Favours absence of severe learning disability	Chelune, Naugle; Fallah, Guyatt; Hu, Zhang	1998–2019	++ Low
Febrile convulsions (FC)	TL and ET in children and adults	OR 2.08 (1.2, 3.7) RR 1.09(1.01, 1.17)	Favours presence of FC	Tonini, Beghi; West, Nevitt	2004–2019	+ Very low
Without acute postoperative seizures (APOS)	Children and adults, TLE and ET	OR 4.2–5.7 (2.97, 9.8)	Favours absence of APOS within 30 days of surgery	Giridharan, Horn	2016	++ Low
Imaging features						
Hippocampal sclerosis (HS)	Adults and children with TLE	OR 2.13 (1.57, 2.86) RR 1.17(1.12, 1.23)	Favours presence of Mesial Temporal Sclerosis or HS	Tonini, Beghi; West, Nevitt	2004–2019	++ Low
Abnormal or lesional MRI	Adults and children with TLE and ET, FCD, frontal lobe, occipital lobe and posterior quadrant epilepsies, hemispherectomies	RR 1.28–1.64 (1.20, 2.08) OR 1.27–4.6 (1.14, 16.62)	Favours abnormal MRI, see online supplemental table 3) for comments on two borderline meta-analyses	Tonini, Beghi; Téllez-Zenteno, Ronquillo; Yin, Kang; West, Nevitt; Rowland, Englot; Englot, Wang; Englot, Rolston; Harward, Chen; Widjaja, Jain; Cao, Liu	2004–2020	++ Low
SPECT: subtraction SPECT co-registered to MRI (SISCOM)	TL and ET	OR 2.44–3.28 (1.34, 5.67)	Favours ictal and inter-ictal SPECT-SISCOM abnormalities	Chen and Guo	2016	++ Low
Neurophysiological features						
Focal Ictal or interictal or invasive EEG	Adults, children, repeat resections, MRI-negative TLE, tuberous sclerosis, ET	OR 1.55–3.89 (1.24, 9.08) Positive prognostic value on PLS also.	Favours focal EEG changes, for comments on notable exceptions from 2012 to 2013^15 35^ see online supplemental table 3	Krucoff, Chan; Wang, Zhang; Fallah, Guyatt; Ibrahim, Morgan; Englot, Breshears	2013–2017	+ Very Low
Multimodal concordance						
EEG-MRI concordance	TL and ET children and adults, tuberous sclerosis, hemispherectomies	RR 1.25 (1.15, 1.37) OR 2.17–4.9 (1.07–13.5) Prognostic value on PLS	Favours EEG and MRI concordance	Tonini, Beghi; West, Nevitt; Fallah, Guyatt; Ibrahim, Morgan; Hu, Zhang	2013–2019	+++ Moderate
Surgical technique or anatomic features						
Temporal lobe (vs ET) resections	Adults and children with FCD, repeat surgery, low grade gliomas	OR 1.35–2 (0.8, 3.45)	Favours surgery for TLE	Rowland, Englot; Chen, Chen; Krucoff, Chan; Widjaja, Jain; Shan, Fan; Lamberink, Otte	2012–2020	+ Very Low
Complete excision (vs subtotal resection)	Adults and children with FCD, FLE, repeat resections, TLE, low grade gliomas	OR 2.6–12.5 (1.3, 20) RR 1.11–1.99 (1.03, 2.84)	Favours complete excision	Rowland, Englot; Chen, Chen; Englot, Wang; Krucoff, Chan; West, Nevitt; Widjaja, Jain; Shan, Fan	2012–2020	+++ Moderate
Pathological features						
Presence of tumours	Children and adults, TLE and ET, gangliogliomas, DNET, neuroepithelial tumours	RR 1.23 (1.14, 1.32) OR 1.27–2.78 (1.12, 3.57)	Favours tumours over multiple other lesions. See comments in online supplemental table 3	Tonini, Beghi; West, Nevitt; Lamberink, Otte	2004–2020	+++ Moderate
Focal cortical dysplasia (FCD)	Adults and children, TLE and ET	FCD: RR 0.90 (0.85, 0.95) FCD type II(b): OR 1.38–1.92 (1.01, 3.57)	Favours the absence of FCD, otherwise favours FCD type IIb	Rowland, Englot; Chen, Chen; West, Nevitt; Lamberink, Otte	2012–2019	++ Low
Lesional pathology vs non-lesional	Adults and children, FLE, TLE, ET, repeat resections, occipital lobe and posterior quadrant.	RR 1.67 (1.36, 28.6) OR 1.08–3.2 (1.02, 5.3)	Favours presence of focal pathological lesion except in MRI neg TLE (see online supplemental table 3) comments)	Englot, Wang; Englot, Rolston; Krucoff, Chan; Wang, Zhang; Harward, Chen; Englot, Breshears; Widjaja, Jain	2012–2017	++ Low

The essential prognostic features (EPFs).

See online supplemental table 3 for more details and full list of references.

ET, extratemporal; FCD, focal cortical dysplasia.; FLE, frontal lobe epilepsy; OR/RR, OR and relative risks over 1 indicate better outcomes; PLS, projection to latent space; TL, temporal lobe; TLE, Temporal Lobe Epilepsy.

**Table 3:** Non-prognostic features (NPF)

NPF features		Non-prognostic evidence base					
Feature	Population(s) or Subgroup(s)	Comments	Individual patients*	Individual studies*	Meta-analytical references	Publication years (first, last)	GRADE score
Clinical features							
Sex: male vs female	Adults and children with FLE, TLE, ET, tuberous sclerosis, MRI neg TLE, repeat surgery, hemispherectomies, low grade gliomas	All were non-significant, a large proportion even on weighted univariate tests, which otherwise tend to overestimate significance. Individual unweighted effect sizes ranged from OR 0.83 (0.42, 1.64)^c^ in repeat surgery for focal DRE^14^ to OR 1.44 (0.86, 2.41) in MRI negative TLE.^25^	5974	148	Englot, Wang; Englot, Rolston; Zhang, Hu; Fallah, Guyatt; Ibrahim, Morgan; Wang, Zhang; Krucoff, Chan; Englot, Breshears; Hu, Zhang; Shan, Fan; Cao, Liu	2012–2018	+++ Moderate
Epilepsia partialis continua (EPC)	Children undergoing hemispherectomies	Not significant on unweighted univariate testing and result is from only one meta-analysis.	127	7	Cao, Liu	2016	++ Low
Imaging features							
Number of cortical tubers	Tuberous sclerosis	Numbers of tubers did not predict outcomes. See online supplemental table 5 for observations on methodology, sample sizes, adjustments and heterogeneity.	286	24	Zhang, Hu; Fallah, Guyatt; Ibrahim, Morgan	2013–2015	++ Low
Magnetic ^1^H spectroscopy	TLE adults and children	Probably no more valuable than conventional MRI abnormality (online supplemental table 5).	121	22	Willmann, Wennberg	2006	+ Very low
Encephalomalacia	Adults and children	Encephalomalacia was NS in the Cochrane meta-analysis, it was also not significant on subgroup analyses.^22^	317	5	West, Nevitt	2019	+ Very Low
Enhancement, oedema, and/or mass effect	Low grade gliomas in adults	These combined features are not clinically prognostic of low-grade glioma resection for seizure freedom. Although NS, the point estimate and CI are unavailable.	2641	23	Shan, Fan	2018	+ Very low
Vascular lesions	Adults and children with TL and ET	Only one meta-analysis investigated this in 2004, comprising only three individual studies, its pathological counterpart was also NS.^22^	<<3511	3	Tonini, Beghi	2004	+ Very low
Neurophysiological features							
Intraoperative invasive EEG	Children and adults with FLE	Electrico-corticography did not effect outcomes	1024	21	Englot, Wang	2012	+++ Moderate
Video telemetry and long-term monitoring	Children and adults with FLE, lesional and non-lesional TLE and ET	Lesional TLE cases do well, and this was the only subgroup in which long-term monitoring had a point effect size estimate greater than 1.	1738	65	Englot, Wang; Kobulashvili, Kuchukhidze	2012, 2018	+ Very low
Surgical technique or anatomic features							
Mesial vs lateral TL focus	MRI neg TLE	Mesial or lateral TLE, as determined by sEEG, subdural grids, or ATL/SAH vs neocortectomy, are not significant.	92	8	Wang, Zhang	2016	+ Very low
Side of resection (left vs right)	Children and adults, Non-lesional, TLE, FLE, ET, MRI negative TLE, repeat surgery, hemispherectomies	This feature is unlikely to be prognostic—see online supplemental table 5.	6550	188	Tonini, Beghi; West, Nevitt; Willmann, Wennberg; Ansari, Tubbs; Englot, Wang Englot; Rolston; Wang, Zhang; Krucoff, Chan; Englot, Breshears; Hu, Zhang; Ansari, Maher	2004–2019	+++ Moderate
Frontal, central, or posterior resections vs other	ET, adults, non-lesional	Not prognostic	81	?	Ansari, Tubbs	2010	+ Very low
Geographical location of surgery	Tuberous sclerosis in children	Only one meta-analysis investigated North America vs Elsewhere, and the GRADE score is from this meta-analysis alone.	186	20	Ibrahim, Morgan	2015	+++ Moderate
Pathological features							
Neuro-migrational defects	TL and ET children and adults	There was a trend whereby neuromigrational deficits were negative prognostic factors, but the number of participants in this analysis was unclear.	?	6	Tonini, Beghi	2004	+ Very Low
Astrocytoma vs non-astrocytoma	Low grade gliomas in adults	The exact numbers of patients were not provided for this particular analysis.	<2641	<23	Shan, Fan	2018	+ Very Low

See online supplemental table 5 for more details and online supplemental materials for full list of references.

*Upper bound of estimate, not including subgroup analyses.

^m^, multivariate; ^u^, univariate; ^c^, calculated (usually unweighted) effect size; ET, extratemporal; FLE, frontal lobe epilepsy; MCD, malformations of cortical development; NS, not significant; PLS, projection to latent space; TL, temporal lobe.

### EPF for epilepsy surgery

Thirteen features were regarded as EPF, as they were consistently prognostic. Three clinical features, from six meta-analyses over 21 years, were severe learning disability including IQ<75, with the largest effect size estimates from the paediatric tuberous sclerosis population (ROES RR 0.26–0.66 (0.14 to 0.94), OR 0.14–0.61 (0.04 to 0.82)), presence of febrile convulsions (RR 1.09 (1.01 to 1.17)) and lack of acute postoperative seizures (OR 4.2 (2.97 to 5.93)) (table 2 and online supplemental table 3).

Prognostic imaging features included the presence of hippocampal sclerosis (RR 1.17 (1.12 to 1.23)) and abnormal Single-Photon Emission Computed Tomography (SPECT) coregistered with MRI (ROES 2.44–3.28 (1.34 to 5.67)). Abnormal MRI was consistently prognostic in 10 meta-analyses with the largest effect sizes from children having hemispherectomies (ROES RR 1.28–1.64 (1.20 to 2.08), OR 1.27–4.6 (1.14 to 16.62)).

Neurophysiological features were ictal and interictal (uni-)focal EEG abnormalities, this effect largely persisted irrespective of whether the MRI was abnormal or if initial epilepsy surgery had failed (ROES OR 1.55–3.89 (1.24 to 9.08)).

Concordant MRI and EEG abnormalities were consistently associated with a good prognosis (ROES OR 2.17–4.9 (1.07 to 13.5)). There were no genetics features in EPF.

Surgical technique EPFs were TL resections (in populations that excluded repeat resections and surgery for low grade gliomas) (ROES OR 1.35–2 (1.06 to 3.45)) and complete excision of lesions (ROES RR 1.11–1.99 (1.03 to 2.84)).

Favourable histopathological features were: (1) presence of tumours (RR 1.23 (1.14 to 1.32)), (2) focal cortical dysplasia type IIb (FCD) (ROES OR 1.38–1.92 (1.01 to 3.57)), (3) presence of any focal pathological lesion (ROES OR 1.08–3.2 (1.02 to 5.3)). One meta-analysis, however, showed non-significance for focal histopathology in MRI-negative temporal lobe epilepsy (TLE),^25^ suggesting that the basis of favourable outcomes in individuals with focal imaging abnormalities is a histopathological abnormality (see structural causal model (SCM) in online supplemental materials).

Concordance and complete excision had moderate quality of evidence scores, other results were of low or very low quality.

### Uncertain prognostic features

Eighteen features had mixed results with some meta-analyses suggesting prognostic value and others suggesting non-significance: previous head injury, central nervous system (CNS) infections, focal semiology, infantile spasms, seizure frequency, age at onset, age at surgery (investigated by 18 separate meta-analyses), duration of epilepsy (15 meta-analyses), interictal fluorodeoxyglucose positron emission tomography (FDG-PET) focal hypometabolism, preoperative invasive EEG or choice of subdural versus depth electrodes, presence of interictal spikes, lateralising ictal or interictal EEG, extensive surgical resections and vascular pathology (online supplemental table 4).

### Non-prognostic features

Fifteen non-prognostic features (table 3 and online supplemental table 5) comprised: sex, epilepsia partialis continua, number of cortical tubers, magnetic spectroscopy abnormality, encephalomalacia, enhancement or mass effect of low grade gliomas, performing intraoperative invasive electrocorticography, use of video-EEG telemetry, mesial versus lateral temporal focus, side of resection, frontal-central or posterior extratemporal lobe resections, geographical location of surgery (North America vs elsewhere), presence of neuronal migration abnormalities on imaging and astrocytoma versus non-astrocytoma.

## Discussion

We identified 46 features from 38 meta-analyses on prognostication in epilepsy surgery, only 15 of which were in the 2019 Cochrane review.^22^ We categorised features that were consistently prognostic. When investigating other variables for associations with seizure outcomes, EPFs can be used to adjust for confounders.

### EPF for epilepsy surgery

**Figure 2:**
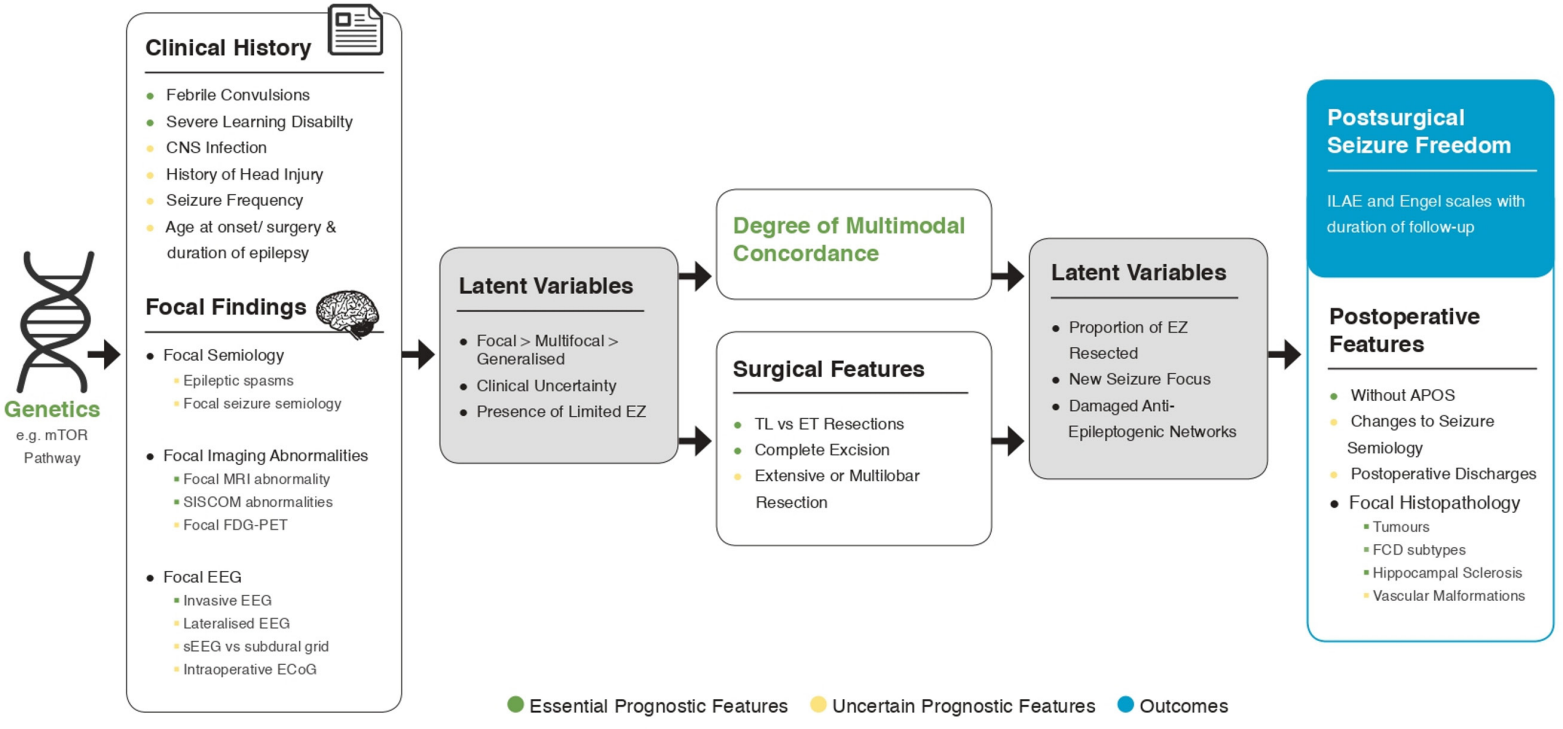
Outline of a structural causal model with latent variables for postsurgical seizure freedom. ET, extratemporal; FCD, focal cortical dysplasia; TL, temporal lobe; ILAE, international league against epilepsy; EZ, epileptogenic zone; FDG-PET, fluorodeoxyglucose positron emission tomography; EEG, electroencephalogram.

EPF is a minimum essential list based on current best-evidence. Our objective was to determine a minimum list of *a priori* features for use in future models, to improve personalised prognosis and outcomes (table 2). We further +propose grouping these features into an *a priori* SCM, to determine if it would be appropriate to adjust for these variables in future studies (see SCM in online supplemental materials, summarised in figure 2).^26^

A 2006 assessment of 3511 patients from 47 articles^27^ suggested that the following were associated with a higher chance of seizure remission: prolonged febrile seizures, unilateral EEG epileptiform abnormalities, MRI abnormalities, hippocampal sclerosis, SPECT ictal focal hyperperfusion, PET TL abnormalities and extent of mesial temporal resections. Head trauma, postoperative epileptiform EEG changes, developmental abnormalities with hippocampal sclerosis and acute postoperative seizures were negatively prognostic.^27^ Due to unadjusted confounders and heterogeneous definitions of features and seizure freedom, such findings were considered preliminary.^27 28^ Another meta-review of 10 reviews and meta-analyses identified lesional, abnormal MRI, focal seizures, complete resection, unifocal ictal EEG abnormality and extensive lobectomy versus tuberectomy, in patients with tuberous sclerosis, as positive predictors. Severe developmental delay, non-localised or bilateral EEG, FCD type 1, abnormal postoperative EEG and tuberectomies were negative predictors.^29^

#### Lesional and abnormal MRI

A meta-analysis with low study heterogeneity concluded an OR of 2.5 (2.1 to 3.0) in favour of lesional cases with an overall RR of 1.4 (p<0.001; 2860 lesional, 697 non-lesional, 40 studies).^30^ This trend was maintained for temporal and extratemporal subgroups. Similar results were found in other meta-analyses for occipital lobe epilepsy and in patients undergoing repeat surgery. When lesions were defined by pathology, MRI abnormalities still had a non-significant trend to higher rates of seizure freedom.^14^ Another meta-analysis found a prognostic trend for abnormal pathology even in MRI-negative TLE (p=0.06, OR=1.36 (0.7 to 2.63)).^25^ Within lesional-epilepsies, FCD type IIb is further associated with better outcomes.^31^

Although it is well established that lesional epilepsies have better postsurgical outcomes,^14 21 22 25 30 32 33^ and that complete lesionectomy is required^15 21 22 31 34 35^ the overwhelming majority of studies did not adjust for these.

A meta-analysis of 1999 patients across 35 articles, found outcomes after stereo-electroencephalogram (SEEG), were better than after subdural grids in patients undergoing temporal resections with lesional-MRI (seizure freedom for subdural grid (51.5% to 61.9%) vs SEEG (64.4% to 81.6%)).^36^ Such a comparison is limited by ascertainment bias and the differing indications for these methods. Interactions between features have not been formally investigated in meta-analyses, except in specific subpopulations and imaging-EEG concordance.

#### Multimodal concordance

Five meta-analyses attested the value of concordant MRI and EEG,^22 28 37–39^ but none looked at the value of semiological concordance with other modalities. In our SCM, the prominent causal pathway node is multimodal concordance, which should be further studied as a valuable predictor of seizure freedom (figure 2).

### Features of uncertain significance

Even meta-analyses may be underpowered, contributing to lack of statistical significance (online supplemental reference 64). PET results were mixed, but when concordant with EEG, PET could predict good seizure outcomes in non-lesional TLE with a positive predictive value of 71% (online supplemental reference 27).

Most meta-analyses reported non-significance of age at seizure onset, age at surgery and duration of epilepsy; however, there is a mixed picture. For every extra year of duration of epilepsy at time of surgery, one metaregression reported overall odds of seizure freedom reduced by a factor of 0.83 and another analysing data from 1545 patients across 12 studies found shorter duration of epilepsy was associated with higher rates of postsurgical seizure freedom with RR ranging from 1.20 to 1.33 (online supplemental reference 57). Conversely, age at surgery and duration of epilepsy before surgery have been documented as having ‘no association’ with outcomes.^27^

Longer duration of epilepsy may result in worse surgical outcomes due to selection bias (more difficult cases being deferred) or progressive cerebral damage. Strikingly, these three features of age at onset, age at surgery and duration of epilepsy have not been explored for three-way interactions.

Uncertain features can be reclassified into the essential or non-prognostic categories, when future studies that evaluate their value adjust for essential prognostic variables. While such models would clarify to what extent uncertain features may be prognostic over and above the essential features, this may not always be clinically desirable. For example, CNS infections may result in glial scars, and adjusting for imaging lesions may not be clinically desirable. Instead, an SCM could be used (see five-step plan below).

### Non-prognostic features

Side of resection and sex were both investigated in 11 meta-analyses but were not prognostic, consistent with a meta-review from 2013.^29^ Nevertheless, studies have continued to investigate them. Their use in predictive models risks overfitting and compromising generalisability.

### Prognostication: common pitfalls and recommendations

#### Unmodelled features

As there has not been significant improvement in postoperative outcomes, there are likely to be variables that have not been included.^14 15^ This is problematic for two reasons. First, studies are unable to adjust for unknown confounders. Second, without these features, individualised predictions will not be accurate. It is, therefore, critical to discuss notable missing features.

No meta-analysis has investigated the role of family history or detailed seizure semiology despite the fact that monitoring seizure semiology is integral to presurgical evaluation. Five meta-analyses reviewed MRI-EEG concordance, but none considered semiological concordance; the closest corollaries were FBTCS, epilepsia partialis continua and epileptic spasms. Future studies should evaluate interactions between semiology, epileptogenic zone, imaging and neurophysiology in patients with both favourable and unfavourable surgical outcomes.

The importance of genetics in seizure-free outcomes is belied by relatively few publications. Individuals with mutations affecting synaptic transmission or ion channels (5 articles, 14 patients) were less likely to benefit from epilepsy surgery than those with mutations in the mechanistic target of rapamycin (mTOR) pathway (10 articles, 30 patients). This was despite six of eight patients with SCN1A mutations having concordant semiology and colocalised MRI lesions.^40^ This meta-analysis was the only one to investigate genetics but it met our exclusion criteria as a large proportion of the small samples were lesional and no attempt at adjustments had been made (online supplemental tables).^40^ High-frequency oscillations and fast ripples were also excluded in our final synthesis (figure 1) due to lack of appropriate effect sizes (online supplemental references 1,6 and online supplemental table 1). This should impel us towards multicentre data sharing in comprehensive models (figure 2).

Other notable factors omitted from meta-analyses include analysis of cerebral structural connectivity (online supplemental reference 65) and resection of the piriform cortex as part of anterior TL resections (online supplemental reference 66).

#### Towards personalised seizure freedom predictions

Meta-analyses have been widely used for over five decades to quantitatively integrate a collection of studies. They are useful to identify important features based on best-available evidence, but cannot identify new features or provide personalised quantitative prognostication. The majority of studies did not statistically correct for multiple comparisons, potentially introducing false positives.

Machine learning models and nomograms have been proposed to predict outcomes, without prospective validation.^10 12^ These models included three features of uncertain significance (duration of epilepsy, frequency of seizures and generalised seizures), one non-prognostic factor (sex) and one EPF (pathological aetiology); it is perhaps unsurprising that the model was not generalisable.^13^ We advocate, therefore, that to improve prognostication and outcomes, a five-step plan is adopted:

1. All relevant factors for epilepsy surgery outcome prediction are curated in an agreed international, multicentre endeavour, which include the essential prognostic list curated here. Practically, the preoperative clinical variables should take precedence over postoperative features, for example, *severe developmental delay* should take priority over *acute postoperative seizures* and *FCD type IIb* as the latter two are only known after surgery.
  The final curated features would then form the starting point for building predictive models.
2. An SCM is devised that links outcomes to prognostic factors, to enable adjusting for EPFs when investigating other variables.
3. Identification of the degree to which polygenic risk scores, family history, seizure semiology and concordance may contribute to outcomes as indirect measures of seizure focality within the SCM.
4. Curation of an international multicentre, high-quality, anonymised retrospective and prospective data set of patients who have undergone epilepsy surgery with features and outcomes, similar to the retrospective collaboration on surgical histopathology.^24^
  A challenge in multicentre data collection will be to ensure that clinical and investigatory data are collected in a consistent and standardised manner, the details of which should be finalised in the protocols of the multicentre collaboration.
5. Machine learning models suitable for binary features and outcome classification on the international dataset.

The current study addressed the first two steps including R code to generate and amend SCMs (see online supplemental materials section on SCM for details on R codes for a simplified and complete SCM, and the two online supplemental files: SCM dagitty V.5 super simplified and SCM dagitty V.4). We can verify the value of EPFs and the SCM by building high-dimensional predictive models from international collaborations using SCM to adjust for covariates, subsequently showing that the resulting model predicts outcomes better than current methods.

10.1136/jnnp-2021-327119.supp2 Supplementary data

10.1136/jnnp-2021-327119.supp3 Supplementary data

### Limitations

Meta analyses were our unit of analysis, each assuming sufficient homogeneity for estimation of pooled effects.^18^ Only English-language articles were searched and we did not check for overlaps between meta-analyses, we, therefore, quote upper limits of numbers of patients and individual studies. We adopted the same definitions of seizure freedom in terms of Engel or ILAE class and duration of follow-up as the meta-analyses, but inconsistent definitions and differing durations meant that we could not adjust for these. Most studies defined seizure freedom as Engel I, potentially compromising results, as this includes patients with ongoing seizures, implying incomplete resection of the epileptogenic zone or multifocal epilepsy.

Meta-analyses improve power, but unless they are hierarchical, lose the granularity of applicability to subgroups. To reduce type I errors, we did not include variables that were significant on unweighted tests, but this can reduce power. Nevertheless, moderate or low-quality evidence from meta-analyses can lead to strong assertions on whether a feature is prognostic (online supplemental reference 13).

Many variables in individual articles of epilepsy surgery outcomes are clinically widely used, contributing to a circular logic, whereby features already considered significant are pooled in meta-analyses. This is why we also discussed unmodelled features.

Whether a feature is of positive or negative prognostic value may be comparable across meta-analyses but due to differing patient populations and seizure-free definitions, diversity of models, unadjusted confounders and unobserved heterogeneity, the magnitude will almost certainly not be, precluding comparisons of effect sizes.^22^ Cochrane-Mantel-Haenszel stratification, multinomial logistic regression or projection to latent space^37^ attempt to adjust for between-feature correlations; nevertheless, this mitigation is limited if important features are omitted. By not fully adjusting for covariates such as focal MRI abnormality or duration of follow-up, incorrect conclusions may be drawn. This limitation is well known^37^ but has not been universally addressed with a definitive set of prognostic features—which was the objective of this study.

As we looked at shared prognostic features across all types of operations and anatomical lobes, our minimum list of EPFs may underidentify variables that may be prognostic for a particular type of operation but not another, such a selective amygdalohippocampectomy as opposed to anterior TL resection. These variables can be identified by further predictive models that adjust for confounders using this list of EPFs. Ultimately interaction terms (deep machine learning models) could adequately stratify seizure freedom.

## Summary and conclusions

Personalised prognostication in epilepsy surgery outcomes has remained elusive and outcomes have not improved with time. We curated features into prognostic and uncertain groups and conclude that more meta-analyses on these are not needed; rather, we need predictive models that quantify their relative contributions to outcomes. We proposed a five-step plan towards personalised seizure freedom predictions and addressed the first two steps in this study. EPFs would be particularly useful in machine learning models of a big-data international collaboration to better predict epilepsy surgery outcomes.

## Data Availability

Data are available upon reasonable request. All data relevant to the study are included in the article or uploaded as supplementary information. All data pertaining to included studies are available in the main manuscript and supplementary materials. The list of titles and abstracts screened for inclusion criteria are available upon request as excel files.
