# Supplementary material for: Multimodal prognostic features of seizure freedom in epilepsy surgery
Source: J Neurol Neurosurg Psychiatry. 2022 Mar 4;93(5):499–508. doi: 10.1136/jnnp-2021-327119 (PMC9016256; doi:10.1136/jnnp-2021-327119)
Supplement: Supplementary data [file jnnp-2021-327119supp001.pdf]

Supplementary Materials

Multimodal Prognostic Features of Seizure-Freedom in Epilepsy Surgery: Towards Personalised Seizure-Freedom Predictions

Ali Alim-Marvasti MRCP[1–4], Vejay Vakharia MRCS[1,4] PhD, John S Duncan DM FRCP FMedSci[1,4]

[1] Department of Clinical and Experimental Epilepsy, UCL Queen Square Institute of Neurology, London WC1N 3BG, UK

Correspondence

Email: a.alim-marvasti@ucl.ac.uk

[2] Department of Medical Physics and Biomedical Engineering, UCL

[3] Wellcome / EPSRC Centre for Interventional and Surgical Sciences (WEISS)

[4] National Hospital for Neurology and Neurosurgery, London, UK

Table of Contents

Supplementary Materials 1

Multimodal Prognostic Features of Seizure-Freedom in Epilepsy Surgery: Towards Personalised Seizure-Freedom Predictions 1

1 Supplementary Methods 5

1.1 Search Strategy 5

1.2 Exclusion Criteria 5

1.3 GRADE Quality of Evidence Scoring 6

1.4 Collection of Effect Sizes 6

1.5 Numbers of studies and participants 6

1.6 Estimating Missing Effect Sizes 7

1.7 Structural Causal Model 7

2 Supplementary Results 8

2.1 Supplementary Table 1: Individual Meta-Analyses of Prognostic Features for Epilepsy Surgery 9

2.1.1 *Chelune, Naugle (20) (1998) 11

2.1.2 Excluded Devous, Thisted (23) (1998) 12

2.1.3 Tonini, Beghi (24) (2004) 13

2.1.4 Excluded as superseded article [4] 16

2.1.5 Willmann, Wennberg (26) (2006) 17

2.1.6 Willmann, Wennberg (27) (2007) 18

2.1.7 Téllez-Zenteno, Ronquillo (5) (2010) 20

2.1.8 Ansari, Tubbs (18) (2010) 21

2.1.9 Ansari, Maher (28) 2010 23

2.1.10 Rowland, Englot (29) (2012) 25

2.1.11 Englot, Wang (30) (2012) 27

2.1.12 Yin, Kang (31) (2013) 30

2.1.13 Englot, Rolston (32) (2013) 31

2.1.14 Zhang, Hu (33) (2013) 34

2.1.15 Josephson, Dykeman (34) (2013) 36

2.1.16 Fallah, Guyatt (35) (2013) 37

2.1.17 Englot, Breshears (37) (2013) 40

2.1.18 Kuang, Yang (38) (2013) 43

2.1.19 Excluded [39] (2013): Review of Reviews 44

2.1.20 Hu, Zhang (40) (2013) 45

2.1.21 Excluded Höller, Kutil (6) (2015) 46

2.1.22 Ibrahim, Morgan (36) (2015) 47
2.1.23 Excluded Bonney, Glenn (41) (2015) 50
2.1.24 Excluded West, Nolan (2) (2015) 50
2.1.25 Ruan, Yu (42) (2015) 51
2.1.26 Cao, Liu (43) (2016) 53
2.1.27 Wang, Zhang (44) (2016) 55
2.1.28 Giridharan, Horn (45) 2016 59
2.1.29 Hu, Zhang (46) 2016 61
2.1.30 Chen and Guo (47) (2016) 63
2.1.31 Excluded Ampie, Choy (48) (2016) 65
2.1.32 Harward, Chen (49) 2017 66
2.1.33 Krucoff, Chan (50) (2017) 67
2.1.34 * Excluded Nevitt, Staba (1) (2017) 70
2.1.35 *Excluded Genetic Stevelink, Sanders (7) (2018) 71
2.1.36 Excluded Pilipović-Dragović, Ristić (9) (2018) 72
2.1.37 Jain, Tomlinson (52) 2018 73
2.1.38 Shan, Fan (53) (2018) 74
2.1.39 Shang-Guan, Wu (54) (2018) 76
2.1.40 Kobulashvili, Kuchukhidze (55) (2018) 78
2.1.41 Harris, Phillips (56) 2019 80
2.1.42 Bjellvi, Olsson (57) (2019) 82
2.1.43 *West, Nevitt (3)* 2019 84
2.1.44 Chen, Chen (58) (2019) 92
2.1.45 Toth, Papp (59) 2019 94
2.1.46 Excluded Pellino, Gencarelli (8) (2020) 95
2.1.47 Widjaja, Jain (60) 2020 96
2.1.48 Excluded Brændholt and Jensen (61) (2020) 99
2.1.49 Lamberink, Otte (62)(2020) 100
2.1.50 Remick, Ibrahim (63) (2020) 105
2.2 Supplementary Table 2: Individual Prognostic Features Across All Meta-Analyses 107
2.3 Supplementary Table 3: Essential Prognostic Features for Epilepsy Surgery (EPF) 161
2.4 Structural Causal Models 187
2.4.1 A Simplified SCM 188
2.4.2 A More Complete SCM 189
References 190

1 Supplementary Methods

1.1 Search Strategy

The free-text search terms used to search PubMed and MEDLINE were:

"epilep*" AND "surg*" AND ("seizure-free" OR "outcome*") AND "meta analys*"

This returned 202 results. With the Humans filter, this reduced to 174, and with a further English language filter, there were 172 articles, of which 111 were meta-analyses. There were a further nine articles included from a non truncated free text search:

"epilepsy" AND "surgery" AND ("seizure-free" OR "outcome") AND "meta analysis*"*

We also used the following MeSH terms:

"Treatment Outcome"[Mesh] AND "surgery" [Subheading] AND "Epilepsy"[Mesh] AND "Meta-Analysis" [Publication Type]

This returned 45 results. Humans and Meta Analyses filters returned the same 45, English language filter reduced this to 43.

Additionally, the same free-text terms were used to search the Cochrane database

Returning 227 Cochrane reviews, of which 104 remained after using the Neurology topic filter. 4 of these were duplicates from PubMed search, leaving 100 unique Cochrane reviews to be screened for inclusion criteria.

After removing duplicated, the above were screened for inclusion criteria based on title and abstract, and if inclusion criteria met, then full-text reviewed for exclusion criteria and prognostic features to extract.

1.2 Exclusion Criteria

- Generalised epilepsy
- Non-resective interventions such as disconnections, neuromodulation, and ablative therapies
- Resections performed primarily for other indications (not directly on outcomes)
- Superseded meta-analyses (2014 Cochrane review replaced in 2017[1] or another 2015 Cochrane review[2] updated in 2019[3], and 2005 article updated in 2010[4, 5])
- Conference presentations and abstracts
- If there was clear and serious concern about risk of bias and unadjusted confounders for any specific feature e.g., attempting meta-analyses using small number of studies/patients or for a feature which was heavily confounded by known prognostic factors.[1, 6-8]
- No meta-analysis attempted or no effect sizes
- Meta-analysis pooling data from only a single study (e.g. surgical techniques[3]).

- Although we included pooled unweighted crude effect sizes of features from individual participant meta-analyses that were non-significant, we excluded such features if they were significant,[9] as unweighted measures can overestimate true effect sizes (Cochran-Mantel-Haenszel confidence intervals are wider).

We did not exclude hemispherectomies or multicentre meta-analyses.

1.3 GRADE Quality of Evidence Scoring

Baseline GRADE quality of each feature from individual meta-analysis were set at “low” (++ out of ++++) by default due to an overwhelming majority of observational studies, except where the majority of constituent studies were randomised or the pooled number of patients were large (>1000) and analyses to investigate bias and/or heterogeneity were performed through sensitivity or subgroup analyses, in which case the preliminary rating for the feature was “moderate” (+++).

+ = Very Low. ++ = Low. +++ = Moderate. ++++ = High.[10-17]

When performing GRADE scoring, regarding indirectness of evidence, although it may be argued that because of the presumed differences in the maturing brain, paediatric and adult epilepsy surgery populations should be investigated separately or as subgroups, we did not rate down for indirectness of evidence if adult and paediatric populations were mixed (Supplementary Table 1 caption).

1.4 Collection of Effect Sizes

If multiple subgroups were reported, e.g., Engel I and Engel Ia for multiple years of follow-up, then we collected the effect size estimates for the strictest outcome (Engel IA) and for the longest duration of follow-up; but where relevant, did consider all the effect sizes when considering inconsistency on the GRADE scale. Where the Cochrane reviews adjusted effect seizes for outcomes, this was quoted. We considered outcomes worse than Engel I or follow-up durations less than 12 months as indirect evidence of good outcomes.

1.5 Numbers of studies and participants

For calculation of medians and IQR of number of individual studies and participants in the main manuscript results section, there were 2 missing datapoints not reported in the meta-analyses. These were imputed using the medians of the rest of the datapoints for numbers of individual articles and numbers of participants.[18, 19] For this calculation, the 8 multicentre study was considered to be from 8 articles [20].

Where there were no numbers for participants or studies for a specific feature in a meta-analysis, total participants across all features in the meta-analysis were used, this, along with the possibility of individual study overlap across meta-analyses, results in the frequency counts of participants and individual studies in main manuscript Tables 2 through 4 to be upper bound estimates.

For summary Table 1 in the main manuscript, we summed all the total individual studies and participants for each category of meta-analysis, irrespective of overlap of individual studies between meta-analyses, and so these are upper bounds of the number of unique individual studies and participants. If a meta-analysis used more than one method, the total number of studies and patients were duplicated for both methods. This was because some studies did not specify exactly

which method was used for which feature and how many studies/participants that involved. The missing values were not imputed for main manuscript Table 1, and were defaulted to zero.

In the few cases of uncertain statements on specific features without forest plots and without quoting effect sizes or the univariate test(s) used, the features were excluded with comments (in red colour in Supplementary Table 1).

1.6 Estimating Missing Effect Sizes

When calculating odds ratios for raw data and their confidence intervals, where the effect size was not provided but the raw data was, these were calculated according to Altman 1991.[21] These are marked by a [c] to indicate the effect size was calculated from the data provided in the literature, and a * where the confidence interval was also estimated.

1.7 Structural Causal Model

The structural causal model outline was designed using dagitty from daggity.net.[22] We used two different levels of complexity, one encompassing all possible relationships and the other simplified to generate causal pathways that are easier to follow diagrammatically. These codes, attached as text files, can be copy-pasted onto http://www.dagitty.net/dags.html to reproduce the causal pathway figures included in the supplementary results below. The R codes used to generate the figures, for reproducibility and future amendment, can also be obtained from pasting the contents of the attached text files to the above website.

2 Supplementary Results

The Table of 44 meta-analyses and their features with GRADE quality of evidence scores are presented in Supplementary Table 1. Supplementary Table 2 shows the same data after collating and reorganising similar features into seven categories.

2.1 Supplementary Table 1: Individual Meta-Analyses of Prognostic Features for Epilepsy Surgery

**Meta-analysis**<br>***Publication year***<br>**Years of individual studies**	**# included Studies, Patients**<br>**Outcomes, Model(s)**	**Feature**<br>**# of total patients with and without**<br>**(# of studies)**	**Population:**<br>**Lobe**<br>**Age**	**Effect Sizes (seizure freedom)**	**Rating the quality of the meta-analysis evidence using the GRADE guidelines** [13]								
					**Risk of Bias or Internal Validity**[1]	**Inconsistency of Results**[2]	**Indirectness of Evidence**[3]	**Imprecision**[4]	**Publication bias**[5]	**Large effect size?**[6]	**"Dose" response?**[6]	**All plausible residual confounding?**[6]	**Quality of the body of evidence (GRADE)**
Author,<br>*Year of publication*<br>Years of literature search	*e.g. 12 studies, 100 patients,*<br>*ILAE 1 or 2 outcomes at least 12months post-surgery*<br>*random-effects model*	*Feature 1*<br>*120 patients in the 7 studies from which feature 1 was extracted*<br>*Feature 2*<br>*200 (9)*	*TLE, FLE…*<br>*Age*<br>*Other*	*RR, OR…*<br>*[95% CI]*	*Heterogenous outcome follow-ups*<br>*Exclusion of known prognostic factors and statistical adjustments*<br>*Selective Reporting*<br>*Serious -1*<br>*V. serious -2*	*Widely spread effect sizes as assessed by point estimates, CI, and statistical tests of heterogeneity.*<br>*Serious -1*<br>*V. serious -2*	*Populations, interventions and/or outcomes being studied differ from those of interest:*<br>*e.g.: unseparated paediatric and adult ages, less than 12 months or more than ILAE 2*	*Large and/ or skewed CI*<br>*Serious -1*<br>*V. serious -2*	*"undetected"*<br>*"suspected"*<br>*-1*<br>*"strongly suspected"*<br>*-2*	*at least a two-fold reduction or increase in risk (OR>4 or <0.25)*<br>*+1*<br>*5-fold or more change in RR (OR >10 or <0.1)*<br>*+2*	*Dose response gradient*<br>*+1*	*All plausible residual confounders or biases would reduce a demonstrated effect, or suggest a spurious effect when results show no effect.*<br>*+1*	*High ++++*<br>*+++ Moderate*<br>*++ Low*<br>*+ Very Low*

Meta-analysis<br>***Publication year***<br>**Years of individual studies**	# included Studies, Patients<br>Outcomes, Follow-up Durations<br>Model(s)	Feature<br># of total patients with and without<br>(# of studies)	Population:<br>Lobe<br>Age	Effect Sizes (seizure freedom)	Rating the quality of the meta-analysis evidence using the GRADE guidelines [13]								
					Risk of Bias or Internal Validity[1]	Inconsistency of Results[2]	Indirectness of Evidence[3]	Imprecision[4]	Publication bias[5]	Large effect size?[6]	“Dose” response?[6]	All plausible residual confounding?[6]	Quality of the body of evidence (GRADE)

2.1.1 *Chelune, Naugle (20) (1998)

Chelune, Naugle (20)<br><br>*1998*<br><br>Baseline Quality: +++	8 centres. 1034 patients<br><br>Outcome: no more than 2 post-operative seizures excluding auras at 6 months or 1 year.<br><br>Individual participant one-way ANOVA.	Low IQ total sample<br><br>1034 (8)<br><br>IQ scores were on average 2.3 lower in not seizure-free group ($p<0.009$)	TLE<br><br>Age ≥16 yrs<br><br>With and without structural lesions	NS RR 0.66<br><br>[0.54, 0.94*]	presence of structural lesions interaction checked	*Multicentre not meta-analysis*	-2 both duration of follow-up and definition of seizure freedom differ	No CI provided, but could be estimated from data presented	-1 suspected, untested		+1<br><br>Higher seizure-freedom rates in higher IQs (their table 3)	+1<br><br>Adjusted for lesions	++
		Low presurgical IQ ≤75 in patients with structural lesions other than HS (cf high IQ and lesional)<br><br>150 lesional (8)		RR 0.26<br><br>[0.14, 0.50]*								Absence of significant interaction with centres, or duration of epilepsy	+

*Found though other sources (see PRISMA flowchart)

2.1.2 Excluded Devous, Thisted (23) (1998)

Excluded as not directly on outcomes, and the data presented on outcomes are proportions of seizure freedom with SPECT, but no other baseline to compare to derive effect sizes. Abstract only.

2.1.3 Tonini, Beghi (24) (2004)

**Meta-analysis**<br><br>***Publication year***<br><br>**Years of individual studies**	**# included Studies, Patients**<br><br>**Outcomes, Follow-up Durations**<br><br>**Model(s)**	**Feature**<br><br>**# of total patients with and without**<br><br>**(# of studies)**	**Population:**<br><br>**Lobe**<br><br>**Age**	**Effect Sizes (seizure freedom)**	**Rating the quality of the meta-analysis evidence using the GRADE guidelines [13]**								
					**Risk of Bias or Internal Validity[1]**	**Inconsistency of Results[2]**	**Indirectness of Evidence[3]**	**Imprecision[4]**	**Publication bias[5]**	**Large effect size?[6]**	**"Dose" response?[6]**	**All plausible residual confounding?[6]**	**Quality of the body of evidence (GRADE)**
Tonini, Beghi (24)<br><br>(2004)<br><br>1984-2001<br><br>Odds ratios inverted so that OR>1 Represents good outcomes<br><br>+++	47 studies<br><br>10 were prospective<br><br>2 were both retro and prospective<br><br>total of 3,511 patients<br><br>>12 months follow up<br><br>Fixed-effects and mixed effects	febrile seizures<br><br>(5)	TL and ET<br><br>Subgroup analysis showed no change when only TL was considered<br><br>Children and adults from 1 through to 86 years	OR 2.08 [1.2, 3.7]	sample size of at least 30 patients, MRI performed in at least 90% of cases, English, Italian, French, German, or Spanish	Q=7.9, p=0.093	mixed populations<br><br>-1<br><br>Engel outcomes in 22 studies and other definitions in 25						+
		mesial temporal sclerosis in TLE<br><br>(15) +++		OR 2.13 [1.57, 2.86]		Q=21.9, p=0.082							++
		Tumours<br><br>(13) +++		OR 1.74 [1.25, 2.5]		Q=19.3, p=0.08							++

		abnormal MRI<br><br>(9) +++		OR 2.27 [1.54, 3.45]		One outlier with poor results but wide CI<br><br>Q=4.9, p=0.768							++
		extensive surgical resection<br><br>(10) +++		OR 4.27 [2.06, 8.85]		Q=26.9,p=0.001 → used random effects							++
		EEG/MRI concordance<br><br>(6)		OR 2.36 [1.07, 5.26]		Heterogenous<br><br>Q=11.4, p=0.044<br><br>→ used random effects							+
		Post operative discharges<br><br>(3)		OR 0.28 [0.08, 0.95]		-1<br><br>@bias :<br><br>Only 3 studies<br><br>Heterogenous Q=6.7, p=0.035<br><br>→ random effects							+
		Intracranial monitoring<br><br>(6)		OR 0.37 [0.22, 0.63]		Q=3,p=0.7							+
		Neuro-migrational,defects<br><br>(6)		NS<br><br>OR 0.66 [0.42, 1.03]		Q=9.8, p=0.08							+
		CNS infections<br><br>(2)		NS<br><br>OR 0.73 [0.29, 1.82]		-1<br><br>@Bias:<br><br>Only 2 studies<br><br>Q=2.1, p=0.146							+

		Vascular disorders<br>(3)		NS<br>OR 0.66 [0.30, 1.46]		-1<br>@bias: 3 studies<br>Q=945, p=0.6							+
		interictal spikes<br>(3)		NS<br>OR 1.82 [0.86, 3.88]		-1<br>@bias: 3 studies		-1<br>skewed					+
		side of resection<br>(4)		NS<br>OR 0.85 [0.54, 1.34]									+

2.1.4 Excluded as superseded article [4]

See exclusion criteria. This article was superseded by [25]

2.1.5 Willmann, Wennberg (26) (2006)

**Meta-analysis** *Publication year* **Years of individual studies**	**# included Studies, Patients** **Outcomes, Follow-up Durations** **Model(s)**	**Feature** **# of total patients with and without** **(# of studies)**	**Population:** **Lobe** **Age**	**Effect Sizes (seizure freedom)**	**Rating the quality of the meta-analysis evidence using the GRADE guidelines [13]**								
					**Risk of Bias or Internal Validity[1]**	**Inconsistency of Results[2]**	**Indirectness of Evidence[3]**	**Imprecision[4]**	**Publication bias[5]**	**Large effect size?[6]**	**"Dose" response?[6]**	**All plausible residual confounding?[6]**	**Quality of the body of evidence (GRADE)**
Willmann, Wennberg (26) (2006) 1992-2003	22 studies 121 pts Engel 1 Studies exclusively reporting on patients with brain tumors or on children were excluded Engel I Unclear if fixed or mixed effects but likely fixed effects CMH	^{1}H spectroscopy: ipsilateral magnetic spectroscopy abnormality (ipsilateral to lobe of resection)	TLE 3-66 years old	OR 4.9 [1.97–12.17]	Fifteen centers performed chemical shift imaging and seven centers used single-voxel spectroscopy. Most studies were obtained at 1.5 T	Q=2.7	-2 The EZ was mostly defined by EEG data	-1 Large CI		PPV = 82% +1 OR>4			+
		Ipsilateral magnetic spectroscopy	Non-lesional MRI and TLE	NS									+

2.1.6 Willmann, Wennberg (27) (2007)

**Meta-analysis** ***Publication year*** **Years of individual studies**	**# included Studies, Patients** **Outcomes, Follow-up Durations** **Model(s)**	**Feature** **# of total patients with and without** **(# of studies)**	**Population:** **Lobe** **Age**	**Effect Sizes (seizure freedom)**	**Rating the quality of the meta-analysis evidence using the GRADE guidelines** [13]								
					**Risk of Bias or Internal Validity**[1]	**Inconsistency of Results**[2]	**Indirectness of Evidence**[3]	**Imprecision**[4]	**Publication bias**[5]	**Large effect size?**[6]	**"Dose" response?**[6]	**All plausible residual confounding?**[6]	**Quality of the body of evidence (GRADE)**
Willmann, Wennberg (27) (2007) 1992-2006 Studies exclusively reporting on patients with brain tumors or on children were excluded.	46 articles (11 TLE and ET, 35 TLE) 153 patients Follow up >12 months Unweighted crude odds ratios	FDG-PET (46)	TLE and ET Adults	NS Unweighted crude	-1 None of the odds ratios of any test combination was significant The analyses were complicated by significant differences in study design and often by lack of precise patient data. the tracer injection dose from 1 to 15 mCi, and the time for data acquisition after tracer injection from 5 to 60 min		PET does not appear to add value in patients localized by ictal scalp EEG and MRI.						+
		FDG-PET (35)	TLE Adults	NS Unweighted crude									+
		Left vs right temporal	TLE Adults	NS									++

		lobe surgery		Unweighted crude<br><br>OR 0.569 [0.26, 1.24]									

2.1.7 Téllez-Zenteno, Ronquillo (5) (2010)

Téllez-Zenteno, Ronquillo (5)<br><br>(2010)<br><br>1995-2007<br><br>+++	40 studies<br><br>3557 (2860 lesional and 697 non-lesional cases)<br><br>Engel I<br><br>>1 year follow-up<br><br>Random-effects	Lesional vs non-lesional<br><br>TL lesional vs TL non-lesional<br><br>ET lesion vs ET non lesion	Adults and children	OR 2.5 [2.1, 3.0]<br><br>RR 1.4<br><br>OR 2.7 [2.1, 3.5]<br><br>OR 2.9 [1.6, 5.1]	-1<br><br>Heterogenous SF definitions.<br><br>But similar results in subgroups: adults, children, TL, ET.<br><br>Two studies favoured non-lesional epilepsy	Q=35.6, p=0.43	They also investigated whether lesion definition by MRI or histopathology made a difference – it didn't		? non lesional were significantly higher in ET cases (45%) than in TL (24%)				++

2.1.8 Ansari, Tubbs (18) (2010)

Meta-analysis *Publication year* Years of individual studies	# included Studies, Patients Outcomes, Follow-up Durations Model(s)	Feature # of total patients with and without (# of studies)	Population: Lobe Age	Effect Sizes (seizure freedom)	Rating the quality of the meta-analysis evidence using the GRADE guidelines [13]								
					Risk of Bias or Internal Validity[1]	Inconsistency of Results[2]	Indirectness of Evidence[3]	Imprecision[4]	Publication bias[5]	Large effect size?[6]	"Dose" response?[6]	All plausible residual confounding?[6]	Quality of the body of evidence (GRADE)
Ansari, Tubbs (18) (2010)<br>>1990<br>+	?<br>131 patients<br>Engel classification<br>Outcome at 1 year<br>Fisher's exact and ANOVA	age at onset	adults extratemporal non lesional	NS	-1<br>Small sample sizes form multiple centres, heterogenous outcome reporting								+
		age at surgery		NS									+
		epilepsy duration		NS									+
		focal vs generalised seizures<br>62 ()		NS									+
		FCD or gliosis<br>115 ()		NS									+
		Frontal, central, posterior or		NS									+

		other resections<br><br>81 ()											
		Lateralisation		NS									+
		Abnormal MRI<br><br>61 ()		NS									+ excluded as ET non lesional population
		Intracranial monitoring<br><br>108 ()		NS									+

2.1.9 Ansari, Maher (28) 2010

**Meta-analysis**<br><br>***Publication year***<br><br>**Years of individual studies**	**# included Studies, Patients**<br><br>**Outcomes, Follow-up Durations**<br><br>**Model(s)**	**Feature**<br><br>**# of total patients with and without**<br><br>**(# of studies)**	**Population:**<br><br>**Lobe**<br><br>**Age**	**Effect Sizes (seizure freedom)**	**Rating the quality of the meta-analysis evidence using the GRADE guidelines [13]**								
					**Risk of Bias or Internal Validity[1]**	**Inconsistency of Results[2]**	**Indirectness of Evidence[3]**	**Imprecision[4]**	**Publication bias[5]**	**Large effect size?[6]**	**"Dose" response?[6]**	**All plausible residual confounding?[6]**	**Quality of the body of evidence (GRADE)**
Ansari, Maher (28) 2010<br><br>**1990-2009**	17 studies<br><br>95 pts<br><br>Engel I<br><br>Univariate ANOVA and Fisher's exact tests	Age at seizure onset<br><br><95 (<17)	ET nonlesional children	NS[u]			?outcome duration of follow up		-1<br><br>suspected				+
		Mean duration of epilepsy <95 (<17)		NS[u]									+
		At age surgery and outcome <95 (<17)		NS[u]	Report as marginally significant but NS p =0.073								+
		Seizure semiology grouped into complex partial, generalized, infantile spasms and other which included simple partial and mixed types.<br><br>65 (?)		Did not perform CMH or meta-analysis besides univariate Fisher's	No direct results between groups								Rejected as significant on univariate tests only
		Histopathology: cortical		As above									As above

		dysplasia, gliosis, other. (included neuronal loss, encephalitis, polymicrogyria, ulegyria, chronic inflammation, and "normal.")											
		Types of surgery (frontal, posterior and other)		NS[u]	Report as marginally significant but NS p =0.059 on univariate, which would not be significant on multivariate / CMH / Bonferronis								+
		Seizure lateralization		NS[u]									+
		Abnormal MRI		NS[u]	High risk of bias due to the selection of non lesional cases (!)								+ rejected as it was ET non lesional…
		Intracranial monitoring		NS[u]									+

2.1.10 Rowland, Englot (29) (2012)

**Meta-analysis**<br><br>***Publication year***<br><br>**Years of individual studies**	**# included Studies, Patients**<br><br>**Outcomes, Follow-up Durations**<br><br>**Model(s)**	**Feature**<br><br>**# of total patients with and without**<br><br>**(# of studies)**	**Population:**<br><br>**Lobe**<br><br>**Age**	**Effect Sizes (seizure freedom)**	**Rating the quality of the meta-analysis evidence using the GRADE guidelines [13]**								
					**Risk of Bias or Internal Validity[1]**	**Inconsistency of Results[2]**	**Indirectness of Evidence[3]**	**Imprecision[4]**	**Publication bias[5]**	**Large effect size?[6]**	**“Dose” response?[6]**	**All plausible residual confounding?[6]**	**Quality of the body of evidence (GRADE)**
Rowland, Englot (29) (2012)<br><br>1971 to 2011<br><br>+++	37 studies<br><br>2,014 patients<br><br>Engel outcomes<br><br>random-effects meta-analysis	Partial (focal) vs generalised seizures<br><br>(10)	Adults and Children with FCD	OR 1.46<br><br>[1.18, 1.82]					-1<br><br>No funnel plots/trim fill etc<br><br>Only 1 forest plot shown for complete resection				++
		Temporal lobe resections<br><br>(20)		OR 1.35<br><br>[1.13, 1.61]									++
		Abnormal MRI<br><br>(14)		OR 1.67<br><br>[1.33, 2.16]									++
		FCD Type II (Palmini)<br><br>(17)		OR 1.38<br><br>[1.22, 1.57]									++

		Complete resection<br><br>(15)		OR 3.91<br><br>[3.03, 5.32]									++
		Age (<18 yrs vs >18yrs)<br><br>(13)		NS<br><br>OR 1.14 [0.96, 1.35]									++
		Unilateral EEG vs bilateral ictal EEG<br><br>(10)		NS<br><br>OR 1.03 [0.82, 1.31]									++

2.1.11 Englot, Wang (30) (2012)

**Meta-analysis**<br><br>***Publication year***<br><br>**Years of individual studies**	**# included Studies, Patients**<br><br>**Outcomes, Follow-up Durations**<br><br>**Model(s)**	**Feature**<br><br>**# of total patients with and without**<br><br>**(# of studies)**	**Population:**<br><br>**Lobe**<br><br>**Age**	**Effect Sizes (seizure freedom)**	**Rating the quality of the meta-analysis evidence using the GRADE guidelines [13]**								
					**Risk of Bias or Internal Validity[1]**	**Inconsistency of Results[2]**	**Indirectness of Evidence[3]**	**Imprecision[4]**	**Publication bias[5]**	**Large effect size?[6]**	**"Dose" response?[6]**	**All plausible residual confounding?[6]**	**Quality of the body of evidence (GRADE)**
Englot, Wang (30) (2012)<br><br>1990-2010<br><br>Significance set at 0.02<br><br>+++	21 studies<br><br>1,199 patients<br><br>Engel I >48 months<br><br>Chi-squared/t-tests then random-effects model	Lesional<br><br>825 (16)<br><br>++	FLE<br><br>Adults and Children	RR 1.67, [1.36, 28.6]	Lesion: tumour, CD, or other lesion vs non-lesional: traumatic, infectious			-1<br><br>Wide CI	Funnel plots: undetected (not shown)				+
		abnormal pre-operative MRI<br><br>627<br><br>(14)		RR 1.64, [1.32, 2.08]									++
		localised frontal resections (vs more extensive frontal +/- extrafrontal resections)<br><br>651 (11)		RR 1.71, [1.26, 2.43]									++

		complete lesion excision<br><br>345 (7)		RR 1.99, [1.47, 2.84]									++
		Focal vs generalized seizure semiology<br><br>269 ()		NS P=0.05<br><br>magnitude not provided				-1<br><br>Magnitude not provided and p value borderlin					+
		Age <18 yrs vs >18 yrs		NS<br><br>43% vs 54% p=0.22									++
		Duration of epilepsy		NS	-1<br><br>Limited info								+
		Seizure frequency		NS									+
		side of surgery		NS									+
		gender		NS p0.99<br><br>Males 53% females 54%									++
		intracranial EEG performed		NS	-1<br><br>Limited info								+
		LTM with video-EEG		NS									+

		localised ictal EEG		NS									+
		lateralised interictal EEG		NS									+
		focal PET abnormality		NS									+
		intraoperative EcOG<br><br>1024 ()		NS p=0.14<br><br>Pooled ind particicpant OR[c] 1.23 [0.95, 1.62]									+++

2.1.12 Yin, Kang (31) (2013)

Meta-analysis<br>*Publication year*<br>Years of individual studies	# included Studies, Patients<br>Outcomes, Follow-up Durations<br>Model(s)	Feature<br># of total patients with and without<br>(# of studies)	Population:<br>Lobe<br>Age	Effect Sizes (seizure freedom)	Rating the quality of the meta-analysis evidence using the GRADE guidelines [13]								
					Risk of Bias or Internal Validity[1]	Inconsistency of Results[2]	Indirectness of Evidence[3]	Imprecision[4]	Publication bias[5]	Large effect size?[6]	"Dose" response?[6]	All plausible residual confounding?[6]	Quality of the body of evidence (GRADE)
Yin, Kang (31) (2013)<br>1995-2012	22 studies<br>2171 patients<br>>6 months follow up: majority over 1 year, 3 studies <1 year<br>Fixed-effects	lesion on neuroimaging	TL and ET<br>Children and adults	OR 2.03 [1.67, 2.47]	English and Chinese studies	I^2=0%	-1<br>short follow ups: 3 studies <1 year follow up		undetected				++
	11 studies<br>1228 pts<br>+++	lesion in temporal resection<br>(11)	TLE<br>Children and adults	OR 1.76 [1.34, 2.32]		I^2=19%, p=0.26<br>Note that the caption to Fig 3 doesn't fit with the p and I-squared values in the forest plot							++
	5 studies<br>203 pts	lesion in extratemporal resection<br>(5)	ET<br>Children and adults	OR 2.88 [1.53, 5.43]		I^2=0%							+

2.1.13 Englot, Rolston (32) (2013)

Meta-analysis<br>*Publication year*<br>Years of individual studies	# included Studies, Patients<br>Outcomes, Follow-up Durations<br>Model(s)	Feature<br># of total patients with and without<br>(# of studies)	Population:<br>Lobe<br>Age	Effect Sizes (seizure freedom)	Rating the quality of the meta-analysis evidence using the GRADE guidelines [13]								
					Risk of Bias or Internal Validity[1]	Inconsistency of Results[2]	Indirectness of Evidence[3]	Imprecision[4]	Publication bias[5]	Large effect size?[6]	"Dose" response?[6]	All plausible residual confounding?[6]	Quality of the body of evidence (GRADE)
Englot, Rolston (32) (2013)<br>1993-2012<br>++	36 studies<br>1,318 patients<br>Engel I minimum of 1 year<br>random-effects model after significance on univariate tests.	lesional<br>(FCD, tumour, tuber, vascular malformation) vs non lesional (included HS, trauma, infection)<br>945 (29)	Paediatric<br>TL	OR 1.08 [1.02, 1.15]			-1<br>Lesional excludes HS		Funnel plots: undetected				+
		focal seizures (partial) vs generalised<br>425 (11)		OR 1.36 [1.20, 1.56]									++
		without daily seizures<br>103 (5)		none given<br>OR[c] individual participant 2.98 [1.24, 7.16]]	-1<br>small number of studies reported this: no formal CMH Meta-Analysis attempted.								+
		abnormal MRI<br>802 (26)		OR 1.27 [1.16, 1.40]									++

		Age (17)		NS t-test	Individual participant pooling of data, chi-squared tests or paired t tests failed to show significant and so CMH random effects not performed								+
		Gender/sex: male vs female<br><br>553 (14)		NS<br><br>Pooled individual participant (crude)<br><br>OR[c] 1.22 [0.90, 1.85]									++
		mean duration of epilepsy (12)		NS<br><br>t-test									++
		localising ictal EEG<br><br>445 (14)		NS<br><br>Pooled crude individual participant<br><br>OR[c] 1.23 [0.73, 2.06]									++
		side of surgery<br><br>537 (15)		NS<br><br>OR[c] crude 1.07 [0.72, 1.60]									++
		use of electrocorticography (ECoG)<br><br>462 (13)		NS<br><br>OR[c] crude 1.31 [0.84, 2.04]									++
		Type of surgery: ATL vs lesionectomy vs		"NS"									Excluded as no data looking at

		lesionectomy plus additional vs SAH											the substrata CMH. In the text mentioned and in table p=0.02 but no meta analysis available.

2.1.14 Zhang, Hu (33) (2013)

**Meta-analysis** ***Publication year*** **Years of individual studies**	**# included Studies, Patients** **Outcomes, Follow-up Durations** **Model(s)**	**Feature** **# of total patients with and without** **(# of studies)**	**Population:** **Lobe** **Age**	**Effect Sizes (seizure freedom)**	**Rating the quality of the meta-analysis evidence using the GRADE guidelines [13]**								
					**Risk of Bias or Internal Validity[1]**	**Inconsistency of Results[2]**	**Indirectness of Evidence[3]**	**Imprecision[4]**	**Publication bias[5]**	**Large effect size?[6]**	**"Dose" response?[6]**	**All plausible residual confounding?[6]**	**Quality of the body of evidence (GRADE)**
Zhang, Hu (33) (2013) 1990-2012	13 studies 229 patients Engel as reported Mean or median follow up of>12months Random and fixed-effects models	tuberectomy vs lobectomy 189 (10)	tuberous sclerosis	OR 0.51 [0.27, 0.99]		I^2=0% as few numbers	-1 Heterogenous definitions of seizure freedom without sensitivity analyses	-1 CI approaches OR of 1	-1 No funnel plots				+
		seizure onset before 12 months of age 200 (10)		OR 0.47 [0.24, 0.92]		I^2=0% as few numbers							+
		unilateral ictal EEG 159 (8)		OR 2.48 [1.17, 5.24]		I^2=0% as few numbers							+
		unilateral interictal EEG		OR 2.42 [1.11, 5.27]		I^2=0% as few numbers							+

		127 (6)											
		<5yrs vs >5yrs of age (at surgery)<br><br>194 (11)		NS<br><br>OR 1.05 [0.58, 1.88]									+
		Gender: males vs females<br><br>186 (10)		NS<br><br>OR 0.94 [0.52, 1.71]									+
		partial vs generalised<br><br>65 (6)		NS<br><br>OR 1.15 [0.42, 3.11]									+
		Normal vs mental retardation<br><br>108 (4)		NS<br><br>OR 1.36 [0.61, 3.05]		@bias: no definition of “mental retardation”							+
		number of cortical tubers <=4 vs >4<br><br>105 (4)		NS<br><br>OR 1.12 [0.49, 2.57]									+
		intracranial EEG performed vs not preformed<br><br>144 (7)		NS<br><br>OR 1.6 [0.76, 3.37]									+
		Infantile spasms (IS)<br><br>157 (7)		OR 0.45 [0.24, 0.85		I^2=45%							+

2.1.15 Josephson, Dykeman (34) (2013)

Meta-analysis<br>*Publication year*<br>Years of individual studies	# included Studies, Patients<br>Outcomes, Follow-up Durations<br>Model(s)	Feature<br># of total patients with and without<br>(# of studies)	Population:<br>Lobe<br>Age	Effect Sizes (seizure freedom)	Rating the quality of the meta-analysis evidence using the GRADE guidelines [13]								
					Risk of Bias or Internal Validity[1]	Inconsistency of Results[2]	Indirectness of Evidence[3]	Imprecision[4]	Publication bias[5]	Large effect size?[6]	"Dose" response?[6]	All plausible residual confounding?[6]	Quality of the body of evidence (GRADE)
Josephson, Dykeman (34) (2013)	11 studies<br>1,203 patients<br>Engel I outcomes but also Engel I and II<br>fixed-effects	ATLR vs SAH<br>+++	TLE<br>Children and adults	RR 1.32<br>[1.12, 1.57]<br>Random effects:<br>RR 1.36 [1.09, 1.7]	The result remained significant when 2 studies that contained fewer than 15 participants in at least 1 arm were excluded and in analyses restricted to hippocampal sclerosis and when a study specific to paediatric patients was excluded	(I^2= 29%; df =10, p=0.17	children and adults – but also excluded paediatric only study and results were very similar		undetected	summary risk difference (8%,<br>95% CI 3%–14%) translates to an NNT of 13 (95% CI<br>7–33) for 1 additional patient to achieve an Engel Class I<br>outcome following ATL			+++
	10 studies<br>1092 pts	ATL vs SAH<br>+++	TLE and HS subgroup<br>Children and adults	RR 1.26 [1.05, 1.51].		I^2=0%							+++

2.1.16 Fallah, Guyatt (35) (2013)

**Meta-analysis**<br><br>***Publication year***<br><br>**Years of individual studies**	**# included Studies, Patients**<br><br>**Outcomes, Follow-up Durations**<br><br>**Model(s)**	**Feature**<br><br>**# of total patients with and without**<br><br>**(# of studies)**	**Population:**<br><br>**Lobe**<br><br>**Age**	**Effect Sizes (seizure freedom)**	**Rating the quality of the meta-analysis evidence using the GRADE guidelines [13]**								
					**Risk of Bias or Internal Validity[1]**	**Inconsistency of Results[2]**	**Indirectness of Evidence[3]**	**Imprecision[4]**	**Publication bias[5]**	**Large effect size?[6]**	**"Dose" response?[6]**	**All plausible residual confounding?[6]**	**Quality of the body of evidence (GRADE)**
Fallah, Guyatt (35) (2013)<br><br>Same data as in Ibrahim, Morgan (36)	20 articles<br><br>181 pts<br><br>Engel I<br><br>Univariate meta-analysis<br><br>Bivariate logistic regression for each eligible independent variable, adjusting for the maximum length of follow-up.	absence of generalised semiology	Tuberous Sclerosis<br><br>At least 90% less than 19 years old	OR = 3.1 [1.2, 8.2]	-1<br><br>Although a list of biologically plausible predictors was developed a priori; due to the small sample sizes of individual studies (median: 7; range 3–25 patients), and the variable inclusion of predictors across studies, unable to conduct a multivariable analysis or adjust	See publication bias comments on inability to assess hetergoeneity			-1<br><br>Suspected but "because of the very small number of participants per study, we could not assess between-study heterogeneity or publication bias."				+
		no or mild developmental delay		OR = 7.3 [2.1–24.7]				-1<br><br>Wide CI		+1 OR>4			++
		Unifocal ictal scalp EEG abnormality		OR = 3.21, [1.35–7.58]									+

		MRI/EEG concordance		OR = 4.9, [1.8–13.5]				-1 Wide CI		+1 OR>4			+
													+
		Gender (female)		NS OR 1.09 [0.48, 2.48]									+
		age at seizure onset (Log base 10)		NS OR 1.52 [0.77, 2.99]									+
		Pre-op seizure frequency (Log base 10)		NS OR 2.3 [0.34, 15.51]									+
		infantile spasms		NS OR 0.84 [0.35, 2.03]									+
		age at surgery (Log base 10)		NS OR 1.21 [0.56, 2.62]									+
		preoperative IQ		NS OR 1.01 [0.94, 1.08]									+
		less tuber burden		NS OR 1.01 [0.96, 1.07]									+

		No or unifocal interictal scalp EEG abnormality		NS OR 1.54 [0.73, 3.26]		-1<br><br>@Bias: unusual feature dichotomization<br><br>See publication bias comments on inability to assess hetergoeneity							+

2.1.17 Englot, Breshears (37) (2013)

**Meta-analysis**<br><br>***Publication year***<br><br>**Years of individual studies**	**# included Studies, Patients**<br><br>**Outcomes, Follow-up Durations**<br><br>**Model(s)**	**Feature**<br><br>**# of total patients with and without**<br><br>**(# of studies)**	**Population:**<br><br>**Lobe**<br><br>**Age**	**Effect Sizes (seizure freedom)**	**Rating the quality of the meta-analysis evidence using the GRADE guidelines [13]**								
					**Risk of Bias or Internal Validity[1]**	**Inconsistency of Results[2]**	**Indirectness of Evidence[3]**	**Imprecision[4]**	**Publication bias[5]**	**Large effect size?[6]**	**"Dose" response?[6]**	**All plausible residual confounding?[6]**	**Quality of the body of evidence (GRADE)**
Englot, Breshears (37) 2013	36 studies<br><br>1259 patients<br><br>Engel I<br><br>Chi-squared / unpaired t-tests significance correction for multiple comparisons at 0.02. Then fixed effects.	Shorter epilepsy duration (≤ 7 years, the median value in this study)<br><br><1259 (8)	Extratemporal paediatric population	OR 1.52 [1.07, 2.14]	-1 suspected as no adjustments reported	Friedman and Kendall's W tests and Cochrane Q statistics			Not detected on funnel plots<br><br>Although to our eyes figure 2 C shows possible asymmetry for partial vs generalized seizure semiology				+
		Lesional epilepsy (aetiology)<br><br>695 (28)		OR 1.34, [1.19, 1.49									+
		absence of generalized seizures<br><br>206 (11)		OR 1.61 [1.18, 2.35]									+
		localizing ictal electroencephalographic findings<br><br>226 (13)		OR 1.55 [1.24, 1.93]									+
		Mean age at surgery		NS[u] OR									+

BMJ Publishing Group Limited (BMJ) disclaims all liability and responsibility arising from any reliance placed on this supplemental material which has been supplied by the author(s)

		<1259 (17)											
		Gender male vs female<br>303 (15)		NS[u] OR[c*] 1.16 [0.74, 1.83]									+
		Daily seizures (yes vs no)<br>158 (4)		NS<br>OR[uc*] 0.54 [0.28, 1.07]									+
		Abnormal preoperative MRI<br>506 (23)		NS<br>OR[uc*] 1.44 [0.98, 2.12]									+
		Interictal EEG lateralizing<br>Vs non-lateralizing<br>130 (10)		NS<br>OR[uc*] 2.22 [0.98, 5.05]									+
		Operative variables, surgical lobe (frontal, parietal, Rolandic, occipital, multilobed)<br>537 (26)		NS[u]<br>See their table 1									+
		Surgery side left vs right<br>326 (15)		NS<br>OR[uc*] 0.99 [0.64, 1.53]									+

		Extent of lesionectomy: gross-total (complete) vs subtotal<br><br>75 (5)		NS^{u}<br><br>OR^{uc*} 13.89 [3.30, 58.38]<br><br>→ meta analysis weighted: not shown or reported on the article				-1 few numbers and wide CI					Rejected as significant only on univariate unweighted analysis
		ECoG used vs not used<br><br>433 (20)		NS^{u}<br><br>OR^{uc*} 0.77 [0.50, 1.19]									+

2.1.18 Kuang, Yang (38) (2013)

Meta-analysis<br>*Publication year*<br>Years of individual studies	# included Studies, Patients<br>Outcomes, Follow-up Durations<br>Model(s)	Feature<br># of total patients with and without<br>(# of studies)	Population:<br>Lobe<br>Age	Effect Sizes (seizure freedom)	Rating the quality of the meta-analysis evidence using the GRADE guidelines [13]								
					Risk of Bias or Internal Validity[1]	Inconsistency of Results[2]	Indirectness of Evidence[3]	Imprecision[4]	Publication bias[5]	Large effect size?[6]	"Dose" response?[6]	All plausible residual confounding?[6]	Quality of the body of evidence (GRADE)
Kuang, Yang (38) (2013)<br>1989 - 2008	6 studies: RCTs<br>626 patients<br>Engel I 1 year post operatively<br>Fixed effect	ATL vs SAH<br>626 (6)	TLE	NS<br>RR 1.01 [0.54, 1.09]		No evidence of inconsistency found							

2.1.19 Excluded [39] (2013): Review of Reviews

10 reviews or meta-analyses identified. No formal meta-analysis. Qualitative synthesis.

Table 6 includes common predictors of seizure outcome for

- lesional and non-lesional TLE,
- lesional ET, and
- Tuberous Sclerosis:

Positive Predictors:

- Lesional, abnormal MRI, partial seizures, complete resection
- As above
- No or mild developmental delay, unifocal ictal EEG abnormality, extensive resection (lobotomy)

Negative Predictors:

- Nonlesional epilepsy, poorly localised EEG, bilateral or multifocal MRI lesions, generalized seizures, FCD type 1, need for ictal EEG, incomplete resection, abnormal post-operative EEG
- Generalized seizures and as above
- Severe developmental delay, corpus callosotomy or tuberectomy

Non-Prognostic:

- Age at surgery, sex, duration of epilepsy, ictal EEG, side of surgery
- Seizure frequency and as above
- Infantile spasms, invasive EEG, PET findings, tuber burden

However, no common predictors of seizure outcome were identified in nonlesional ETLE.

2.1.20 Hu, Zhang (40) (2013)

**Meta-analysis**<br><br>***Publication year***<br><br>**Years of individual studies**	**# included Studies, Patients**<br><br>**Outcomes, Follow-up Durations**<br><br>**Model(s)**	**Feature**<br><br>**# of total patients with and without**<br><br>**(# of studies)**	**Population:**<br><br>**Lobe**<br><br>**Age**	**Effect Sizes (seizure freedom)**	**Rating the quality of the meta-analysis evidence using the GRADE guidelines [13]**								
					**Risk of Bias or Internal Validity[1]**	**Inconsistency of Results[2]**	**Indirectness of Evidence[3]**	**Imprecision[4]**	**Publication bias[5]**	**Large effect size?[6]**	**"Dose" response?[6]**	**All plausible residual confounding?[6]**	**Quality of the body of evidence (GRADE)**
Hu, Zhang (40) (2013)<br><br>1993-2012	13 studies<br><br>1397 patients (686 total SAH, 711 total ATL)<br><br>Engel I<br><br>Mean or median follow up >1 year<br><br>Fixed or random effects	SAH vs ATL	TLE	Overall OR 0.65 [0.51, 0.82]<br><br>Transsylvian SAH OR (4)<br><br>0.60 [0.41, 0.87]<br><br>Transcortical SAH OR (5)<br><br>0.68 [0.49, 0.96]<br><br>Unknown or multiple approaches OR (2)<br><br>0.7 [0.25, 1.95]	Also included subgroups of transcortical or transsylvian SAH.<br><br>Used NOS scale.<br><br>The sensitivity analysis demonstrated that the significance of seizure outcome was not altered with the exclusion of low-quality studies (OR 0.57 [95% CI 0.43–0.76], p = 0.0001).	Trans-sylvian approach I^2 = 74% and p =0.009<br><br>Overall I^2 = 43%, p= 0.06<br><br>Otherwise, no significant heterogeneity<br><br>Insufficient to mark down given the subgroup analysis		Only 2 studies for mixed approaches and hence may lack power with wide CI, whereas the other subgroup analyses show clear benefit for ATL	-1<br><br>Mild bias on funnel plot, did not further investigate or use trim and fill				++

2.1.21 Excluded Höller, Kutil (6) (2015)

**Meta-analysis**<br><br>***Publication year***<br><br>**Years of individual studies**	**# included Studies, Patients**<br><br>**Outcomes, Follow-up Durations**<br><br>**Model(s)**	**Feature**<br><br>**# of total patients with and without**<br><br>**(# of studies)**	**Population:**<br><br>**Lobe**<br><br>**Age**	**Effect Sizes (seizure freedom)**	**Rating the quality of the meta-analysis evidence using the GRADE guidelines [13]**								
					**Risk of Bias or Internal Validity[1]**	**Inconsistency of Results[2]**	**Indirectness of Evidence[3]**	**Imprecision[4]**	**Publication bias[5]**	**Large effect size?[6]**	**"Dose" response?[6]**	**All plausible residual confounding?[6]**	**Quality of the body of evidence (GRADE)**
Höller, Kutil (6) (2015)	11 studies<br><br>ILAE 1/Engel Ia<br><br>Random-effects model	HFO resection ratios (:= proportion of HFO electrodes in the resected lobe, compared to total number of HFO electrodes).	Children and adults	Difference between SF and NSF resection ratios quoted:<br><br>Ripples<br><br>0.18 [0.1, 0.27]<br><br>Fast ripples 0.17 [0.01, 0.33]	-2<br><br>10 studies looked at ripples (80-200Hz) while 7 looked at fast ripples (>200Hz)<br><br>Diff of HFO ratio quoted, not OR/RR of outcomes.<br><br>No adjustments.	Q-statistic significant (p=0.025, for ripples and p<0.001 for fast ripples) with I^2 = 53%, 77% respectively.	-1<br><br>Difference of resection ratios between SF and NSF groups of 0.184 and 0.167, respectively.	In 9 of 10 studies resection ratio was higher for SF but in 5 of the 9 ripples studies the CI overlapped with zero<br><br>5 of 7 fast ripples studies resection ratio > for SF; but in 2 of 5 CI overlapped with zero.	Funnel plots, trim and fill	At best a rather small positive effect<br><br>The effect sizes found in the meta-analysis are small but significant.			Rejected as no effect size of HFO resection ratio on outcome. Difference in resection ratio quoted.

2.1.22 Ibrahim, Morgan (36) (2015)

**Meta-analysis**<br>***Publication year***<br>**Years of individual studies**	**# included Studies, Patients**<br>**Outcomes, Follow-up Durations**<br>**Model(s)**	**Feature**<br>**# of total patients with and without**<br>**(# of studies)**	**Population:**<br>**Lobe**<br>**Age**	**Effect Sizes (seizure freedom)**	**Rating the quality of the meta-analysis evidence using the GRADE guidelines [13]**								
					**Risk of Bias or Internal Validity[1]**	**Inconsistency of Results[2]**	**Indirectness of Evidence[3]**	**Imprecision[4]**	**Publication bias[5]**	**Large effect size?[6]**	**"Dose" response?[6]**	**All plausible residual confounding?[6]**	**Quality of the body of evidence (GRADE)**
Ibrahim, Morgan (36) (2015)<br>Same data as in [35]<br>++<br>2000 to 2011	20 articles<br>186 paediatric cases<br>Engel ordinal, median duration of follow up was 2.3yrs [1.3, 4.3]<br>Individual participant meta analysis:<br>Singular value decomposition and partial least squares method used on 11 features. Only 2 were significant.<br>partial least squares (PLS) to model multidimensional variance and study significant patterns in data that are associated with	Concordance EEG-MRI	Paediatric<br>tuberous sclerosis	+<br>Note no traditional effect sizes as used SVD and PLS (latent variable space)			Only for TS patients		undetected			+1<br>Permutation testing was performed\to evaluate the significance of the component, and bootstrapping was used to identify significant contributors to the component.<br>PLS accounted for latent structure of data and ordinal Engel outcomes classes	+++

	seizure outcomes												
		Focal ictal EEG		+									+++
		generalised seizure semiology		NS				-1<br><br>Fig 1 shows bootstrapping CI just about crosses zero, otherwise significant prognostic value					++
		focal interictal EEG		NS				-1<br><br>Skewed bootstrapping CI suggestive of possible positive effect					++
		gender		NS									+++
		tuber burden on MRI		NS									+++
		Epileptic spasms		NS									+++
		age at surgery		NS									+++
		lobe of resection		NS									+++
		age at onset		NS									+++
		Lesionectomy / multilobar resection surgery type		NS									+++

		Geographical location of surgery: N America vs elsewhere		NS									+++

2.1.23 Excluded Bonney, Glenn (41) (2015)

Data not fully extracted as explicitly state due to heterogeneity no formal meta-analysis was performed. Population was for gangliogliomas.

NB Fig 2 shows age at seizure onset to have inverse relationship with seizure freedom proportions, as well as mean or median duration of epilepsy having an inverse relationship with proportion seizure free.

2.1.24 Excluded West, Nolan (2) (2015)

Superseded by Cochrane review in 2019

2.1.25 Ruan, Yu (42) (2015)

Meta-analysis<br><br>*Publication year*<br><br>Years of individual studies	# included Studies, Patients<br><br>Outcomes, Follow-up Durations<br><br>Model(s)	Feature<br><br># of total patients with and without<br><br>(# of studies)	Population:<br><br>Lobe<br><br>Age	Effect Sizes (seizure freedom)	Rating the quality of the meta-analysis evidence using the GRADE guidelines [13]								
					Risk of Bias or Internal Validity[1]	Inconsistency of Results[2]	Indirectness of Evidence[3]	Imprecision[4]	Publication bias[5]	Large effect size?[6]	"Dose" response?[6]	All plausible residual confounding?[6]	Quality of the body of evidence (GRADE)
Ruan, Yu (42)<br><br>2015<br><br>1996 – 2014	10<br><br>594 patients<br><br>Engel I<br><br>At least 12 months<br><br>Fixed or random effects based on I^2	Extended excision of surrounding haemosiderin	Mainly Adults with cavernomas (but also few children)	234 of 316 in extended excision (74%)<br><br>Vs<br><br>189 of 278 in limited cavernoma resection (68%) were Engel I<br><br>OR 1.61 [1.10, 2.38]	-1 missing data in limitations<br><br>Note using their raw figures from 10 studies: OR 1.32 [uc] [0.94, 1.92]<br><br>More significant in males than females, in Europe and Asian studies than American studies, and more significant in cohort than case control studies. Also more significant in follow up durations less than 3 years and cavernomas greater than 2cm	I^2 = 28% p=0.16<br><br>Did perform sensitivity analyses to removing each of the 13 studies	Some studies used MRI others histology to determine if haemosiderin was resected		Egger's test normal				+

2.1.26 Cao, Liu (43) (2016)

**Meta-analysis** ***Publication year*** **Years of individual studies**	**# included Studies, Patients** **Outcomes, Follow-up Durations** **Model(s)**	**Feature** **# of total patients with and without** **(# of studies)**	**Population:** **Lobe** **Age**	**Effect Sizes (seizure freedom)**	**Rating the quality of the meta-analysis evidence using the GRADE guidelines [13]**								
					**Risk of Bias or Internal Validity[1]**	**Inconsistency of Results[2]**	**Indirectness of Evidence[3]**	**Imprecision[4]**	**Publication bias[5]**	**Large effect size?[6]**	**“Dose” response?[6]**	**All plausible residual confounding?[6]**	**Quality of the body of evidence (GRADE)**
Cao, Liu (43) (2016) 1995-2015	15 articles 380 sample size Engel I Min mean/median follow up time of 5 years Univariate then fixed or random effects	Seizure onset age ? (13)	Children with epilepsy undergoing hemispheric surgery	SMD = 0.26, [0.03, 0.49] P = 0.028		I square result was 40.2%, and the P value of the heterogeneity test was 0.116			Funnel and Egger				++
		Age at surgery ?(13)		NS[u]									++
		Seizure duration ?(5)		NS[u]									++
		Seizure type focal vs		Invert NS[u] OR[c*] 1.43 [0.58, 3.52]									++

		generalized 212(8)											
		Etiology		NS[u]									++
		epilepsia partialis continua 127 (7)		Invert NS[u] OR[c*] 0.46 [0.19, 1.15]]									++
		Surgical side ?(7)		NS[u]									++
		Gender male vs female<br><br>231 (10)		Invert NS[u] OR[c*] 1.15 [0.66, 2.01]									++
		MRI findings: abnormal vs normal ?(8)		OR 4.6 [1.27, 16.62]				-1 wide CI		Note unweighted OR is 1.49			+

2.1.27 Wang, Zhang (44) (2016)

**Meta-analysis** ***Publication year*** **Years of individual studies**	**# included Studies, Patients** **Outcomes, Follow-up Durations** **Model(s)**	**Feature** **# of total patients with and without** **(# of studies)**	**Population:** **Lobe** **Age**	**Effect Sizes (seizure freedom)**	**Rating the quality of the meta-analysis evidence using the GRADE guidelines [13]**								
					**Risk of Bias or Internal Validity[1]**	**Inconsistency of Results[2]**	**Indirectness of Evidence[3]**	**Imprecision[4]**	**Publication bias[5]**	**Large effect size?[6]**	**"Dose" response?[6]**	**All plausible residual confounding?[6]**	**Quality of the body of evidence (GRADE)**
Wang, Zhang (44) (2016)	18 studies 391 patients Mainly Engel I as per constituent studies in Table 1 random-effects model	Shorter epilepsy duration 128 (9)	(MRI neg TLE)	OR = 2.57 [1.21, 5.47]	NOS scores ranged from 4 to 6 stars Newcastle-Ottawa Scale	I^2 = 1%		-1 All have large CI and/or few patient numbers especially per study (imprecision/bias)	Forrest plots				+
		Ictal EEG localised to temporal lobe 125 (6)		OR = 3.89 [1.66, 9.08]	NOS scores ranged from 4 to 6 stars Newcastle-Ottawa Scale	I^2 = 0%							+
		Interictal EEG localised to temporal lobe 149 (7)		OR 3.38 [1.57, 7.25]	NOS scores ranged from 4 to 6 stars Newcastle-Ottawa Scale	I^2 = 0%							+
		PET scan results 127 (5)		NS p=0.06 OR = 2.11	NOS scores ranged from 4 to 6 stars Newcastle-	I^2 = 0%							+

				[0.95, 4.65]	Ottawa Scale								
		Gender: male vs female<br><br>146 (11)		NS<br><br>OR 1.44 [0.86, 2.41]<br><br>P=0.17	-1<br><br>2/11 studies had zero SF cases amongst males and 5 male and 5 females each or 2 males and 5 females – very few numbers with large CI<br><br>Collected data from even studies with very few cases<br><br>NOS scores ranged from 4 to 6 stars Newcastle-Ottawa Scale	I^2 = 0%, p=0.51							+
		Age at onset: children < 18 yrs vs >18yrs<br><br>69 (6)		NS<br><br>OR 0.68 [0.22, 2.08]	NOS scores ranged from 4 to 6 stars Newcastle-Ottawa Scale	I^2 = 0%, p=0.79							+

		Age at surgery <18 yrs vs >18yrs<br><br>78 (6)		NS<br><br>OR 1.09 [0.38, 3.07]	NOS scores ranged from 4 to 6 stars Newcastle-Ottawa Scale								+
		Side of surgery<br><br>320 (15)		NS<br><br>Slightly favours Left TL<br><br>OR 1.33 [0.84, 2.08]	NOS scores ranged from 4 to 6 stars Newcastle-Ottawa Scale	I^2 = 0%,							+
		Positive pathology		NS<br><br>(p=0.36)<br><br>OR=1.36 [0.7, 2.63]	NOS scores ranged from 4 to 6 stars Newcastle-Ottawa Scale	I^2 = 6%							+
		Mesial vs lateral TL epileptic focus<br><br>(as determined on sEEG, subdural grid; or ATL/SAH vs neocortectomy)<br><br>92 (8)		NS<br><br>Sightly favours mTL<br><br>OR 1.39 [0.61, 3.2]	NOS scores ranged from 4 to 6 stars Newcastle-Ottawa Scale								+

BMJ Publishing Group Limited (BMJ) disclaims all liability and responsibility arising from any reliance placed on this supplemental material which has been supplied by the author(s)

2.1.28 Giridharan, Horn (45) 2016

Giridharan, Horn (45)<br><br>2016<br><br>1986-2012<br><br>Baseline Quality of evidence: +++	17 studies, 2028 patients<br><br>Engel class I outcome >1 year post-operatively<br><br>Random-effects model, meta-regressions (logistic regression)	Without APOS within 30 days of surgery<br><br>Overall: 1983 (17)<br><br>Paediatric: 730 (6)	TLE and ETE<br><br>Mixed paediatric and adult. Subgroup analysis for overall APOS persistent in both.	Overall OR 4.2 [2.97, 5.93]<br><br>(Without APOS 73.5% seizure-free, vs with APOS 39%)<br><br>Paediatric subgroup OR 5.71 [3.32, 9.8]	-1 64.8% had presurgical lesion, not adjusted<br><br>Variable APOS definitions (7-30 days) but used meta-regression to explore this for under 24hrs only	Not detected<br><br>Subgroup analyses for paediatric, time of occurrence, semiology and meta-regressions to explore heterogeneity were performed			-1<br><br>Funnel plots and Egger's regression showed no bias. However, we note asymmetry in overall APOS, paediatric APOS and semiology group funnel plots in their Fig e1.	+1 large effect sizes for without APOS			++
		Earlier onset of APOS (within 24hrs)<br><br>222 (6)		NS 1.87 [0.89, 3.95]				-1 towards positive<br><br>Seizure-freedom more likely, but statistically not significant					+
		Postsurgical semiology different from presurgical<br><br>109 (3)		NS 4.24 [0.93, 19.25]				-1 towards positive<br><br>Seizure-freedom more likely, but statistically					+

								not significant					
		Subgroup meta-regression: mean age at surgery		NS									++
		Subgroup meta-regression: mean duration of epilepsy		NS									++
		Subgroup meta-regression: proportion with lesion		No data									No data, rejected

2.1.29 Hu, Zhang (46) 2016

Meta-analysis<br>*Publication year*<br>Years of individual studies	# included Studies, Patients<br>Outcomes, Follow-up Durations<br>Model(s)	Feature<br># of total patients with and without<br>(# of studies)	Population:<br>Lobe<br>Age	Effect Sizes (seizure freedom)	Rating the quality of the meta-analysis evidence using the GRADE guidelines [13]							
					Risk of Bias or Internal Validity[1]	Inconsistency of Results[2]	Indirectness of Evidence[3]	Imprecision[4]	Publication bias[5]	Large effect size?[6]	"Dose" response?[6]	All plausible residual confounding?[6]	Quality of the body of evidence (GRADE)
Hu, Zhang (46) 2016	56 studies<br>1528 pts<br>mean or median follow-up period ≥ 1 year<br>fixed and random effects	Developmental disorders<br>1041 (26)	Hemispheric	OR 0.61, 95% CI 0.46–0.82, p = 0.001		I^2 and Q statistics not significant heterogeneity detected			Funnel plots<br>undetected				+++
		Focal vs Generalized seizures<br>403 (15)		OR 1.84, [1.18, 2.89], p = 0.008									++
		Lateralized findings on interictal EEG<br>413 (7)		OR 1.66, [1.03, 2.67], p = 0.0									++

		Lateralized findings on ictal EEG<br>414 (7)		ictal: OR 1.88, [1.15, 3.07], p = 0.01									++
		contralateral MRI abnormalities<br>332 (6)		OR 0.46, [0.27, 0.77], p = 0.004									++
		Male vs female<br>575 (24)		NS OR 1.15, 95% CI 0.79–1.67, p = 0.46									++
		Side of resection<br>539 (29)		NS<br>OR 1.17, [0.79, 1.73], p = 0.43									++

2.1.30 Chen and Guo (47) (2016)

**Meta-analysis**<br><br>***Publication year***<br><br>**Years of individual studies**	**# included Studies, Patients**<br><br>**Outcomes, Follow-up Durations**<br><br>**Model(s)**	**Feature**<br><br>**# of total patients with and without**<br><br>**(# of studies)**	**Population:**<br><br>**Lobe**<br><br>**Age**	**Effect Sizes (seizure freedom)**	**Rating the quality of the meta-analysis evidence using the GRADE guidelines [13]**								
					**Risk of Bias or Internal Validity[1]**	**Inconsistency of Results[2]**	**Indirectness of Evidence[3]**	**Imprecision[4]**	**Publication bias[5]**	**Large effect size?[6]**	**"Dose" response?[6]**	**All plausible residual confounding?[6]**	**Quality of the body of evidence (GRADE)**
Chen and Guo (47) (2016)<br><br>1995-2015	11 studies<br><br>320 patients<br><br>275 patients undergoing epilepsy surgery<br><br>Engel I<br><br>Univariate then fixed effects and random effects also quoted in Fig 2	subtraction ictal and inter-ictal SPECT co-registered to MRI (SISCOM)	TL and ET	unweighted positive rate of SISCOM was 85.9% (275/320).					-1<br><br>Funnel plot was asymmetric and that there was publication bias				Rejected as unsuitable metrics, not an effect size, used non surgical data and unweighted (instead of Trim and Fill)
		concordant lateralized and localized to EZ	TL and ET	unweighted concordant rate of SISCOM was 65.3% (203/311)		$I^2 = 61.0\%$, $Q = 0.2938$, $p = 0.0042$	-1<br><br>71 pts used presumed EZ, 240 used actual resection		*-1*<br><br>*p* value of the Egger test was 0.0042				As above
	Fixed effects	Concordance SISCOM with EZ<br><br>275 (11)	TL and ET	OR 3.28 [1.90, 5.67]		$I^2 = 16.6\%$, p=0.285)			Egger's and Begg's test not significant				++

	Fixed effects	Concordance SISCOM with EZ<br><br>209 (11)	Subgroup ET	OR 2.44 [1.34, 4.43]		I^2 = 10.6%, Q = 10.06, p = 0.345			Egger's and Begg's test not significant				++

2.1.31 Excluded Ampie, Choy (48) (2016)

Meta-analysis<br>*Publication year*<br>Years of individual studies	# included Studies, Patients<br>Outcomes, Follow-up Durations<br>Model(s)	Feature<br># of total patients with and without<br>(# of studies)	Population:<br>Lobe<br>Age	Effect Sizes (seizure freedom)	Rating the quality of the meta-analysis evidence using the GRADE guidelines [13]								
					Risk of Bias or Internal Validity[1]	Inconsistency of Results[2]	Indirectness of Evidence[3]	Imprecision[4]	Publication bias[5]	Large effect size?[6]	"Dose" response?[6]	All plausible residual confounding?[6]	Quality of the body of evidence (GRADE)
Ampie, Choy (48)<br>2016<br>2005-2013	39 articles<br>88 patients of which 3 had only a biopsy with adjuvant radiotherapy (n=85)<br>"seizure-freedom" no definition, median of 24 months follow up<br>Fisher's exact	Gross total resection (vs subtotal resection)	Angiocentric gliomas<br>Majority of tumour locations was temporal lobe (39%)<br>2 – 79 years (average = 16 years)	36/37 GTR SF<br>Vs 9/16 STR SF<br>OR 28 [3.04, 258][c]	-2 included individual case reports<br>Did not clearly state what features were being compared for seizure freedom.<br>Did not define seizure freedom.			-2					Excluded as significant risk of bias, unweighted univariate statistics effect sizes overestimate true effect sizes<br>(exclusion criteria)

"Eight patients who presented with seizures (9%) reported seizure recurrence after surgical resection. GTR, when compared to STR, was associated with improved rates of seizure control (**p** = 0.0005). The remaining patients were seizure free post-operatively. In the 37 patients undergoing GTR, only one (2.7%) patient had seizure recurrence, occurring 15 months following surgery. Of the 16 patients undergoing STR, seven (44%) had recurrence of seizures in the post-operative period."

2.1.32 Harward, Chen (49) 2017

Harward, Chen (49)<br><br>2017<br><br>1990-2015<br><br>Baseline Quality: ++	27 series, 584 patients,<br><br>Engel class I outcome >1 year post-operatively<br><br>Mixed-effects model.	Age<18 yrs<br><br>9+21+13+9+7 +36+16 = 111 (7) (at surgery)	Occipital Lobe and posterior quadrant. Mixed adult and paediatric	OR 1.54 [1.13, 2.18]	Attempted to minimise selection bias: variables selected only if at least 80 patients across 5 studies<br><br>-1 No statistical adjustments “impossible to perform a multivariate analysis looking for interactions across variables” e.g. didn’t adjust for lesions	Salanova 1992 study seems to have an outlying large point effect for age, without attempts at subgroup explanation.		Note all 7 studies’ CI overlap OR of 1 but that of the overall effect does not	Undetected on funnel plots				+
		Focal pathological lesion<br><br>167 (9)		OR 2.08 [1.58, 2.89]									+
		Abnormal pre-operative MRI<br><br>132 (7)		OR 3.24 [2.03, 6.55]		Liava 2014 is the only study out of 7 without a CI overlapping OR of 1.							+

2.1.33 Krucoff, Chan (50) (2017)

Krucoff, Chan (50) (2017)<br>1989-2016<br>++	36 studies<br>782 patients<br>Engel I >12 months<br>Overall Engel I in 47% (n=369) of patients.<br>NB weights attributed to each study data are not shows on their forest plots.<br>Random-effects and pooled univariate	“Congruent” (=focal) electrophysiology (ictal/interictal or invasive EEG)<br>Table 2 & Fig 3: 192 (8)	patients who received repeat resective surgery for refractory focal epilepsy	OR = 3.6, [1.6, 8.2]									++
		Lesional (pathology: tumour, cyst, vascular malformation)<br>Table 2 & Fig 3:<br>507 (12)		OR = 3.2, [1.9, 5.3]									++
		surgical limitations over disease-related failure of first resection (incomplete resection) vs new emergent seizures or palliative cases<br>Table 2 & Fig 3:<br>273 (11)		OR = 2.6, [1.3, 5.3]	-1 heterogenous categorisations								+
		invasive monitoring<br>Table 2 & Fig 3:<br>210 (?)		OR = 0.4, [0.2, 0.9]									++

		trend whereby temporal were more likely to become seizure free than extratemporal resections<br><br>Table 2 & Fig 3:<br><br>943 (1st surgery = 447pts and 2nd resection = 496pts) (12)		NS OR=1.5 [0.8, 3.0]	No clear trend in 1st and 2nd resection subgroups, TL surgeries may have higher rate of redos?			Large CIs of individual studies					++
		Abnormal vs normal preop MRI (cf lesional pathology)<br><br>Table 2 & Fig 3:<br><br>196 (7)		NS OR 1.9 [0.6, 5.4]									++
		Non prognostic factors: [c*]<br><br>Gender<br><br>140 (?)<br><br>Epilepsy duration 60 (?)<br><br>Age at surgeries<br><br>1st surgery 194 (?)<br><br>2nd/last surgery 164 (?)<br><br>Time between resections 188 (?)		NS<br><br>Only univariate estimates can be calculated from the data in table 2<br><br>Gender male vs female NS OR[c*] 0.83 [0.42, 1.64]<br><br>Side of surgery	Seizure generalisations showed a non-significant trend towards worse outcomes			-1 imprecise and effect sizes and CIs estimated					+<br><br>no quantitative data on effect estimates provided, but calculated

		Laterality of resections 1st surgery 218 (?) 2nd/last surgery 209 (?)<br><br>Seizure generalization 145 (?)		operation #1 OR[c*] 0.73 [0.43, 1.25]<br><br>Operation #2 OR[c*] 0.77 [0.44, 1.33]<br><br>Focal vs generalised seizures:										
		Focal onset seizures with impaired awareness<br><br>vs aware or other seizures<br><br>206 (3)		Calculated from Table 2<br><br>Non weighted non-CMH univariate pooled [c*]<br><br>OR 1.61 [0.93, 2.8]<br><br>p=0.089	-1<br><br>no meta-analysis due to paucity of # of semiology studies									+<br><br>no quantitative data on effect estimates provided

2.1.34 * Excluded Nevitt, Staba (1) (2017)

**Meta-analysis** ***Publication year*** **Years of individual studies**	**# included Studies, Patients** **Outcomes, Model(s)**	**Feature** **# of total patients with and without** **(# of studies)**	**Population: Lobe** **Age**	**Effect Sizes (seizure freedom)**	**Rating the quality of the meta-analysis evidence using the GRADE guidelines [13]**								
					**Risk of Bias or Internal Validity[1]**	**Inconsistency of Results[2]**	**Indirectness of Evidence[3]**	**Imprecision[4]**	**Publication bias[5]**	**Large effect size?[6]**	**"Dose" response?[6]**	**All plausible residual confounding?[6]**	**Quality of the body of evidence (GRADE)**
Systematic Review<br>Nevitt, Staba (1) (2017)<br>+	2 studies<br>11 patients<br>Engel 1<br>12months follow up	ictal HFOs for epilepsy surgery decision making		NS<br>Found seizure free rates (Engel I) to be 55% - which isn't dissimilar to general seizure-freedom rates	Neither study compared surgical results guided by HFOs versus surgical results guided without HFOs.								+<br>Rejected as too few patients for formal meta-analysis and no effect sizes

This supersedes [51]

Rejected as 2 papers, 11 patients, no effect sizes.

*Found though other sources

2.1.35 *Excluded Genetic Stevelink, Sanders (7) (2018)

*Found though other sources

Stevelink, Sanders (7)<br><br>(2018)<br><br><2016<br><br>24 studies, 82 total patients, 38 patients with positive genetics,<br><br>15 different genetic aetiologias grouped into three categories<br><br>Raw proportions	5 studies<br><br>12 patients<br><br>Engel I<br><br>Last reported follow-up<br><br>Raw proportions	Germline Mutations in mTOR > synaptic or ion channel related mutations<br><br>38 genetics positive (24)	Genetic Epilepsies<br><br>Any Lobe	RR^c = (7/12) / (2/14) = 4.08 [1.04, 16.06]	-2<br><br>8 of 12 germline mTOR mutations were lesional		-1<br><br>15 different genetic aetiologias grouped into three categories	-1<br><br>Very small numbers and large CI					Excluded as few numbers and all lesional without adjustments; and no RR calculations
	6 studies<br><br>18 patients<br><br>Last follow up	Somatic or Mosaic mTOR mutations >synaptic/ion ch mutations	Genetic Epilepsies<br><br>Any Lobe	RR^c = 15/18 /<br><br>2/14 = 5.833 [1.59, 21.40]	-2<br><br>All 18 mosaic mTOR were lesional								Excluded as few numbers and all lesional and no RR calculations
	2 studies<br><br>12 patients<br><br>Last follow up	Other: microdeletions	Majority lesional	Sz free = 10/12 = 75%									Excluded as few numbers and all lesional and no RR calculations
	3 studies<br><br>21 patients	Other: NF1	HS or low-grade tumours	12/21 = 57%	-2 majority were lesional								Excluded as few numbers and all lesional and no RR calculations

2.1.36 Excluded Pilipović-Dragović, Ristić (9) (2018)

Pilipović-Dragović, Ristić (9) (2018)<br><br>1993-2017	7 studies<br><br>253 patients<br><br>Engel I<br><br>univariate meta-regression	Localised interictal EEG	Parietal Lobe	No effect sizes given besides a univariate “b”. e^b would give the OR, unadjusted<br><br>e^b=4.80=OR	-2 univariate meta regression without inverse variance weighting for features.<br><br>No adjustments.	-1<br><br>Significant heterogeneity in the meta-analysis (Q p<0.001, I^2=80%	Poor quality, CI of porportions exceed 1 in “Engel Forrest Plot” Figure 1	Excluded as univariate meta regression without adjusting for other factors, “b” coefficient of logistic regression quoted. Although we could calculate the effect size and estimate the CI, their figure 1 showed CI exceeding proportion of 1 for seizure freedom, so we didn’t feel we could reliably deduce effect sizes from the rest of the paper. Even then, the results are generally consistent with included meta-analyses. The interesting ones were presence of aura and somatosensory aura not being significant as prognostic features which we didn’t find in other meta-analyses. Imaging lesion and localised ictal EEG were even NS in their study , again no effect sizes.					
		Tumour pathology		e^b=1.4=OR									Excluded as above
		Age at onset, duration of epilepsy, age at surgery, mean follow up, imaging done, presence of aura, somatosensory aura, GTCS, imaging lesion, localized ictal EEG, invasive study, MCD, right sided surgery		NS univariate meta-regression	NB: invasive EEG, ictal EEG localization and GTCS are shown in other meta-analyses to be prognostic								Excluded As bove

2.1.37 Jain, Tomlinson (52) 2018

Meta-analysis<br>*Publication year*<br>Years of individual studies	# included Studies, Patients<br>Outcomes, Follow-up Durations<br>Model(s)	Feature<br># of total patients with and without<br>(# of studies)	Population:<br>Lobe<br>Age	Effect Sizes (seizure freedom)	Rating the quality of the meta-analysis evidence using the GRADE guidelines [13]								
					Risk of Bias or Internal Validity[1]	Inconsistency of Results[2]	Indirectness of Evidence[3]	Imprecision[4]	Publication bias[5]	Large effect size?[6]	"Dose" response?[6]	All plausible residual confounding?[6]	Quality of the body of evidence (GRADE)
Jain, Tomlinson (52) 2018<br>Used GRADE	19 compared ATL vs SAH<br>?pts<br>Engel Ia or ILAE 1 or Engel 1, 12 months follow up<br>Bayesian random effects NMA	ATL vs SAH (mix of transcortical, transsylvian and subtemporal approaches)	Mainly adults	NS<br>OR 1.14, 95% CI 0.93 to 1.39; p=0.201<br>NS OR 1.15, 95% credible interval (CrI) 0.84-1.15									++<br>[52] 2018

2.1.38 Shan, Fan (53) (2018)

**Meta-analysis**<br>***Publication year***<br>**Years of individual studies**	**# included Studies, Patients**<br>**Outcomes, Follow-up Durations**<br>**Model(s)**	**Feature**<br>**# of total patients with and without**<br>**(# of studies)**	**Population:**<br>**Lobe**<br>**Age**	**Effect Sizes (seizure freedom)**	**Rating the quality of the meta-analysis evidence using the GRADE guidelines [13]**								
					**Risk of Bias or Internal Validity[1]**	**Inconsistency of Results[2]**	**Indirectness of Evidence[3]**	**Imprecision[4]**	**Publication bias[5]**	**Large effect size?[6]**	**"Dose" response?[6]**	**All plausible residual confounding?[6]**	**Quality of the body of evidence (GRADE)**
Shan, Fan (53) 2018<br>**1965-2016**	23 studies<br>2641 patients<br>Engel at last point of follow up 1mo to 17 yrs<br>Fixed-effects	Age >= 45 yrs<br>++ (at surgery)<br>1065 (6)	Supratentorial low grade gliomas in adults	RR 1.12 [1.01, 1.23]	NOS scale for individual studies<br>GRADE evidence scale used		-1 Engel I at 1 month post-surgery also included		undetected				+
		Focal seizures<br>+++<br>796 (3)		RR 0.76 [0.67, 0.85]	Looked at generalized seizures and focal separately which was seems redundant	Note the caption on Fig 3 is incorrect and confusing.							+
		Prolonged history of seizures >1 yrs		RR 0.82 [0.75, 0.91]									+

		++ <2641 (<23)											
		Gross total resection cf subtotal resesction ++ 1379 (16)		RR 1.47 [1.37, 1.59]									+
		Tumor location TL vs ET ++ <2641 (<23)		NS NA									+
		Sex ++ <2641 (<23)		NS NA									+
		Tumor histology astro vs non astro ++ <2641 (<23)		NS NA									+
		Imaging characteristics (enhancement, oedema, mass effect) No GRADE score provided <2641 (<23)		NS NA									+

2.1.39 Shang-Guan, Wu (54) (2018)

**Meta-analysis**<br>***Publication year***<br>**Years of individual studies**	**# included Studies, Patients**<br>**Outcomes, Follow-up Durations**<br>**Model(s)**	**Feature**<br>**# of total patients with and without**<br>**(# of studies)**	**Population:**<br>**Lobe**<br>**Age**	**Effect Sizes (seizure freedom)**	**Rating the quality of the meta-analysis evidence using the GRADE guidelines [13]**								
					**Risk of Bias or Internal Validity[1]**	**Inconsistency of Results[2]**	**Indirectness of Evidence[3]**	**Imprecision[4]**	**Publication bias[5]**	**Large effect size?[6]**	**"Dose" response?[6]**	**All plausible residual confounding?[6]**	**Quality of the body of evidence (GRADE)**
Shang-Guan, Wu (54) (2018)<br>1967-2017	7 studies<br>245 patients<br>Follow up >6 months<br>Fixed/random effects	extended lesionectomy with lesionectomy (:=resection of lesion and surrounding hemosiderin is sufficient)<br>245 (7)	any	NS<br>OR 1.30 [0.66, 2.56]<br>Removed 1 article with selection bias:<br>OR 0.96 [0.44, 2.08]	NOS>4 only included, English, case reports excluded<br>Sensitivity analysis		-1<br>f/up 6 months		Eggers and Beggs				+
		Average age <18 yrs excluded as only 1 trial		NS see table 2									Only 1 study
		Average >18yrs vs other at surgery		NS OR 0.95 [0.38, 2.37]									+

		Year of publication <2010 vs >2010		NS OR see table 2									+
		NOS > 6		NS OR 0.51 [0.21, 1.26] see table 2									+
		Seizure duration in years		NS OR see table 2									+
		Follow up in months		NS OR see table 2									+

2.1.40 Kobulashvili, Kuchukhidze (55) (2018)

**Meta-analysis**<br><br>***Publication year***<br><br>**Years of individual studies**	**# included Studies, Patients**<br><br>**Outcomes, Follow-up Durations**<br><br>**Model(s)**	**Feature**<br><br>**# of total patients with and without**<br><br>**(# of studies)**	**Population:**<br><br>**Lobe**<br><br>**Age**	**Effect Sizes (seizure freedom)**	**Rating the quality of the meta-analysis evidence using the GRADE guidelines [13]**								
					**Risk of Bias or Internal Validity[1]**	**Inconsistency of Results[2]**	**Indirectness of Evidence[3]**	**Imprecision[4]**	**Publication bias[5]**	**Large effect size?[6]**	**"Dose" response?[6]**	**All plausible residual confounding?[6]**	**Quality of the body of evidence (GRADE)**
55<br><br>2018<br><br>1990 – 2015	48 of 94 eligible for meta-analysis (40 for subgroup analysis)<br><br>534 patients subgroup analysis<br><br>Completely seizure free (IA, 1)<br><br>At least 12 months<br><br>univariate random-effects meta-analytical models	Long-term monitoring (LTM / VT) localising vs non-localising in subsequent seizure outcome<br><br>(sensitivity total studies = 44)<br><br>(specificity total studies = 34)	All<br><br>Lesional TLE n=406 (33)	Sensitivity 0.7 [0.6, 0.8]<br><br>Sensitivity higher in tumours for outcomes<br><br>Specificity 0.4 [0.27, 0.54]<br><br>Sensitivity 0.85 [0.81, 0.89]<br><br>highest in MCD/FCD ~0.95 [~0.71, 1]<br><br>Spec 0.19 [0.13, 0.28]<br><br>Specificity high for gliosis in	Also looked at covariates: lesional on MRI, TLE vs ETE, pathology including HS, length of follow-up, invasive and non-invasive LTM in their supplementary tables	**Sensitivity:**<br><br>-1 Very large differences<br><br>I^2 = 94.9%<br><br>P<0.0001<br><br>**Specificity:**<br><br>-1 Very large heterogeneity<br><br>I^2 = 92.6%<br><br>P<0.0001					With certain sensitivities yes, such as increased specificity with ETE and increased sensitivity with proportion of concordant LTM/MRI	+2 investigated covariates and sensitivity analyses including TLE, ETE, and abnormal MRI etc	+<br><br>Very Low

			Nonlesional TLE n=14 (3)<br><br>Lesional ETE n=108 (16)<br><br>Nonlesional ETE n=6 (2)	lesional TLE 0.41 [0.18, 0.69]<br><br>Sens 0.47 [0.36, 0.58]<br><br>Spec 0.35 [0.21, 0.53]									
		Long-term monitoring (LTM / VT)<br><br>Odds ratios representing the odds of being seizure-free if the LTM is localizing and concordant with the surgical resection compared to non localizing LTM	Lesional TLE<br><br>Lesional ETE<br><br>Nonlesional TLE<br><br>Nonlesional ETE	OR 1.41 [0.79, 2.53]<br><br>OR 0.46 [0.2, 1.07]<br><br>OR 0.6 [0.01, 35.86]<br><br>1 [0.06, 17.51]			-1 indirect evidence as their chose of definitions for LTM consists of focal and prognosis (see their discussion)	-1 imprecision for Nonlesional cases as few cases			See above	See above	+<br><br>Very Low

2.1.41 Harris, Phillips (56) 2019

Harris, Phillips (56) 2019<br>1992-2016<br>Baseline Quality: ++	19 articles, 187 children<br>Engel I outcome at the longest reported follow-up time.<br>Individual participant univariate and multivariate Cox regression analysis.	Younger age at onset	Rasmussen's<br>Paediatric	HR 0.91^{u} [0.85, 0.96]<br>NS HR 0.95^{m} [0.87, 1.04]	HR 1.10^{u} [1.04, 1.18]<br>NS HR 1.05^{m} [0.96, 1.15]		-1 Adjusted for the variable length of follow-up, but not lesional or other known factors<br>The NS features were not adjusted for known features in multivariate analyses<br>-1 Reporting bias: 7 out of 19 studies had ≤ 5 patients	Probably not significant			-1 suspected but "unable to measure between-study heterogeneity and publication bias due to the very limited sample size per study"			Note not significant on adjustment for follow up	+
		Younger age at surgery		HR 0.93^{u} [0.89, 0.97]<br>NS^{m} HR 0.95^{m} [0.90, 1.0]	HR 1.08^{u} [1.03, 1.12]<br>NS^{m} HR 1.05^{m} [1.0, 1.11]										+
		Shorter duration of epilepsy		HR 0.92 [0.88, 0.97]	HR 1.09 [1.03, 1.14]										+
		Hemispherectomy (vs resective)		HR 0.28^{u} [0.18, 0.45]<br>HR 0.30^{m} [0.18, 0.49]	HR 3.57^{u} [2.22, 5.56]<br>HR 3.33^{m} [2.04, 5.56]									Remained significant on multivariate analysis but it seems only adjusted for length of follow up	+
		General seizure semiology		NS HR 0.8 [0.43, 1.51]	NS HR 1.25 [0.66, 2.33]										+

		Mod-severe developmental delay		NS HR 0.64 [0.31, 1.32]	NS HR 1.56 [0.76, 3.23]										+

2.1.42 Bjellvi, Olsson (57) (2019)

Bjellvi, Olsson (57) (2019)<br><br>Baseline Certainty: ++<br><br>Except for <10 yrs vs >10 yrs +++ as over 1000	12 studies<br><br>1,545 patients<br><br>Engel I<br><br>>12months (although some exceptions were made)<br><br>Random-Effects	Shorter duration of epilepsy at different points	Children and adults, all lobes	**RR 1.2-1.33**<br><br>**(Risk difference 0.15 to 0.21)**	"Moderate risk of bias"<br><br>"did not justify downgrading the evidence level"	**-1**<br><br>3/12 studies reported no association between duration and outcome<br><br>1/12 study was in favour of longer duration	"some concerns"<br><br>"did not justify downgrading the evidence level"		Studies that only reported<br><br>mean or median duration of epilepsy for patients grouped<br><br>by seizure outcome were not included.		Evaluated<br><br><5 yrs vs >10yrs<br><br>To investigate if a larger time gap in epilepsy duration resulted in a larger effect – not present		++<br><br>GRADE score provided by study
		<2 vs >2yrs<br><br>388 (3)		RR 1.20 [1.05, 1.39]		I^2 = 9%							
		<5 vs >5yrs<br><br>551 (4)		RR 1.24 [1.08, 1.42]		I^2 = 55%							
		<10 vs >10<br><br>1376 (10)+++		1.25 [1.09, 1.43]		I^2 = 66%<br><br>(p=0.002)							
		<20 vs <20<br><br>346 (3)		1.33 [1.08; 1.65]		I^2 = 20%							
		<5 vs >10		1.32 [1.19; 1.46]		I^2 = 0%							

		430 (4)											

2.1.43 *West, Nevitt (3)* 2019

West, Nevitt (3) 2019<br><br>1984-2013<br><br>Baseline Quality: +++<br><br>182 studies (9 RCTs, 29 multivariate studies) 16855 participants<br><br>Engel I or Ia, variably between 1 year and 5 years follow-up.<br><br>Fixed and Mixed-Effects<br><br>Note this study already includes GRADE scores per features and this is used as the starting	43 studies<br><br>3999 patients<br><br>Combined good outcomes, various follow up Fixed effects	abnormal pre-operative MRI<br><br>3999 (43)		RR 1.28 [1.20, 1.37]	-1 †	I^2 = 38.57% p=0.01, no difference between subgroups by outcome							++<br><br>Unclear why they didn't use Random effects here
	21 studies<br><br>1547 patients<br><br>Combined good outcomes, various follow up<br><br>Fixed effects	invasive monitoring<br><br>1547 (21)		RR 0.85 [0.78, 0.93]	-1 †	I^2 = 37.28% p=0.04;, no difference between subgroups by outcome							++<br><br>Unclear why they didn't use Random effects here
	1 study<br><br>70 pts<br><br>GRADE: ++<br><br>Engel Ia at 1 yr<br><br>Fixed effects	Complete / total vs partial resection<br><br>70 (1)	Adults >18yrs	RR 1.82<br><br>[1.12, 2.93]	-1<br><br>Insufficient information regarding methods of randomization and allocation concealment in the study†								+ Only 1 study

point individually despite overall the study being rated as +++ by us.	46 studies<br><br>4430 patients<br><br>Combined good outcomes, various follow up<br><br>Fixed	mesial temporal sclerosis<br><br>4430 (46)	TLE	RR 1.17 [1.12, 1.23]	-1 †	I^2 = 27.79% p=0.04; no difference between subgroups by outcome (I^2 63% p 0.06) and certainly not by effect estimates							++<br><br>Unclear why they didn't use Random effects here
	23 studies<br><br>1778 patients<br><br>Combined good outcomes, various follow up<br><br>Fixed	MRI and EEG concordance<br><br>1778 (23)		RR 1.25 [1.15, 1.37]	-1 †	I^2 = 26%, no difference between subgroups by outcome							++
	15<br><br>1368<br><br>Combined good outcomes, various follow up<br><br>Fixed	Febrile seizures<br><br>1368 (15)		RR 1.09 [1.01, 1.17]	-1 †	-1<br><br>I^2 = 32%, p=0.11; >1 yr SF subgroup had good outcomes with febrile seizures but the other subgroups did not (subgroup I^2 49%, p 0.14)							+
	46<br><br>3572<br><br>Combined good outcomes, various follow up	presence of FCD<br><br>3572 (46)		RR 0.90 [0.85, 0.95]	-1 †	I^2 28%, no significant different between outcome subgroups (I^2 0%, p 0.83)							++

	Fixed												
	41<br>3357<br>Combined good outcomes, various follow up<br>Random-Effects	presence of tumour<br>3357 (41)		RR 1.23 [1.14, 1.32]	-1 †	Mixed effects used as I^2 40.59% p=0; no significant outcome subgroup differences							++
	37<br>2976	right-sided resection<br>2976 (37)		NS<br>RR 0.96 [0.91, 1.01]	-1 †	I^2 28.22% p0.06 for total of 47 studies. I^2 0% p0.6 for subgroup heterogeneity. I^2 30% p=0.05 for total of the TLE vs ET subgroup analyses with TLE vs ET subgroups themselves not significant. Unclear why TLE/ET subgroup has fewer studies.		-					++
	Pg. 35<br>Analysis 4.11<br>18 studies<br>1414 pts	unilateral interictal spikes vs bilateral<br>1414 (18)	Mixed	Pooled RR 1.14<br>[1.05, 1.24]	CI adjusted for outcome scale<br>Definition was likely to have influenced the analysis, e.g. non-lateralising vs contralateral	-1 I^2 = 67% overall and mixed effects using only Engel outcomes is non-significant: RR [0.88, 2]. Subgroup		-1 small studies with imprecise results. Mixed-effects model RR has no statistical significance.			In best case scenario of pooled effect rather than mixed-effects, the point estimation		+

	Various outcome scales<br><br>Fixed effects				spikes, focal vs non-focal spikes †	analyses (TLE vs ET) do not explain these differences.					is 14% better. E.g. if bilateral 60% SF, unilateral spokes, 70% SF. This variable only explains one-third of the missing outcome variance, with NNT around 10.		
	40<br><br>3013<br><br>Various outcome scales<br><br>Fixed effects	Complete resection (extent of resection) vs incomplete<br><br>2930 (39)<br><br>Temporal 1266 (13)<br><br>ET 30 (1)<br><br>Mixed TLE and ET 1634 (25)		RR 1.41 [1.32, 1.50]<br><br>TL subgroup RR 1.11 [1.03, 1.2]<br><br>ET RR 2.0 [0.76, 5.29]	-1 †	-1<br><br>I^2 77.76% with p <0.0001; outcome subgroup differences also significant I^2 89.51% p<0.0001although the direction of effects are similar.<br><br>Extratemporal subgroup ommited as only 1 study.							+<br><br>Unclear why random effects method was not used<br><br>ET subgroup Only 1 study
	1 study<br><br>47 pts<br><br>GRADE: ++ randomised	Subtemporal vs transsylvian approach to SAH 47 (1)	TLE<br><br>Adults>18 yrs	NS RR 0.92<br><br>[0.59, 1.46]	Participants not completing one year of follow-up measures were excluded from the study†								+ Only 1 study

	ILAE 1 at 1 year													
	1 study<br>40 patients<br>GRADE: ++ randomised<br>Engel 1 and IA at 1 and 5 years<br>Fixed effects	ATL vs parahippocampectomy PHC Engel IA 5yrs<br>28(1)	TLE<br>Adults >18yrs	NS<br>RR 0.57 [0.21, 1.52]	Outcome assessors were not blinded†		Excluded children from the study	-1<br>Despite the CI overlap, point estimates favour ATL, whether at 1 or 5 yrs or Engel I or IA.						+ Only 1 study
		SAH vs PHC Engel IA at 5 yrs<br>29 (1)	TLE<br>Adults >18yrs	NS<br>RR 0.54 [0.21, 1.39]	Outcome assessors were not blinded†			-1<br>Despite the CI overlap, point estimates favour SAH, whether at 1 or 5 yrs or Engel I or IA.						+ Only 1 study
		ATL vs SAH<br>29 (1)	TLE<br>Adults >18yrs	NS<br>RR 1.07 [0.53, 2.16]	Outcome assessors were not blinded†									+ Only 1 study
	1 study<br>207 pts<br>GRADE: +++	2.5cm vs 3.5cm ATL resection 207 (4)	TLE<br>Adults >18yrs	NS<br>RR 1.02	†									++

	randomised<br><br>Engel 1 at 1 yr			[0.86, 1.2]									
	1 study<br><br>70 patients<br><br>GRADE: randomised<br><br>ILAE 1 at 1yr<br><br>Fixed effects	Total hippocampectomy > partial 70 (1)	TLE	RR 1.82 [1.12, 2.93]	†								++<br><br>Couldn't find a Cochrane GRADE, so rated the study as 3+ as it was randomised. Only 1 study
	1 study<br><br>58 patients<br><br>GRADE: ++<br><br>Engel Ib at least, between 25-36months<br><br>Fixed effects	ATL>stereotactic radiosurgery 58 (1)	TLE<br><br>Adults >18yrs	RR 1.52<br><br>[1.01, 2.22]	Insufficient information regarding methods of randomization and allocation concealment in the study†			Note the limit approaches 1 and not fully adjusted, so could have confounders					+<br><br>Only 1 study
	1 study<br><br>43 patients<br><br>GRADE: ++ randomised	Resection ± corpus callosotomy vs resection alone in LGS 43 (1)	LGS<br><br>Children < 18 yrs	NS for all three<br><br>5yrs:<br><br>RR 1.09<br><br>[0.53, 2.21]	Outcomes split into three follow-up groups of 1,3 and 5 years<br><br>Inadequate method of quasi-randomisation. Unclear if blinded.†								+

	Engel 1 at 1, 3 and 5 years<br><br>Fixed effects												
	1 study<br><br>60 patients<br><br>GRADE: +++ randomised<br><br>Engel 1 at 2 years<br><br>Fixed effects	ATL + CCT vs ATL alone.<br><br>60 (1)	TLE<br><br>Children and Adults	NS<br><br>RR 1.22<br><br>[0.85, 1.76]	Inadequate method of quasi-randomisation. Unclear if blinded.†								++
	7 studies<br><br>551 patients<br><br>Combined good outomces, various follow up<br><br>Fixed	History of Head injury<br><br>551 (7)		NS<br><br>RR 0.99 [0.86, 1.13]	-1 †	-1<br><br>I^2 46%, p 0.08, subgroup analyses by outcome were very different and inconsistent							+
	5<br><br>317<br><br>Combined good outomces, various follow up<br><br>Fixed	Encephalomalacia<br><br>317 (5)		NS<br><br>RR 0.78 [0.52, 1.17]	-1 †	No significant difference between outcome subgroups							+

	6<br>542<br>Combined good outomces, various follow up<br>Random-effects and Fixed for temporal subgroup	postoperative discharges<br>542 (6)		NS<br>Adjusted for outcomes RR 0.91 [0.68, 1.22]<br>For TLE subgroup RR 0.81 [0.70, 0.94]	-1 †	Different total results for subgroups TLE/ET vs outcome subgroups, but both NS.							++
	Analysis 4.10<br>19 studies<br>1488 pts	vascular malformations<br>1488 (19)		NS pooled RR 1.07 [0.94, 1.21] adj for outcomes scale	- 1 †	No broad changes to result according to outcomes scales (Engel, Other or seizure freedom for 1 yr).	I^2= 0% for both overall heterogeneity and subgroup differences						++

2.1.44 Chen, Chen (58) (2019)

Meta-analysis *Publication year* Years of individual studies	# included Studies, Patients Outcomes, Model(s)	Feature # of total patients with and without (# of studies)	Population: Lobe Age	Effect Sizes (seizure freedom)	Rating the quality of the meta-analysis evidence using the GRADE guidelines [13]								
					Risk of Bias or Internal Validity[1]	Inconsistency of Results[2]	Indirectness of Evidence[3]	Imprecision[4]	Publication bias[5]	Large effect size?[6]	"Dose" response?[6]	All plausible residual confounding?[6]	Quality of the body of evidence (GRADE)
Chen, Chen (58) (2019)<br><br>1991-2018<br><br>+++	48 studies<br><br>1580<br><br>Engel I and II<br><br>Combinations of fixed-effects, random-effects and network-analyses (NMA).	FCD type I vs type II<br><br>1580 +++<br><br>(34)	Patients with focal dysplasia<br><br>Any lobe<br><br>Children and adults	OR 0.52<br><br>[95%CI<br><br>0.41, 0.65]	Trial sequence analysis, sensitivity analyses including removing individual studies<br><br>Subgroup analyses for geographical locations.<br><br>Palmini system of FCD (no FCD type III which was developed by IALE in 2011)	I^2=14%, p=0.24<br><br>Not in subgroup analyses in asia (OR=1.24 []0.75, 2.04]	-1<br><br>Engel II was considered seizure free		Begg rank correlation test and Egger linear regression test with trim and fill as necessary				++
		FCD and **incomplete resection**<br><br>567 ++<br><br>(16)		OR 0.08<br><br>[95%CI<br><br>0.05, 0.14]		I^2=0%, p=0.68				+2 large OR<0.1			+++

		FCD and **extratemporal location** 370 (12)		0.52 (95%CI 0.29, 0.94]		I^2=0%, p=0.8							+
		FCD IIb in network meta-analyses of FCD subtypes		OR 1.89 [1.01, 3.57]		P=0.048		-1 CI of OR approached 1					+

2.1.45 Toth, Papp (59) 2019

Toth, Papp (59)<br><br>2019<br><br>1996-2017	31 studies<br><br>1999 patients<br><br>Engel I, >6 months<br><br>Random-effects	sEEG > subdural<br><br>overall: 1999 (31)<br><br>Nonlesional: 237 (15)<br><br>Lesional: 665 (21)<br><br>TL: 470 (17)<br><br>ET: 420 (14)	TL and ET<br><br>Adults and Children (but not data from children only)	Overall RR = 64.7% [59.2, 69.8] / 55.9% [50.9, 60.8]<br><br>NS Nonlesional NS RR = 52% [37.3, 66.3] / 54.4% [40.6, 67.6]<br><br>Lesional RR = 71.6% [61.6, 79.9] / 57.3% [48.7, 65.6]<br><br>TL RR = 73.9% [64.4, 81.6] / 56.7% [51.5, 61.9]<br><br>ET RR = (61% [51, 70.2]) / (46.7%[36.5, 57.2]) = 1.31	-1 average follow up for SEEG was 10 months while for SDG it was nearly 19months.<br><br>Significant differences overall (p = 0.02), lesional (p = 0.031), and also, temporal sugroups (p = 0.002)	Overall SEEG: I2 = 11.86%;p = 0.318<br><br>subdural grid: I2 = 54.47%;p = 0.002	-1<br><br>studies <6 months follow-up durations; we are interested in at least 12 months		Funnel plots, Egger's tests: no overall changes or subgroup changes			[Unlcear if ET was significant or not as it mentions lesional in the text whereas it should mention ET]	+

2.1.46 Excluded Pellino, Gencarelli (8) (2020)

**Meta-analysis**<br>***Publication year***<br>**Years of individual studies**	**# included Studies, Patients**<br>**Outcomes, Model(s)**	**Feature**<br>**# of total patients with and without**<br>**(# of studies)**	**Population:**<br>**Lobe**<br>**Age**	**Effect Sizes (seizure freedom)**	**Rating the quality of the meta-analysis evidence using the GRADE guidelines** [13]								
					**Risk of Bias or Internal Validity**[1]	**Inconsistency of Results**[2]	**Indirectness of Evidence**[3]	**Imprecision**[4]	**Publication bias**[5]	**Large effect size?**[6]	**"Dose" response?**[6]	**All plausible residual confounding?**[6]	**Quality of the body of evidence (GRADE)**
Pellino, Gencarelli (8) (2020)	21 articles<br>24 patients<br>univariates	Distribution of neurocutaneous melanosis (focal)	Children with parenchymal Neurocutaneous melanosis	Bilateral or isolated amygdala involvement carried the best rates of seizure-freedom 100% cf multiple localisations 58% (>6 months, 24 paediatric cases amongst 21 studies).	Few patients, averaging 1 patient per study								Excluded as no summary effect size, not a meta-analysis and few numbers

2.1.47 Widjaja, Jain (60) 2020

Widjaja, Jain (60) 2020<br><br>1990-2017<br><br>Baseline Quality: +++	258 studies, 4891 patients<br><br>"seizure-freedom" ≥12months follow-up, Random-effects, meta-regression and network meta-analysis	Lesional epilepsy (=abnormal MRI)<br><br>883 (10)	No mention of distribution by lobe<br><br>Paediatric	OR 1.85<br><br>[1.14, 2.94]	Sensitivity analysis performed by removing the single RCT from results.<br><br>Newcastle-Ottawa Quality Assessment Scale for observational studies.	2 of 10 studies had a reverse OR point estimate, one of which was a small study, $I^2<50\%$, p=0.01.	-1<br><br>SF not clearly defined, some individual studies included Engel Classes I and II		Funnel plots and trim and fill test to impute bias effect estimates: difference between observed and imputed <10%<br><br>NB the MR for study quality score showed for subgroups of tumour and ETE, higher quality was associated with reduced SF percentages - although not statistically significant, the magnitude of effect was significant<br><br>(-0.31 and<br><br>-0.16 respectively)			Age at surgery and age at seizure onset associated with seizure freedom in general and especially for TLE and ETE (age at surgery only) but not for hemisphere-ctomies, tumors or MCD	+
		Pathologies<br><br>Tumour, HS > Rasmussen > MCD, TS > HH		Proportions only									+
		Complete resections<br><br>893 (15)		OR 7.69<br><br>[4.76, 12.5]		consistent				+1			++
		Age at seizure onset in general (MR) ? (<30)		SF % coefficient +0.346 [0.21, 0.49]							+1 meta-regression		++
		Age at surgery in general (MR) ? (<30)		SF % -0.19 [-0.27, 0.12]							+1 meta-regression		++
		Surgery Locations hemispheric (NMA) ? (~23)		Vs medical OR 13.1 [4.3, 41]		NMA Surgical locations cf with medical therapy, pairwise comparisons				Large OR only vs medical			+

				But NS for vs ET		similar except for TLE vs ETE							
		Surgery Locations Temporal Lobe (NMA) ? (~23)		OR 9.3 [3.3, 27] vs medical<br><br>Also significant for vs ET:<br><br>OR 2 [1.4, 2.9] direct and NMA p=0.025						Large OR only vs medical			+
		Surgery Locations Extratemporal Lobe (NMA) ? (~23)		OR 4.7 [1.7, 14] vs medical<br><br>But NS except for worse cf TL as above						Large OR only vs medical			+

2.1.48 Excluded Brændholt and Jensen (61) (2020)

6 original studies, 59 patients.

iMSI can reliably localize the EZ in focal epilepsy, but not clearly predictive of outcomes. Sensitivity and specificity are provided for EZ predictions in seizure free and non-seizure free groups, and compared with that of icEEG. The latter which is usually either non-prognostic or has a negative prognostic value. The effect sizes were for EZ prediction rather than outcomes per se.

2.1.49 Lamberink, Otte (62)(2020)

Meta-analysis<br>*Publication year*<br>Years of individual studies	# included Studies, Patients<br>Outcomes, Follow-up Durations<br>Model(s)	Feature<br># of total patients with and without<br>(# of studies)	Population:<br>Lobe<br>Age	Effect Sizes (seizure freedom)	Rating the quality of the meta-analysis evidence using the GRADE guidelines [13]								
					Risk of Bias or Internal Validity[1]	Inconsistency of Results[2]	Indirectness of Evidence[3]	Imprecision[4]	Publication bias[5]	Large effect size?[6]	"Dose" response?[6]	All plausible residual confounding?[6]	Quality of the body of evidence (GRADE)
Lamberink, Otte (62)<br>2020<br>Jan 1, 2000, and Dec 31, 2012	37 collaborating tertiary referral centres across 18 European countries<br>Proportions of individuals who were Engel I were reported for 11 categories of histopathological diagnosis at 1, 2 and 5 years of follow-up.<br>8191 patients for 2 years of follow up data used as ORs available for this and much less missing values than for 5 years follow up. 9147 total patients in study.	low-grade epilepsy associated neuroepithelial tumour (LEAT)<br>1325 (<37)<br>(similar for LGNET, DNET, ganglioglioma, and others: angiocentric neuroepithelial tumour, pilocytic astrocytoma, gangliocytoma, papillary glio-neuronal tumour, and other low-grade neuroepithelial tumours) – majority were gangliogliomas and DNET<br>Ganglioglioma	Children and Adults	77·5%<br>80·4%	89·5% of patients had outcomes followed up for 2 years (8191 pts)<br>Missing data were imputed (39% of outcome data at 5yrs and 10% of outcome data at 2 years were missing, as well as 18·5% of	Heterogeneity between centres was modelled through random intercepts.	Reference was LEAT which was a mixture of LGNET, DNET, gangliogliomas and others.<br>Vascular is a mixture of cavernomas and other vascular malformations. Other mixed categories are MCD-other, LEAT, and non-LEAT and Encephalitis. These are rated down.<br>-1		Pooled data from 37 centres.<br>"We acknowledge that not including patients of whom no histopathological data were available, such as those undergoing disconnection surgery, could have introduced a bias when assessing the association between cause and outcome."	Proportion with Engel I around 70% or greater had significant ORs and were concluded to be positively prognostic, whereas in the 50%s were negatively prognostic.		Adjusted for age at surgery and duration of epilepsy and lobe of surgery<br>+1	+++

	retrospective, multicentre, longitudinal, cohort study<br><br>random-effects logistic regression models<br><br>all ORs are cf to LEAT	672<br><br>DNET<br><br>484		74·8%	duration of epilepsy data -1)								
		vascular malformation (cavernomas and others)<br><br>443<br><br>Cavernomas<br><br>323<br><br>Others<br><br>120		74·0%<br><br>NS<br><br>OR 0.79 [0·60 - 1·06]<br><br>77·1%<br><br>65·8%									++
		hippocampal sclerosis<br><br>2948		71·5%<br><br>OR 0.79 [0·65 - 0·89]									+++
		FCD type I or MCD<br><br>426		Negative<br><br>50·0%<br><br>OR 0.38 [0·28 - 0·49]									+++
		Other MCD<br><br>(Hypothalamic hamartomas, tubers and others)		Negative<br><br>52·3%									++

		405		OR 0.44 [0·29 - 0·63]									
		No histopathological lesion (comprised of gliosis and normal tissue)<br>740		Negative<br>53·5%<br>OR 0.36 [0·30 - 0·46]									+++
		FCD type II<br>796		64·9%<br>NS<br>OR 0.8 [0·61 - 1·09]									+++
		Encephalitis<br>(rasmussen's and limbic, herpes, neurocysticercosis)<br>124<br>Encephalitis - Rasmussen's subgroup<br>72		59·7%<br>OR 0.43 [0·22 - 0·73]<br>72·2%									++
		Glial scar<br>261		59·4%<br>OR 0.53 [0·39 - 0·70]									+++
		Non-LEAT (astrocytoma, oligodendroglioma, cysts, ependymoma,		68·4%<br>NS									++

		meningioma, neurocytoma, and pleomorphic xanthoastrocytoma)<br><br>310		OR 0.75 [0·54 - 1·02]									
		Year of surgery ? but rated +1 as likely >1000		NS									+++
		Duration in years for LEAT<br><br>Duration interaction with all other pathologies		0.97 [0·96 – 0·99]<br><br>NS									+++
		Lobe of surgery<br><br>TL reference had the highest compared to all other lobes, all significant (multilobar, parietal, occipital, frontal, hypothalamus)<br><br>? (rated +1 as likely >1000)		Significant no effect size									+++

Table 1: Individual Meta-Analyses of postsurgical prognostic features, with quality of evidence rating using the GRADE system for each meta-analysis

[1]**Study limitations (risk of bias or internal validity):** Differential surveillance for outcome between studies, failure of accurate measurement of all known prognostic factors and to match for prognostic factors and/or lack of adjustment in statistical analysis. Includes selective reporting bias. [12 14]

[2]**Inconsistency of results:** If some studies suggest substantial prognostic value using relative measures while others suggest no effect or negative prognostic value then it may be appropriate to rate down for quality. Criteria for evaluating consistency include similarity of point estimates, extent of overlap of confidence intervals, and statistical criteria including tests of heterogeneity and I^2. If inconsistent results cannot be explained by differences in subgroups (populations undergoing surgery, surgical intervention, or outcome definitions and follow-up), then the quality of the body of evidence is rated down. [15]

[3]**Indirectness of evidence:** If there are differences in the populations, interventions and/or outcomes being studied compared to what we are interested in, or if interventions are compared without direct head-to-head comparisons, studies can be rated down [16]. We only rate down if there is a compelling reason to believe the populations studied differ from the population of interest that the magnitude of effect would differ significantly. We rate down if there is an outcomes discrepancy, whereby the seizure-freedom duration of follow-up in the inclusion criteria is less than that of interest (at least 12months). We consider ILAE 1 and 2 seizure free, which is equivalent to Engel Ia/Ib (we consider undifferentiated Engel I otherwise not reported to be seizure-free) and only rate down inclusion criteria that specifically include Engel II or ILAE 3.

[4]**Imprecision:** e.g. for a single meta-analysis, effect sizes which overlap the neutral point (for RR and OR, 1) suggesting the feature is not prognostic, but the boundaries of the confidence interval are skewed significantly in one direction such that the largest plausible effect is that the feature is either positively or negatively correlated with outcomes. [11]

[5]**Publication bias:** clinical features that are non-prognostic and smaller effect sizes are less likely to be published and these can be assessed by funnel plots. Cumulative iterative meta-analyses could be indirectly inferred from the publication dates to ascertain time-lag bias. Risk of publication bias is probably larger for small, observational, and industry-funded studies. [14]

[6]**Rating up:** relative risks above 2 (below 0.5) are rated up one level, and above 5 (below 0.2) are rate up two levels unless the CI overlaps significantly with these thresholds. If the baseline proportion of outcomes is low, odds ratios are treated similarly, otherwise a higher threshold is used. Studies were also rated up if a dose response was present or if all plausible residual confounders or biases would reduce a demonstrated effect, or suggest a spurious effect when results show no effect [17]

TLE: Temporal Lobe Epilepsy. ETE: Extratemporal lobe Epilepsy. APOS: Acute Postoperative Seizures. NS: Not Significant. CI: confidence aura. HS: Hippocampal Sclerosis. MCD: Malformations of Cortical Development. HH: Hypothalamic Hamartoma. TS: Tuberous Sclerosis. OR: Odds Ratio. RR: Relative Risk Ratio. SF: Seizure Freedom. NMA: Network Meta-Analysis. ATL: Anterior Temporal Lobectomy. SAH: Selective Amygdalohippocampectomy. NA: Not Available.

*: Our calculated CI from their data. [C]: Effect size derived from article data. [u]: Univariate analyses. [m]: Multivariate analyses. †A weakness of the Cochrane review is that they “did not class any of the pre-operative prognostic factors of interest…as confounders” so in general our GRADE score is one lower.

2.1.50 Remick, Ibrahim (63) (2020)

**Meta-analysis**<br><br>***Publication year***<br><br>**Years of individual studies**	**# included Studies, Patients**<br><br>**Outcomes, Follow-up Durations**<br><br>**Model(s)**	**Feature**<br><br>**# of total patients with and without**<br><br>**(# of studies)**	**Population:**<br><br>**Lobe**<br><br>**Age**	**Effect Sizes (seizure freedom)**	**Rating the quality of the meta-analysis evidence using the GRADE guidelines** [13]								
					**Risk of Bias or Internal Validity**[1]	**Inconsistency of Results**[2]	**Indirectness of Evidence**[3]	**Imprecision**[4]	**Publication bias**[5]	**Large effect size?**[6]	**"Dose" response?**[6]	**All plausible residual confounding?**[6]	**Quality of the body of evidence (GRADE)**
Remick, Ibrahim (63) (2020)<br><br>2020<br><br>1999 – 2018	33<br><br>462<br><br>Mixed effects meta analysis<br><br>PCA and multivariable logistic regression of principle components	SEEG vs SDE<br><br>SEEG 127/235 (17)<br><br>SDE 146/227 (18)<br><br>(33)	All	NS<br><br>SDE 64.3% [61.1, 67.5]<br><br>SEEG 54% [50.8, 57.3]<br><br>It is likely adjusted p value in the text is p = 0.0565<br><br>OR[cu] 0.65 [0.45, 0.95] p=0.025[cu]	Cox proportional hazards investigated length of follow-up<br><br>-1 some outcome data unavailable for SEEG and SDE<br><br>"the difference between seizure freedom rates following SEEG- or SDE-informed resection decreased with long-term follow-up"	-1<br><br>"SEEG-informed resections were associated with a lower rate of postresection seizure freedom than SDE-informed resections (p = 0.0247)."<br><br>But also<br><br>"Our results demonstrate that while there was no difference in seizure freedom rates regardless of resection (p = 0.0565)"	-2 does not include studies directly comparing SEEG vs SDG						+<br><br>Very low

2.2 Supplementary Table 2: Individual Prognostic Features Across All Meta-Analyses

**Feature**	**Population**<br><br>**# patients (# studies, #meta-analyses)**	**Effect Sizes**	**Rating the quality of the meta-analytic evidence behind the potential prognostic value using GRADE guidelines** [13]								
			**Risk of Bias or Internal Validity**[1]	**Inconsistency of Results**[2]	**Indirectness of Evidence**[3]	**Imprecision**[4]	**Publication bias**[5]	**Large effect size?**[6]	**"Dose" response?**[6]	**All plausible residual confounding?**[6]	**Quality of the body of evidence**
Example	*TLE, FLE…*<br><br>*Age*<br><br>*Other*	*RR, OR…*	*Heterogenous outcome follow-ups*<br><br>*Exclusion of known prognostic factors and statistical adjustments*<br><br>*Selective Reporting*	*Widely spread effect sizes as assessed by point estimates, CI, and statistical tests of heterogeneity.*	*Populations, interventions and/or outcomes being studied differ from those of interest:*<br><br>*e.g.: unseparated paediatric and adult ages, less than 12 months or more than ILAE 2*	*Large and/ or skewed CI*	*"undetected"*<br><br>*"suspected"*<br><br>*-1*<br><br>*"strongly suspected"*<br><br>*-2*	*at least a two-fold reduction or increase in risk*<br><br>*+1*<br><br>*5-fold or more increase in RR*<br><br>*+2*	*+1*	*All plausible residual confounders or biases would reduce a demonstrated effect, or suggest a spurious effect when results show no effect.*<br><br>*+1*	*+ Very Low*<br><br>*++ Low*<br><br>*+++ Moderate*<br><br>*High ++++*
**1. Clinical Features**											
Low IQ<br><br>IQ ≤75<br><br>"mental retardation"	TLE<br><br>Age ≥16 yrs<br><br>With and without structural lesions<br><br>1034 (8, 1)<br><br>non-HS structural lesions in TLE >16 yrs<br><br>150 (8, 1)<br><br>Tuberous sclerosis 108 (4)	RR 0.66 [0.54, 0.94]*<br><br>IQ on average 2.3 lower in not seizure-free group (p<0.009)<br><br>RR 0.26 [0.14, 0.50]*<br><br>NS OR 0.74 [0.33, 1.64]	Adjusted for presence of structural lesions Chelune, Naugle (20)<br><br>Absence of significant interaction with centres, or duration of epilepsy Chelune, Naugle (20)	Multicentre not meta-analysis Chelune, Naugle (20)	-2 duration of follow-up and definition of seizure freedoms differ Chelune, Naugle (20)<br><br>-1Heterogenous definitions of seizure freedom without sensitivity analyses. No definition of mental	No CI provided, but could be estimated from data presented Chelune, Naugle (20)	-1 suspected, untested Chelune, Naugle (20) Zhang, Hu (33)		+1<br><br>Higher seizure-freedom rates in higher IQs (their table 3)	+1<br><br>Adjusted for lesions	+<br><br>Chelune, Naugle (20) 1998<br><br>+

					retardation. Zhang, Hu (33)						Zhang, Hu (33) (2013)
Severe developmental delay	Tuberous Sclerosis; at least 90% less than 19 years old <181 (<20)	OR 0.14 [0.04, 0.48]	-1 Small samples (median 7, IQR[3,25]), could not adjust Fallah, Guyatt (35)			-1 Wide CI Fallah, Guyatt (35)	-1 Small samples, did not assess heterogeneity or bias Fallah, Guyatt (35)	+1 OR< 0.25			++ Fallah, Guyatt (35) (2013)
Pre-operative IQ (note all the others are severe low IQ)	TS in 90% <19yrs	NS OR[u] 1.01 [0.94, 1.08]									+ Fallah, Guyatt (35) (2013)
Moderate to severe developmental delay	Paediatric Rasmussen's <187 (<19)	NS HR[u] 0.64 [0.31, 1.32]	-1 Reporting bias: 7 out of 19 studies had ≤ 5 patients				-1 suspected but "unable to measure between-study heterogeneity and publication bias due to the very limited sample size per study"				+ Harris, Phillips (56) 2019
	Children and adults hemispherectomy 1041 (26)	OR 0.61, 95% CI									+++ Hu, Zhang (46) 2016

		0.46–0.82, p = 0.001									
History of Head injury	Adults and children 551 (7)	NS<br><br>RR 0.99 [0.86, 1.13]	-1 †	-1<br><br>I^2 46%, p 0.08, subgroup analyses by outcome were very different and inconsistent							+<br><br>West, Nevitt (3) 2019
febrile convulsions	TL and ET<br><br>Children and adults 1368 (20, 2)	OR 2.08 [1.2, 3.7], RR 1.09 [1.01, 1.17]	-1 †	Q=7.9, p=0.093 Tonini, Beghi (24)<br><br>-1 I^2 = 32%, p=0.11; >1 yr SF subgroup had good outcomes with febrile seizures but the other subgroups did not (subgroup I^2 49%, p 0.14) West, Nevitt (3) 2019	-1<br><br>Engel outcomes in 22 studies and other definitions in 25						+, +<br><br>Tonini, Beghi (24) (2004), West, Nevitt (3) 2019
CNS infections	TL and ET<br><br>Children and adults ? (2, 1)	NS<br><br>OR 0.73 [0.29, 1.82]	-1<br><br>Only 2 studies	Q=2.1, p=0.146	-1<br><br>Engel outcomes in 22 studies and other definitions in 25						+<br><br>Tonini, Beghi (24) (2004)
Focal (partial) seizure semiology (vs generalized)	ET, Adults, Non-lesional<br><br>62 (?, 1)	NS[u] (univariate Fisher's)	-1<br><br>Small sample sizes form multiple centres, heterogenous outcome reporting Ansari, Tubbs (18)				-1				+<br><br>Ansari, Tubbs (18) (2010)<br><br>++

	Adults and Children with FCD 2014(10, 1)	OR 1.46 [1.18, 1.82]					No funnel plots/trim fill etc Rowland, Englot (29) Zhang, Hu (33) Fallah, Guyatt (35)				Rowland, Englot (29) (2012)
	Adults and Children with FLE 269 (<21, 1)	NS P=0.05				-1 Magnitude not provided and p value borderline Englot, Wang (30)					+ Englot, Wang (30) (2012)
	TL in Children 425 (11, 1)	OR 1.36 [1.20, 1.56]					Undetected Englot, Rolston (32)				++ Englot, Rolston (32) (2013)
	Tuberous Sclerosis 65 (6)	NS OR 1.15 [0.42, 3.11]			-1 Heterogenous definitions of seizure freedom without sensitivity analyses Zhang, Hu (33)						+ Zhang, Hu (33) (2013)
			-1 Small samples (median 7, IQR[3,25]), could not adjust Fallah, Guyatt (35)		-1 Small samples, did not assess heterogeneity or bias Fallah, Guyatt (35)						+ *Fallah, Guyatt (35) (2013)
	Tuberous Sclerosis in at least 90% less than 19 years old (~children) 181 (20)	OR = 3.1 [1.2, 8.2] But same data NS PLS method				-1 Fig 1 shows bootstrapping CI just about crosses zero, otherwise significant prognostic value. Ibrahim, Morgan (36)				+1 Permutation testing was performed\to evaluate the significance of the component, and bootstrapping was used to identify significant contributors to the component. PLS accounted for latent structure of data and ordinal Engel outcomes classes. Ibrahim, Morgan (36)	++ *Ibrahim, Morgan (36) (2015)

…Focal vs generalized…<br><br>Focal onset with impaired awareness vs aware or other seizures	Repeat surgery for focal DRE 145 (?)<br><br>Repeat surgery for focal DRE 206 (?)<br><br>Paediatric Rasmussen <187 (<19)<br><br>Paediatric ET<br><br>206 (11)	NS^{u} OR^{c*} 1.84 [0.93, 3.61]<br><br>NS^{u*} OR 1.61 [0.93, 2.8]<br><br>p=0.089<br><br>NS HR 1.125 [0.66, 2.33]	-1 Reporting bias: 7 out of 19 studies had ≤ 5 patients			1 imprecise and effect sizes and CIs estimated. Krucoff, Chan (50) (2017)	-1 suspected but “unable to measure between-study heterogeneity and publication bias due to the very limited sample size per study”				*=same data different methods<br><br>+<br><br>Krucoff, Chan (50) (2017)<br><br>+<br><br>Krucoff, Chan (50) (2017)<br><br>+<br><br>Harris, Phillips (56) 2019<br><br>+<br><br>Englot, Breshears (37) 2013<br><br>++

	Children and adults hemispherectomy<br><br>403 (15)<br><br>Adults with supratentorial low grade gliomas<br><br>796 (3)<br><br>Children hemispherectomy<br><br>212(8)	OR 1.61 [1.18, 2.35]<br><br>OR 1.84, [1.18, 2.89], p = 0.008<br><br>RR 0.76 [0.67, 0.85]<br><br>NS[u]	Note the caption on Fig 3 seems incorrect and confusing.								Hu, Zhang (46) 2016<br><br>+<br><br>Shan, Fan (53) 2018<br><br>++<br><br>Cao, Liu (43) (2016)
Infantile/epileptic spasms	Tuberous sclerosis <343 (<27, 2), 90% <19yrs in ref [35]	OR 0.45 [0.24, 0.85,<br><br>NS OR 0.84 [0.35, 2.03] also NS on PLS	-1 Small samples (median 7, IQR[3,25]), could not adjust Fallah, Guyatt	I^2=45% Zhang, Hu (33) (2013)	-1 Heterogenous definitions of seizure freedom without sensitivity analyses Zhang, Hu (33) (2013)		-1 No funnel plots Zhang, Hu (33) (2013)<br><br>-1 Small samples, did not assess heterogeneity or bias Fallah, Guyatt			+1 Permutation testing was performed\to evaluate the significance of the component, and bootstrapping was used to identify significant contributors to the component. PLS accounted for latent structure of data and ordinal Engel outcomes classes. Ibrahim, Morgan (36)	+, +<br><br>Zhang, Hu (33) (2013)<br><br>*Fallah, Guyatt (35) (2013)<br><br>+++<br><br>*Ibrahim, Morgan (36) (2015)

											* = same data different methods
Gender (male vs female)	Adults and Children with FLE <1199 (<21,1)<br><br>Children with TLE 553 (14, 1)<br><br>Tuberous Sclerosis <186 (<30, 2)	NS p=0.99 Males 53% females 54%<br><br>NS[u] crude OR[c] 1.22 [0.90, 1.85]<br><br>NS OR 0.94 [0.52, 1.71], NS 0.92 [0.40, 2.08] also NS on PLS	-1 Heterogenous definitions of seizure freedom without sensitivity analyses Zhang, Hu (33)<br><br>-1 Small samples (median 7, IQR[3,25]), could not adjust Fallah, Guyatt				Funnel plots: undetected Englot, Wang (30)<br><br>-1 No funnel plots Zhang, Hu (33)<br><br>-1<br><br>Small samples, did not assess heterogeneity or bias Fallah, Guyatt			+1 Permutation testing was performed\to evaluate the significance of the component, and bootstrapping was used to identify significant contributors to the component.<br><br>PLS accounted for latent structure of data and ordinal Engel outcomes classes	++<br><br>Englot, Wang (30) (2012)<br><br>+<br><br>Englot, Rolston (32) (2013)<br><br>+, +<br><br>Zhang, Hu (33) (2013) *Fallah, Guyatt (35) (2013)<br><br>+++<br><br>*Ibrahim, Morgan (36) (2015)<br><br>*=same data different methods<br><br>+

	MRI neg TLE<br><br>146 (11)<br><br>Repeat surgery in focal DRE 140 (?)<br><br>Paediatric ET 303 (15)<br><br>Children and adults hemispherecromy 575 (24)	NS OR 1.44 [0.86, 2.41] P=0.17<br><br>NS OR[c*] 0.83 [0.42, 1.64]<br><br>NS[u] OR[c*] 1.16 [0.74, 1.83]<br><br>NS OR 1.15, 95% CI 0.79–1.67, p = 0.46	-1 2/11 studies had zero SF cases amongst males and 5 male and 5 females each or 2 males and 5 females – very few numbers with large CI. Collected data from even studies with very few cases. NOS scores ranged from 4 to 6 stars Wang, Zhang (44)			-1 imprecise and effect sizes and CIs estimated					Wang, Zhang (44) (2016)<br><br>+<br><br>Krucoff, Chan (50) (2017)<br><br>+<br><br>Englot, Breshears (37) 2013<br><br>++<br><br>Hu, Zhang (46) 2016<br><br>+<br><br>Shan, Fan (53) 2018

	sex in low grade gliomas in adults <2641 (<23)<br><br>children hemispherectomy 231 (10)	NS NA<br><br>NS^{u} NA										++<br><br>Cao, Liu (43) (2016)
Seizure Frequency<br><br>Without daily seizures	Adults and children with FLE <1199 (<21, 1)<br><br>TL in Children<br><br>103 (5)<br><br>Paediatric ET 158 (4)	NS^{u}<br><br>OR^{c*} individual participant 2.98 [1.24, 7.16]]<br><br>NS OR^{uc*} 1.85 [0.93, 3.57]	-1<br><br>Limited info Englot, Wang (30)<br><br>small number of studies reported this: no formal CMH Meta-Analysis attempted Englot, Rolston (32)									+<br><br>Englot, Wang (30) (2012)<br><br>+<br><br>Englot, Rolston (32) (2013)<br><br>+<br><br>Englot, Breshears (37) 2013
Age at seizure onset	ET, Adults, Non-lesional	NS	-1Small sample sizes form multiple									+

	131 (?, 1)		centres, heterogenous outcome reporting								Ansari, Tubbs (18) (2010)
seizure onset before 12 months of age (dichotomised)	Tuberous sclerosis 200 (10, 1)	OR 0.47 [0.24, 0.92]		I^2=0% Zhang, Hu (33) (2013)	-1 Heterogenous definitions of seizure freedom without sensitivity analyses Zhang, Hu (33) (2013)		-1 No funnel plots Zhang, Hu (33) (2013)				+ Zhang, Hu (33) (2013)
Log base 10 of age at onset	Tuberous Sclerosis in 90% <19 yrs <181 (<20)	NS OR 1.52 [0.77, 2.99] also NS on PLS	-1 Small samples (median 7, IQR[3,25]), could not adjust Fallah, Guyatt				-1 Small samples, did not assess heterogeneity or bias Fallah, Guyatt			+1 Permutation testing was performed\to evaluate the significance of the component, and bootstrapping was used to identify significant contributors to the component. PLS accounted for latent structure of data and ordinal Engel outcomes classes. Ibrahim, Morgan (16	+, +++ *Fallah, Guyatt (35) (2013) *Ibrahim, Morgan (36) (2015) *=same data different methods
<18 yrs vs >18 yrs	MRI neg TLE 78 (6)	NS OR 1.09 [0.38, 3.07]	NOS scores ranged from 4 to 6 stars								+ Wang, Zhang (44) (2016)
Younger age at onset	Paediatric Rasmussen's <187 (<19)	NS HR 0.91^{u} [0.85, 0.96] HR 0.95^{m} [0.87, 1.04]	-2 Adjusted for the variable length of follow-up, but not lesional or other known factors. Reporting bias: 7 out of 19 studies				-1 suspected but "unable to measure between-study heterogeneity and publication bias due to the very limited				+ Harris, Phillips (56) 2019

	Paediatrics (and paedaitric subgroups: TL, but not ET, hemispherectomy, tumors or MCD)<br><br>? (<30)<br><br>ET non lesional children<br><br><95 (<17)<br><br>Children hemispherectomy <380 (13)	Meta Regression overall = $e^{0.346}$ = OR[c]=1.41 (p<0.001)<br><br>TL $e^{0.144}$= OR[c]=1.15 (p=0.023)<br><br>NS[u]<br><br>SMD = 0.26, [0.03, 0.49]<br><br>P = 0.028	had ≤ 5 patients		-1<br><br>SF not clearly defined, some individual studies included Engel Classes I and II		sample size per study”				++<br><br>Widjaja, Jain (60) 2020<br><br>+<br><br>Ansari, Maher (28) 2010<br><br>Cao, Liu (43) (2016)
Age at epilepsy surgery<br><br>Continuous	ET, Adults, Non-lesional<br><br>131 (?, 1)	NS (ANOVA)	-1<br><br>Small sample sizes form multiple centres, heterogenous outcome reporting								+<br><br>Ansari, Tubbs (18) (2010)

<18 yrs vs >18yrs	Adults and Children with FCD <2014 (13, 1)	NS<br><br>OR 1.14 [0.96, 1.35]					-1<br><br>No funnel plots/trim fill Rowland, Englot (29) Zhang, Hu (33)				++<br><br>Rowland, Englot (29) (2012)
<18 yrs vs >18yrs	Adults and Children with FLE <1199 (<21, 1)	NS<br><br>43% vs 54% p=0.22 (OR[c] ~0.64)					Funnel plots: undetected Englot, Wang (30)				++<br><br>Englot, Wang (30) (2012)
Continuous	Children with TLE <1318 (17, 1)	NS t-test[u]									+<br><br>Englot, Rolston (32) (2013)
<5yrs vs >5yrs<br><br>Log base 10 age at surgery	Tuberous Sclerosis <375 (<31, 2)	NS OR 1.05 [0.58, 1.88]; NS OR 1.21 [0.56, 2.62]<br><br>Also NS on PLS	-1 Small samples (median 7, IQR[3,25]), could not adjust Fallah, Guyatt		-1 Heterogenous definitions of seizure freedom without sensitivity analyses Zhang, Hu (33)		-1 Small samples, did not assess heterogeneity or bias Fallah, Guyatt			+1 Permutation testing was performed\to evaluate the significance of the component, and bootstrapping was used to identify significant contributors to the component. PLS accounted for latent structure of data and ordinal Engel outcomes classes. Ibrahim, Morgan (36)	+, +<br><br>Zhang, Hu (33) (2013), Fallah, Guyatt (35) (2013)<br><br>+++<br><br>Ibrahim, Morgan (36) (2015)<br><br>+

<18 yrs vs > 18 yrs at surgery	MRI neg TLE 78 (6)	NS OR 1.09 [0.38, 3.07]	NOS scores ranged from 4 to 6 stars Wang, Zhang (44) (2016)									Wang, Zhang (44) (2016)
Subgroup meta-regression: mean age at surgery	TLE and ET children and adults <1983 (<17)	NS		Not detected. Subgroup analyses for paediatric, time of occurrence, semiology and meta-regressions to explore heterogeneity were performed. Giridharan, Horn (45)			-1 Funnel plots and Egger's regression showed no bias. However, we note asymmetry in overall APOS, paediatric APOS and semiology group funnel plots in their Fig e1. Giridharan, Horn (45)					++ Giridharan, Horn (45) 2016
Age<18 yrs	Occipital Lobe and posterior quadrant. Mixed adult and paediatric 111 (7)	OR 1.54 [1.13, 2.18]	Attempted to minimise selection bias: variables selected only if at least 80 patients across 5 studies. -1 No statistical adjustments "impossible to perform a multivariate analysis looking for interactions across variables" e.g. didn't adjust for lesions	Salanova 1992 study seems to have an outlying large point effect for age, without attempts at subgroup explanation. Harward, Chen (49)								+ Harward, Chen (49) 2017
Age at surgery	Repeat surgery focal DRE<br>1st surgery 194 (?)<br>2nd/last surgery 164 (?)	NS[u]				-1 imprecise and effect sizes and CIs estimated. Krucoff, Chan (50)						+ Krucoff, Chan (50) (2017)

											+<br>Harris, Phillips (56) 2019
	Paediatric Rasmussen's <187 (<19)	HR 0.93[u] [0.89, 0.97] NS[m] HR 0.95[m] [0.90, 1.0]	-2 Adjusted for the variable length of follow-up, but not lesional or other known factors. Reporting bias: 7 out of 19 studies had ≤ 5 patients				-1 suspected but "unable to measure between-study heterogeneity and publication bias due to the very limited sample size per study"				
											++<br>Widjaja, Jain (60) 2020
	Paediatric Meta Regression ? (<30)<br>Overall<br>TL<br>ET<br>But NS for hemispherectomy, tumors, MCD	Meta regression<br>$e^{-0.189}$ = OR[c] = 0.83 overall p<0.001<br>$e^{-0.093}$ = OR[c] = 0.91 TL p=0.031<br>$e^{-0.173}$ = OR[c] = 0.84 ET p 0.004							+1 meta-regression		+<br>Englot, Breshears (37) 2013
	Paediatric ET<br><1259 (17)										+<br>Shan, Fan (53) 2018

Age>= 45 yrs	Low grade gliomas in adults 1065 (6)<br><br>ET non lesional children<br><br><95 (<17)<br><br>Children hemispherectomy <380(13)<br><br>Cavernomas adults and children >18 vs other <245 (<7)	NSᵘ<br><br>RR 1.12 [1.01, 1.23]<br><br>NSᵘ<br><br>NSᵘ<br><br>NS 0.95 [0.38, 2.37]									+<br><br>Ansari, Maher (28) 2010<br><br>++<br><br>Cao, Liu (43) 2016<br><br>+<br><br>Shang-Guan, Wu (54) (2018)
Duration of epilepsy prior to surgery	ET, Adults, Non-lesional<br><br>131 (?, 1)	NS<br><br>NS NA	-1<br><br>Small sample sizes form multiple centres, heterogenous outcome reporting<br><br>-1				Funnel plots: undetected				+<br><br>Ansari, Tubbs (18) (2010)<br><br>+

Mean duration	Adults and Children with FLE <1199 (<21, 1)	NS t-test	Limited information given								Englot, Wang (30) (2012)
Shorter epilepsy duration	TLE in Children <1318 (12, 1)	OR = 2.57 [1.21, 5.47]	NOS scores ranged from 4 to 6 stars								++<br>Englot, Rolston (32) (2013)
	MRI neg TLE 128 (9)										+<br>Wang, Zhang (44) (2016)
Subgroup meta-regression: mean duration of epilepsy	TLE and ET children and adults <1983 (<17)	NS		Not detected. Subgroup analyses for paediatric, time of occurrence, semiology and meta-regressions to explore heterogeneity were performed. Giridharan, Horn (45)			-1<br>Funnel plots and Egger's regression showed no bias. However, we note asymmetry in overall APOS, paediatric APOS and semiology group funnel plots in their Fig e1. Giridharan, Horn (45)				++<br>Giridharan, Horn (45)<br>2016
	Repeat surgery for focal DRE<br>60 (?)	NS^{u} (t-test)									+<br>Krucoff, Chan (50) (2017)
Time between resections		NS^{u} (t-test)									+<br>Krucoff, Chan (50) (2017)

Shorter duration of epilepsy	Repeat surgery for focal DRE 188 (?)<br><br>Paediatric Rasmussen's <187 (<19)	HR 0.92[u] [0.88, 0.97] (NS likely on adjustment)	-2 Not even adjusted for the variable length of follow-up, let alone not lesional or other known factors. Reporting bias: 7 out of 19 studies had ≤ 5 patients				-1 suspected but "unable to measure between-study heterogeneity and publication bias due to the very limited sample size per study"				+<br><br>Harris, Phillips (56) 2019
<2 vs >2yrs 388 (3)<br><br><5 vs >5yrs 551 (4)<br><br><10 vs >10 1376 (10)<br><br><20 vs <20 346 (3)	Children and adults all lobes	RR 1.20 [1.05, 1.39]<br><br>RR 1.24 [1.08, 1.42]<br><br>1.25 [1.09, 1.43]<br><br>1.33 [1.08; 1.65]	"Moderate risk of bias"<br><br>"did not justify downgrading the evidence level"	-1<br><br>3/12 studies reported no association between duration and outcome<br><br>1/12 study was in favour of longer duration			Studies that only reported mean or median duration of epilepsy for patients grouped by seizure outcome were not included.		Evaluated<br><br><5 yrs vs >10yrs<br><br>To investigate if a larger time gap in epilepsy duration resulted in a larger effect – not present		+<br><br>Bjellvi, Olsson (57) (2019)<br><br>Except for <10 vs >10 yrs:<br><br>++<br><br>Bjellvi, Olsson

<5 vs >10 430 (4)		1.32 [1.19; 1.46]									(57) (2019) +
Shorter epilepsy duration (≤ 7 years, the median value in this study)	Paediatric ET <1259 (8)	OR 1.52 [1.07, 2.14]									Englot, Breshears (37) 2013 +
More than 1 year history of seizures	Adults with low grade gliomas <2641 (23)	RR 0.82 [0.75, 0.91]									Shan, Fan (53) 2018 +
	ET nonlesional children <95 (<17)	NS[u]									Ansari, Maher (28) 2010 ++

	Children hemispherectomy <380 (5)<br><br>Cavernomas adults and children<br><br><245 (<7)	NS[u]<br><br>NS			-1<br><br>f/up 6 months						Cao, Liu (43) (2016)<br><br>+<br><br>Shang-Guan, Wu (54) (2018)
Duration in years	Children and Adults with low-grade epilepsy associated neuroepithelial tumour (LEAT)<br><br>Duration does not interact with any other pathology	0.97 [0·96 – 0·99]<br><br>NS									+++<br><br>Lamberink, Otte (62)
Postsurgical: without acute postoperative seizures (APOS) within 30days after surgery	TLE and ETE<br><br>Mixed paediatric and adult.<br><br>1983 (17)<br><br>Paediatric: 730 (6)<br><br>TLE and ET 222 (6)	Overall OR 4.2 [2.97, 5.93]<br><br>(Without APOS 73.5% seizure-free, vs with APOS 39%)<br><br>Paediatric subgroup OR 5.71 [3.32, 9.8]	Subgroup analysis for overall APOS persistent in both.<br><br>-1 64.8% had presurgical lesion, not adjusted<br><br>Variable APOS definitions (7-30 days) but used meta-regression to explore this for under 24hrs only	Not detected<br><br>Subgroup analyses for paediatric, time of occurrence, semiology and meta-regressions to explore heterogeneity were performed		-1 towards positive Seizure-freedom more	-1<br><br>Funnel plots and Egger's regression showed no bias. However, we note asymmetry in overall APOS, paediatric APOS and semiology group funnel plots in their Fig 1e.	Large effect size +1			++<br><br>*Giridharan, Horn (45)*<br><br>*2016*<br><br>+

Earlier onset of APOS (within 24hrs)		NS 1.87 [0.89, 3.95]				likely, but statistically not significant					*Giridharan, Horn (45)*<br>*2016*
Postsurgical semiology different from presurgical	TLE and ET adults and children 109 (3)	NS 4.24 [0.93, 19.25]		Not detected<br>Subgroup analyses for paediatric, time of occurrence, semiology and meta-regressions to explore heterogeneity were performed		-1 towards positive Seizure-freedom more likely, but statistically not significant	-1<br>Funnel plots and Egger's regression showed no bias. However, we note asymmetry in overall APOS, paediatric APOS and semiology group funnel plots in their Fig e1.				+<br>Giridharan, Horn (45)<br>*2016*
epilepsia partialis continua (EPC)	Children undergoing hemispherectomies 127 (7)	NS[u]									++<br>Cao, Liu (43) (2016)

**2. Imaging Features**											
**Consistently prognostic**											
mesial temporal sclerosis	TLE<br><br>Children and adults 4430 (61, 2)	OR 2.13 [1.57, 2.86] RR 1.17 [1.12, 1.23]	-1 †	Q=21.9, p=0.082	-1<br><br>Engel outcomes in 22 studies and other definitions in 25						++, ++<br><br>Tonini, Beghi (24) (2004), West, Nevitt (3) 2019
Abnormal MRI (lesional MRI)	Adults and children, TL and ET <13238 (<114, 4)<br><br>TL lesion vs TL no lesion <4785 (<51, 2)<br><br>ET lesion vs ET no lesion <3760 (<45, 2)	OR 2.27 [1.54, 3.45]; OR 2.5 [2.1, 3.0] (RR 1.4); OR 2.03 [1.67, 2.47], RR 1.28 [1.20, 1.37]<br><br>OR 2.7 [2.1, 3.5]; OR 1.76 [1.34, 2.32]<br><br>OR 2.9 [1.6, 5.1]; OR 2.88 [1.53, 5.43]	-1 Heterogenous SF definitions. English and Chinese studiesYin, Kang (31);<br><br>Two studies favoured non-lesional epilepsy Téllez-Zenteno, Ronquillo (5)<br><br>-1 †	One outlier with poor results but wide CI [24]<br><br>Q=4.9, p=0.768<br><br>Q=35.6, p=0.43	-1 Engel outcomes in 22 studies and other definitions in 25 Tonini, Beghi (24); -1 short follow ups in 3 studies Yin, Kang (31).<br><br>Téllez-Zenteno, Ronquillo (5) also investigated whether lesion definition by MRI or histopathology made a difference – it didn't.		Non lesional significantly more frequent in ET cases (45%) than in TL (24%)[5]; undetected Yin, Kang (31)<br><br>-1<br><br>No funnel plots/trim fill Téllez-Zenteno, Ronquillo (5),				++, ++, ++, ++<br><br>Tonini, Beghi (24) (2004), Téllez-Zenteno, Ronquillo (5), Yin, Kang (31) (2013) *West, Nevitt (3) 2019*<br><br>++, ++<br><br>Téllez-Zenteno, Ronquillo (5), Yin, Kang (31) (2013)<br><br>++, +<br><br>Téllez-Zenteno, Ronquillo (5), Yin, Kang (31) (2013)

	Adults and Children with FCD <2014 (14, 1)	OR 1.67 [1.33, 2.16]					Funnel plots: undetected (not shown)Rowland, Englot (29)				++ Rowland, Englot (29) (2012)
	FLE, Adults and Children 627 (14, 1)	RR 1.64, [1.32, 2.08]									++ Englot, Wang (30) (2012)
	TL in Children 802 (26, 1)	OR 1.27 [1.16, 1.40]									++ Englot, Rolston (32) (2013)
	Occipital Lobe and posterior quadrant. Mixed adult and paediatric. 132 (7)	OR 3.24 [2.03, 6.55]	-1 No statistical adjustments "impossible to perform a multivariate analysis looking for interactions across variables" e.g. didn't adjust for lesions. Harward, Chen (49)	Liava 2014 is the only study out of 7 without a CI overlapping OR of 1.							+ Harward, Chen (49) 2017
	Repeat surgery for focal DRE 196 (7)	NS OR 1.9 [0.6, 5.4]		-1							++ Krucoff, Chan (50) (2017)

	Paediatric 883 (10)<br><br>Paediatric ET<br>506 (23)<br><br>Children hemispherectomy <380 (8)	OR 1.85<br>[1.14, 2.94]<br><br>NS<br>OR^{uc}* 1.44 [0.98, 2.12]<br><br>OR 4.6 [1.27, 16.62]	Sensitivity analysis performed by removing the single RCT from results.<br><br>NOS	SF not clearly defined, some individual studies included Engel Classes I and II		1 wide CI	Funnel and trim and fill: difference between observed and imputed <10%				+<br>Widjaja, Jain (60) 2020<br><br>+<br>Englot, Breshears (37) 2013<br><br>+<br>Cao, Liu (43) (2016)
Number of cortical tubers <=4 vs > 4<br>"less tuber burden"	Tuberous Sclerosis <286 (<24, 2)	NS OR 1.12 [0.49, 2.57];<br>NS OR 1.01 [0.96, 1.07] also NS on PLS	-1 Small samples (median 7, IQR[3,25]), could not adjust Fallah, Guyatt		-1<br>Heterogenous definitions of seizure freedom without sensitivity analyses Zhang, Hu (33)		-1<br>No funnel plots Zhang, Hu (33)<br>-1<br>Small samples, did not assess heterogeneity or			+1<br>Permutation testing was performed\to evaluate the significance of the component, and bootstrapping was used to	+, +<br>Zhang, Hu (33) (2013)<br>*Fallah, Guyatt (35) (2013)

							bias Fallah, Guyatt			identify significant contributors to the component.<br><br>PLS accounted for latent structure of data and ordinal Engel outcomes classes Ibrahim, Morgan (36) (2015)	+++<br><br>* Ibrahim, Morgan (36) (2015)
^{1}H spectroscopy:<br><br>magnetic spectroscopy abnormality<br><br>ipsilateral to lobe of resection	TLE adults and children<br><br>TLE, adults and children, normal MRI 121 (22, 1)	OR 4.9 [1.97, 12.17]<br><br>NS	Fifteen centers performed chemical shift imaging and seven centers used single-voxel spectroscopy. Most studies were obtained at 1.5 T	Q=2.7<br><br>Only valuable in lesional MRI cases	-2<br><br>The EZ was mostly defined by EEG data (rather than resection)	-1<br><br>Large CI		PPV = 82% but no benchmark			+<br><br>Willmann, Wennberg (26) (2006)<br><br>Probably no more valuable than conventional MRI abnormality
Vascular disorders	TL and ET<br><br>Children and adults ? (3, 1)	NS<br><br>OR 0.66 [0.30, 1.46]	-1<br><br>3 studies	Q=945, p=0.6	-1<br><br>Engel outcomes in 22 studies and other definitions in 25						+<br><br>Tonini, Beghi (24) (2004)

FDG-PET focal interictal hypometabolism	TLE and ET Adults<br><br>153 (46, 1)<br><br>TLE Adults ? (35, 1)<br><br>Adults and Children with FLE <1199 (<21,1)<br><br>MRI negative TLE 127 (5, 1)	NS[u] (unweighted crude)<br><br>NS[u] (unweighted crude)<br><br>NS[u] (Chi-squared tests then random effects if significant)<br><br>NS p=0.06<br><br>OR = 2.11 [0.95, 4.65]	-1<br><br>The analyses were complicated by significant differences in study design and often by lack of precise patient data.<br><br>the tracer injection dose from 1 to 15 mCi, and the time for data acquisition after tracer injection from 5 to 60 min<br><br>NOS scores 4-6 Wang, Zhang (44)				Funnel plots: undetected				+<br><br>Willmann, Wennberg (27) (2007)<br><br>PET does not appear to add value in patients localized by ictal scalp EEG and MRI.<br><br>+<br><br>Englot, Wang (30) (2012)<br><br>+<br><br>Wang, Zhang (44) (2016)
Encephalomalacia	Adults and children 317 (5)	NS<br><br>RR 0.78 [0.52, 1.17]	-1 †	No significant difference between outcome subgroups							+<br><br>West, Nevitt (3) 2019
Enhancement, oedema, mass effect	Low grade gliomas in adults <2641 (<23)	NS NA									+<br><br>Shan, Fan (53) 2018

SPECT: SISCOM concordance with resection area<br><br>subtraction ictal and inter-ictal SPECT co-registered to MRI (SISCOM)	TL and ET 275 (11)<br><br>ET subgroup 209 (11)	OR 3.28 [1.90, 5.67]<br><br>OR 2.44 [1.34, 4.43]		I^2 = 16.6%, p=0.285)<br><br>I^2 = 10.6%, Q = 10.06, p = 0.345			Egger's and Begg's test not significant				++<br><br>Chen and Guo (47) (2016)

3. Neurophysiological Features												
Postoperative discharges	TL and ET<br>Children and adults 1547 (9, 2)	OR 0.28 [0.08, 0.95]<br>NS Adjusted for outcomes RR 0.91 [0.68, 1.22]	-1<br>Only 3 studies	Heterogenous Q=6.7, p=0.035j used random effects	-1<br>Engel outcomes in 22 studies and other definitions in 25						+, ++<br>Tonini, Beghi (24) (2004), West, Nevitt (3) 2019
	TLE subgroup <542 (<6)	RR 0.81 [0.70, 0.94]	-1 †								
Intracranial / invasive<br>Monitoring / EEG<br>(performed vs not performed)	TL and ET<br>Children and adults 1547 (27, 2)	OR 0.37 [0.22, 0.63], RR 0.85 [0.78, 0.93]	1 †	Q=3,p=0.7	-1<br>Engel outcomes in 22 studies and other definitions in 25						+, ++<br>Tonini, Beghi (24) (2004), West, Nevitt (3) 2019
	ET, Adults, Non-lesional<br>108 (?, 1)	NS	-1<br>Small sample sizes form multiple centres, heterogenous outcome reporting								+<br>Ansari, Tubbs (18) (2010)
	Children and adults with FLE <1199, <21, 1)	NS	-1<br>Limited information provided				Funnel plots: undetected Englot, Wang (30) Englot, Rolston (32)				+<br>Englot, Wang (30) (2012)
	Tuberous Sclerosis 144 (7)	NS<br>OR 1.6 [0.76, 3.37]			-1<br>Heterogenous definitions of seizure freedom without sensitivity analyses Zhang, Hu (33)		-1<br>No funnel plots Zhang, Hu (33)				+<br>Zhang, Hu (33) (2013)

ECoG performed<br><br>Invasive monitoring	Children with TLE 462 (13)<br><br>Repeat surgery on focal DRE<br>210 (?)<br><br>Paediatric ET<br>433 (20)<br><br>ET nonlesional children <95 (<17)	NS OR[c] crude 1.31 [0.84, 2.04]<br><br>OR = 0.4, [0.2, 0.9]<br><br>NS[u]<br>OR[uc]* 0.77 [0.50, 1.19]<br><br>NS[u]									++<br>Englot, Rolston (32) (2013)<br><br>++<br>Krucoff, Chan (50) (2017)<br><br>+<br>Englot, Breshears (37) 2013<br><br>+<br>Ansari, Maher (28) 2010
sEEG vs subdural grid	TL and ET adults and children (but not from children only studies) 1999 (31)	Overall RR = 64.7% [59.2, 69.8] / 55.9% [50.9, 60.8] = 1.16[c]	-1 average follow up for SEEG was 10 months while for SDG it was	Overall SEEG: $I^2 = 11.86\%$; p = 0.318<br><br>subdural grid: $I^2 = 54.47\%$; p = 0.002	-1<br>studies <6 months follow-up durations; we are interested in		Funnel plots, Egger's tests: no overall changes or subgroup changes				+<br>Toth, Papp (59)<br>2019

	Nonlesional 237 (15)	Nonlesional NS RR = 52% [37.3, 66.3] / 54.4% [40.6, 67.6] = 0.96	nearly 19months.<br><br>Significant differences overall (p = 0.02), lesional (p = 0.031), and also, temporal sugroups (p = 0.002)		at least 12 months						
	Lesional 665 (21)	Lesional RR = 71.6% [61.6, 79.9] / 57.3% [48.7, 65.6] = 1.25									
	TL 470 (17)	TL RR = 73.9% [64.4, 81.6] / 56.7% [51.5, 61.9] = 1.30									
	ET 420 (14)	ET RR = (61% [51, 70.2]) / (46.7%[36.5, 57.2]) = 1.31									
	all	OR[cu] 0.65 [0.45, 0.95] p=0.025[cu]<br><br>It is likely adjusted p value in the text is p = 0.0565<br><br>Therefore NS	See individual paper Remick et al for GRADE scores for this feature								+<br>[63] 2020

Intraoperative ECoG	Children and adults with FLE 1024, <21, 1)	NS p=0.14<br><br>Pooled ind particicpant OR[c] 1.23 [0.95, 1.62]					Funnel plots: undetected (not shown)				+++<br><br>Englot, Wang (30) (2012)
Interictal spikes	TL and ET<br><br>Children and adults ? (3, 1)	NS<br><br>OR 1.82 [0.86, 3.88]	-1<br><br>Only 3 studies			-1<br><br>Skewed					+<br><br>Tonini, Beghi (24) (2004)
Lateralised/ unilateral interictal EEG	Children and adults with FLE <1199, <21, 1)	NS	-1 limited information provided Englot, Wang (30)				Funnel plots: undetected Englot, Wang (30)				+<br><br>Englot, Wang (30) (2012)
	Tuberous Sclerosis 127 (6)	OR 2.42 [1.11, 5.27]		I^2=0% Zhang, Hu (33) (2013)	-1 Heterogenous definitions of seizure freedom without sensitivity analyses Zhang, Hu (33) (2013)		-1 No funnel plots<br><br>Zhang, Hu (33) (2013)				+<br><br>Zhang, Hu (33) (2013)
Unilateral interictal spikes vs bilateral	Adults and children 1414 (18)	RR 1.14 [1.05, 1.24],	-1<br><br>CI adjusted for various outcome scale<br><br>Definition was likely to have influenced the analysis, e.g. non-lateralising vs contralateral spikes, focal vs non-focal spikes †	-1 I^2 = 67% overall and mixed effects using only Engel outcomes is non-significant: RR [0.88, 2]. Subgroup analyses (TLE vs ET) do not explain these differences.		-1 small studies with imprecise results. Mixed-effects model RR has no statistical significance.			In best case scenario of pooled effect rather than mixed-effects, the point estimation is 14% better. E.g. if bilateral 60% SF, unilateral spokes, 70% SF. This variable only explains one-third of the missing outcome variance, with		+<br><br>West, Nevitt (3) 2019

									NNT around 10.		
	Paediatric ET<br>130 (10)	NS<br>OR^{uc}* 2.22 [0.98, 5.05]									+<br>Englot, Breshears (37) 2013
	Adults and children hemispherectomy 413 (7)	OR 1.66, [1.03, 2.67]									++<br>Hu, Zhang (46) 2016
Unifocal interictal scalp EEG abnormality (or no interictal abnormality)	Tuberous Sclerosis 90% <19yrs <181 (<20,1)	NS OR 1.54 [0.73, 3.26]<br>Also NS on PLS	-1<br>unusual feature dichotomization<br>-1 Small samples (median 7, IQR[3,25]), could not adjust Fallah, Guyatt			-1 Skewed bootstrapping CI suggestive of possible positive effect Ibrahim, Morgan (36)	-1<br>Small samples, did not assess heterogeneity or bias Fallah, Guyatt			+1<br>Permutation testing was performed\to evaluate the significance of the component, and bootstrapping was used to identify significant contributors to the component. PLS accounted for latent structure of data and ordinal Engel outcomes classes	+<br>*Fallah, Guyatt (35) (2013)<br>++<br>*Ibrahim, Morgan (36) (2015)<br>* = same data different methods
Interictal EEG localised to temporal lobe	MRI neg TLE 149 (7)	OR 3.38<br>[1.57, 7.25]	NOS 4-6		- 1 large CI Wang, Zhang (44)						+

												Wang, Zhang (44) (2016)
Focal ictal/interictal/invasive EEG	Repeat resective surgery for focal DRE<br><br>192 (8)	OR = 3.6, [1.6, 8.2]										++<br><br>Krucoff, Chan (50) (2017)
Unilateral vs bilateral ictal EEG (Lateralized ictal EEG)	Adults and Children with FCD <2014 (10, 1)<br><br>Tuberous Sclerosis 159 (8)<br><br>Adults and children hemispherectomy 414 (7)	NS<br><br>OR 1.03 [0.82, 1.31]<br><br>OR 2.48 [1.17, 5.24]<br><br>ictal: OR 1.88, [1.15, 3.07], p = 0.01		I^2=0% Zhang, Hu (33)	-1 Heterogenous definitions of seizure freedom without sensitivity analyses Zhang, Hu (33)		-1<br><br>No funnel plots/trim fill Rowland, Englot (29) Zhang, Hu (33)				++<br><br>Rowland, Englot (29) (2012)<br><br>+<br><br>Zhang, Hu (33) (2013)<br><br>++<br><br>Hu, Zhang (46) 2016

Localized/unifocal ictal (scalp) EEG	Children and adults with FLE <1199, <21, 1)	NS^u	-1 limited information provided				Funnel plots: undetected				+ Englot, Wang (30) (2012)
	Children with TLE 445 (14, 1)	NS^u crude OR^c 1.23 [0.73, 2.06]									++ Englot, Rolston (32) (2013)
	Tuberous Sclerosis; at least 90% less than 19 years old <186 (<20)	OR = 3.21, [1.35–7.58] Positive prognostic value (PLS)	-1 Small samples (median 7, IQR[3,25]), could not adjust Fallah, Guyatt (15)				-1 Small samples, did not assess heterogeneity or bias Fallah, Guyatt (15)			+1 Permutation testing was performed\to evaluate the significance of the component, and bootstrapping was used to identify significant contributors to the component. PLS accounted for latent structure of data and ordinal Engel outcomes classes Ibrahim, Morgan (36)	+ *Fallah, Guyatt (35) (2013) +++ *Ibrahim, Morgan (36) (2015) * = same data, different methods
Ictal EEG localized to temporal lobe	MRI neg TLE 125 (6)	OR = 3.89 [1.66, 9.08]	NOS 4-6		- 1 large CI Wang, Zhang (44)						+ Wang, Zhang (44) (2016)
	Paediatric ET 226 (13)	OR 1.55 [1.24, 1.93]									+ Englot, Breshears (37) 2013

Video-Telemetry (VT)<br><br>(Long Term Monitoring, LTM)	Children and adults with FLE <1199, <21, 1)<br><br>TLE, ETE, lesoinal and nonlesional, (assumed adults and children as not explicitly mentioned)<br><br>(534, 44)<br><br>Lesional TLE<br><br>Lesional ETE<br><br>Nonlesional TLE<br><br>Nonlesional ETE	NS (chi-squared tests then random effects if significant)<br><br>OR 1.41 [0.79, 2.53]<br><br>OR 0.46 [0.2, 1.07]<br><br>OR 0.6 [0.01, 35.86]<br><br>1 [0.06, 17.51]	-1 limited information provided<br><br>A high risk of bias was observed in a considerable proportion of included studies; the quality of evidence was assigned as "very low"<br><br>Note the trend wotwards LTM predicting seizure free outcomes in lesional TLE only, which is a confounder of good outcomes in lesional TLE.				Funnel plots: undetected				+<br><br>Englot, Wang (30) (2012)<br><br>+<br><br>Very low<br><br>Kobulashvili, Kuchukhidze (55) (2018)

4. Multimodal Concordance											
EEG/MRI Concordance	TL and ET<br>Children and adults 1778 (29, 2)	OR 2.36 [1.07, 5.26]<br>RR 1.25 [1.15, 1.37]	-1 †	Heterogenous Q=11.4, p=0.044 used random effects	-1<br>Engel outcomes in 22 studies and other definitions in 25						+, ++<br>Tonini, Beghi (24) (2004), West, Nevitt (3) 2019
	Tuberous Sclerosis; at least 90% less than 19 years old <186 (<20, 2)	OR = 4.9, [1.8–13.5]<br>Positive prognostic value (PLS)	-1 Small samples (median 7, IQR[3,25]), could not adjust Fallah, Guyatt			-1<br>Wide CI Fallah, Guyatt (35)	-1 Small samples, did not assess heterogeneity or bias Fallah, Guyatt<br>undetectedIbrahim, Morgan (36) (2015)	+1<br>OR>4		+1<br>Permutation testing was performed\to evaluate the significance of the component, and bootstrapping was used to identify significant contributors to the component. PLS accounted for latent structure of data and ordinal Engel outcomes classes. Ibrahim, Morgan (36)	++<br>*Fallah, Guyatt (35) (2013)<br>+++<br>*Ibrahim, Morgan (36) (2015)<br>* = same data, different methods
Concordance same side	Children and adults hemispherectomy<br>332 (6)	OR 2.17 [1.30, 3.7]									++<br>Hu, Zhang (46) 2016

5. Genetics											

6. Surgical Features

Extensive surgical resection	TL and ET Children and adults <3511 (10, 1)	OR 4.27 [2.06, 8.85]		Q=26.9,p=0.001 used random effects	-1<br>Engel outcomes in 22 studies and other definitions in 25						++<br>Tonini, Beghi (24) (2004)
Extensive frontal +/- extra-frontal vs more localised frontal resections	FLE, adults and children 651 (11, 1)	RR 0.58 [0.41, 0.79]					Funnel plots: undetected Englot, Wang (30) Josephson, Dykeman (34)				++<br>Englot, Wang (30) (2012)
Lobectomy (extensive) vs tuberectomy (focal)	tuberous sclerosis<br>189 (10, 1)	OR 1.96 [1.01, 3.7]		I^2=0% as few numbers Zhang, Hu (33)	-1<br>Heterogenous definitions of seizure freedom without sensitivity analyses Zhang, Hu (33)	-1 CI approaches OR of 1 Zhang, Hu (33)	-1 No funnel plots Zhang, Hu (33)				+<br>Zhang, Hu (33) (2013)
ATL (extensive) vs SAH (selective)	TLE children and adults 1203 (11, 1)<br>TLE and HS subgroup children and adults 1092 (10, 1)	RR 1.32<br>[1.12, 1.57] (also quoted a separate random effects figure to the above fixed effects)<br>RR 1.26 [1.05, 1.51]	Result remained significant on multiple sensitivity analyses	I^2= 29%; df =10, p=0.17 Josephson, Dykeman (34)<br>I^2=0% Josephson, Dykeman (34)	children and adults – but also excluded paediatric only study and results were very similar Josephson, Dykeman (34)			summary risk difference 8 [3%–14%] translates to NNT of 13 [7, 33] for 1 additional patient to achieve an Engel Class I outcome following ATL Josephson, Dykeman (34)			+++<br>Josephson, Dykeman (34) (2013)
ATL vs SAH (mix of transcortical, transsylvian and subtemporal approaches)	Mainly adults ? (19)	NS OR 1.14, 95% CI 0.93 to 1.39; p=0.201									++<br>Jain, Tomlinson (52) 2018

ATL vs SAH	TLE 626 (6)	NS<br>RR 1.01 [0.54, 1.09]									+++<br>Kuang, Yang (38) (2013)
	SAH vs ATL in TLE 1397 (13)	Overall OR 0.65 [0.51, 0.82]					-1<br>Mild bias on funnel plot, did not further investigate or use trim and fill				++<br>Hu, Zhang (40) (2013)
Lesionectomy / multilobar resection surgery type <186 (<20)	Tuberous sclerosis children	NS on PLS								+1<br>Permutation testing was performed\to evaluate the significance of the component, and bootstrapping was used to identify significant contributors to the component. PLS accounted for latent structure of data and ordinal Engel outcomes classes. Ibrahim, Morgan (36)	+++<br>Ibrahim, Morgan (36) (2015)
Hemispherectomy (vs resective)	Rasmussen's<br>Paediatric <187 (<19)	HR 0.28^{u} [0.18, 0.45]<br>HR 0.30^{m} [0.18, 0.49]	-2 Adjusted for the variable length of follow-up, but not lesional or other known factors. Reporting bias: 7 out of 19 studies had ≤ 5 patients				-1 suspected but "unable to measure between-study heterogeneity and publication bias due to the very limited sample size per study"v			Remained significant on multivariate analysis but it seems only adjusted for length of follow up	+<br>Harris, Phillips (56) 2019

3.5cm (extensive) vs 2.5cm (limited) ATL resection	TLE Adults >18yrs 207 (4)	NS<br>RR 0.98<br>[0.83, 1.16]	†			-1<br>Despite the CI overlap, point estimates favour SAH, whether at 1 or 5 yrs or Engel I or IA.					+<br>West, Nevitt (3) 2019
Extended lesionectomy vs limited lesionectomy confined to cavernoma and hemosiderin	Cavernomas in adults and children 245 (7)	NS OR 0.96 [0.44, 2.08]	Removed 1 article with bias		-1<br>f/up 6 months						+<br>Shang-Guan, Wu (54) (2018)

Extensive resection of surrounding haemosiderin vs no excision of haemosiderin in cavernomas	Mainly Adults with cavernomas (but also few children)	OR 1.61 [1.10, 2.38]									+<br>Ruan, Yu (42)<br>2015
											++<br>West, Nevitt (3) 2019
Temporal Lobe (vs ET) resections<br><br>Lobe of resection<br><br>TL vs ET	Adults and Children with FCD<br><2384 (32, 2)<br><br>Tuberous Slceorisis in children <186 (<20, 1)<br><br>Repeat surgery in focal DRE 943 (12)	OR 1.35 [1.13, 1.61]<br>OR 1.92 [1.06, 3.45]<br><br>NS on PLS method<br><br>NS OR=1.5 [0.8, 3.0]	Trial sequence analysis, sensitivity analyses including removing individual studies.Subgroup analyses for geographical locations. Chen, Chen (58) (2019)<br><br>No clear trend in 1st and 2nd resection subgroups		-1<br>Engel II was considered seizure free Chen, Chen (58) (2019)		-1 No funnel plots/trim fill etc Rowland, Englot (29)<br>Begg rank correlation test and Egger linear regression test with trim and fill Chen, Chen (58)			+1<br>Permutation testing was performed\to evaluate the significance of the component, and bootstrapping was used to identify significant contributors to the component. PLS accounted for latent structure of data and ordinal Engel outcomes	++, +<br>Rowland, Englot (29) (2012), Chen, Chen (58) (2019)<br><br>+++<br>Ibrahim, Morgan (36) (2015)<br><br>++<br>Krucoff, Chan (50) (2017)

										classes. Ibrahim, Morgan (36)	
TL vs ET	Paediatrics ? (~23)	OR 2 [1.4, 2.9] direct and ~NMA p=0.025									+ Widjaja, Jain (60) 2020
ET vs hemispherectomy	Paediatrics ? (~23)	NS									+ Widjaja, Jain (60) 2020
surgical lobe	Paediatric ET (frontal, parietal, Rolandic, occipital, multilobed) 537 (26)	NS[u] See their table 1									+ Englot, Breshears (37) 2013
	Low grade gliomas in adults TL vs ET <2641 (<23)	NS NA									+ Shan, Fan (53) 2018

	Type of surgery (frontal, posterior and other) in ET non lesional children <95 (<17)	NS									+ Ansari, Maher (28) 2010
Lobe of surgery<br>TL reference had the highest compared to all other lobes, all significant (multilobar, parietal, occipital, frontal, hypothalamus)<br>>1000	Children and adults	Significant but no effect size given									+++ Lamberink, Otte (62)
Mesial vs lateral TL epileptic focus<br>(as determined on sEEG, subdural grid; or ATL/SAH vs neocortectomy)	MRI neg TLE 92 (8)	NS<br>OR 1.39 [0.61, 3.2]	NOS scores ranged from 4 to 6 stars								+ Wang, Zhang (44) (2016)
Complete excision (of lesion)	Adults and Children with FCD<br><2581 (31, 2)<br>Adults and Children with FLE 345 (7, 1)	OR 3.91 [3.03, 5.32] OR 12.5 [7.14, 20]<br>RR 1.99, [1.47, 2.84]	Trial sequence analysis, sensitivity analyses removing individual studies.Subgroup analyses for geographical locations. Chen, Chen (58)		-1<br>Engel II was considered seizure free Chen, Chen (58)		-1<br>No funnel plots/trim fill Rowland, Englot (29)<br>Begg rank correlation test and Egger linear regression test with trim and fill as necessary Chen, Chen (58)	+2 large OR<0.1 or OR >10			++, +++<br>Rowland, Englot (29) (2012), Chen, Chen (58) (2019)

	Repeat resective surgery for focal DRE 273 (11)	OR = 2.6, [1.3, 5.3]	-1 heterogenous categorisations								++<br>Englot, Wang (30) (2012)
							Funnel plots: undetected				+<br>Krucoff, Chan (50) (2017)
	Adults and children 2930 (39)	RR 1.41 [1.32, 1.50]	-1 †	-1<br>I^2 77.76% with p <0.0001; outcome subgroup differences also significant I^2 89.51% p<0.0001although the direction of effects are similar. Extratemporal subgroup ommited as only 1 study.							
	Adults and children TL subgroup 1266 (13)	TL RR 1.11 [1.03, 1.2]									+<br>West, Nevitt (3) 2019
	Paediatrics 893 (15)	OR 7.69<br>[4.76, 12.5]		consistent				+1 large OR >4			++<br>*Widjaja, Jain (60) 2020*
Gross total resection vs subtotal resection	Low grade gliomas in adults 1379 (16)	RR 1.47 [1.37, 1.59]									+<br>Shan, Fan (53) 2018

Side of resection (left vs right)	TL and ET Children and adults 2976 (41, 2)	NS OR 0.85 [0.54, 1.34], NS RR 1.04 [0.99, 1.1]	-1 †		-1<br>Engel outcomes in 22 studies and other definitions in 25		undetected				+, ++<br>Tonini, Beghi (24) (2004), West, Nevitt (3) 2019
	TLE adults ? (35)	NS Unweighted crude OR 0.57 [0.26, 1.24]									++<br>Willmann, Wennberg (27) (2007)
	ET, Adults, Non-lesional<br>131 (?, 1)	NS	-1<br>Small sample sizes form multiple centres, heterogenous outcome reporting								+<br>Ansari, Tubbs (18) (2010)
	Adults and children with FLE <1199 (<21, 1)	NS NA	-1<br>Limited info								+<br>Englot, Wang (30) (2012)
	Children with TLE 537 (15, 1)	NS[u]<br>OR[c] crude 1.07 [0.72, 1.60]									++<br>Englot, Rolston (32) (2013)
	MRI neg TLE 320 (15)	NS, slightly favours Left TL, OR 1.33 [0.84, 2.08]	NOS 4-6 stars Wang, Zhang (44) (2016)								+<br>Wang, Zhang (44) (2016)

Side of resection L v R	Repeat surgery for focal DRE 1st surgery 218 (?)<br><br>2nd/last surgery 209 (?)	NSu<br><br>Surgery #1 ORc* 0.73 [0.43, 1.25]<br><br>NSu<br><br>Surgery #2 ORc* 0.77 [0.44, 1.33]				-1 imprecise and effect sizes and CIs estimated. Krucoff, Chan (50)					+<br><br>Krucoff, Chan (50) (2017)
	Paediatric ET<br><br>326 (15)	NS<br><br>ORuc* 0.99 [0.64, 1.53]									+<br><br>Englot, Breshears (37) 2013
	Children and adults hemispherectomy 539 (29)	NS<br><br>OR 1.17, [0.79, 1.73], p = 0.43									++<br><br>Hu, Zhang (46) 2016
	ET non lesional children <95 (<17)	NSu									+<br><br>Ansari, Maher (28) 2010

Frontal, central, posterior vs other resections	ET, Adults, Non-lesional<br><br>81 (?, 1)	NS	-1<br><br>Small sample sizes form multiple centres, heterogenous outcome reporting								+<br><br>Ansari, Tubbs (18) (2010)
Geographical location of surgery N America vs elsewhere	Tuberous Sclerosis in children <186 (<20, 1)	NS on PLS								+1<br><br>Permutation testing was performed\to evaluate the significance of the component, and bootstrapping was used to identify significant contributors to the component.<br><br>PLS accounted for latent structure of data and ordinal Engel outcomes classes	+++<br><br>Ibrahim, Morgan (36) (2015)
Year of surgery >1000	Children and adults	NS									+++<br><br>Lamberink, Otte (62)

7. Pathology											
Presence of Tumours	TL and ET<br>Children and adults 3357 (54, 2)	OR 1.74 [1.25, 2.5]<br>RR 1.23 [1.14, 1.32]	-1 †	Q=19.3, p=0.08	-1<br>Engel outcomes in 22 studies and other definitions in 25						++<br>Tonini, Beghi (24) (2004)<br>++<br>West, Nevitt (3) 2019
low-grade epilepsy associated neuroepithelial tumour (LEAT)<br>majority were gangliogliomas and DNET	Children and adults 1325 (<37)	77·5% (SF)<br>No OR as used as baseline									+++<br>Lamberink, Otte (62)
Ganglioglioma 672		80·4%									
DNET 484		74·8%									
FCD Type II vs other FCD (Palmini Classification)	Adults and Children with FCD<br><2014 (17, 1)	OR 1.38 [1.22, 1.57]	Trial sequence analysis, sensitivity analyses including removing individual studies	I^2=14%, p=0.24<br>Not in subgroup analyses in asia (OR=1.24 []0.75, 2.04] Chen, Chen (58)	-1<br>Engel II was considered seizure free Chen, Chen (58)		-1<br>No funnel plots/trim fill Rowland, Englot (29)				++<br>Rowland, Englot (29) (2012)
FCD type 2 vs type 1 Palmini	Children and adults FCD 1580 (34)	OR 1.92 [1.54, 2.44]	Subgroup analyses for geographical locations. Chen, Chen (58)				Begg rank correlation test and Egger linear regression test with trim and fill as necessary Chen, Chen (58)				++<br>Chen, Chen (58) (2019)
FCD type IIb in network meta-analyses of subtypes (NMA)	Adults and children with FCD	OR 1.89 [1.01, 3.57]				-1 CI of OR approached 1					+<br>Chen, Chen (58) (2019)

“lesional”: tumours, CD, or other lesion (tuber, vascular malformation) i.e. positive pathology<br><br>vs<br><br>“non-lesional”: traumatic, infectious (Englot, Rolston (32) included HS in non-lesional)	FLE<br><br>Mixed Adults and Children 825 (16, 1)	RR 1.67, [1.36, 28.6]				1<br><br>Wide CI	Funnel plots: undetected (not shown in Englot, Wang (30)) Harward, Chen (49)				+<br><br>Englot, Wang (30) (2012)
	TL in Children 945 (29, 1)	OR 1.08, [1.02, 1.15]									+<br><br>Englot, Rolston (32)
	Repeat resective surgery for focal DRE 507 (12)	OR = 3.2, [1.9, 5.3]									++<br><br>Krucoff, Chan (50) (2017)
Focal pathological lesion	MRI neg TLE 167 (7)	NS (p=0.36) OR=1.36 [0.7, 2.63]	NOS 4-6 stars<br><br>Wang, Zhang (44)								+<br><br>Wang, Zhang (44) (2016)
	Occipital Lobe and posterior quadrant. Mixed adult and paediatric 167 (9)	OR 2.08 [1.58, 2.89]	-1 No statistical adjustments “impossible to perform a multivariate analysis looking for interactions across variables” e.g. didn’t adjust for lesions. Harward, Chen (49)								+<br><br>Harward, Chen (49) 2017

	Paediatric ET 695 (28)	OR 1.34, [1.19, 1.49									++<br>Englot, Breshears (37) 2013
Neuro-migrational defects	TL and ET<br>Children and adults ? (6, 1)	NS<br>OR 0.66 [0.42, 1.03]		Q=9.8, p=0.08	-1<br>Engel outcomes in 22 studies and other definitions in 25						+<br>Tonini, Beghi (24) (2004)
FCD vs gliosis	ET, Adults, Non-lesional<br>115 (?, 1)	NS	-1<br>Small sample sizes form multiple centres, heterogenous outcome reporting								+<br>Ansari, Tubbs (18) (2010)
Presence of FCD (vs absence)	Adults and children TLE and ET 3572 (46)	RR 0.90 [0.85, 0.95]	-1 †								++<br>West, Nevitt (3) 2019
Vascular malformation	Adults and Children 1488 (19)	NS pooled RR 1.07 [0.94, 1.21] adj for outcomes scale	- 1 †<br>Cavernomas not evaluated separately so uncertain of significance	No broad changes to result according to outcomes scales (Engel, Other or seizure freedom for 1 yr).	I^2= 0% for both overall heterogeneity and subgroup differences						++<br>West, Nevitt (3) 2019<br>++

Vascular malformation (cavernomas and others) vs low grade neuroepithelial<br>Cavernomas<br>Others	Children and Adults 443 (<37)<br>323<br>120	NS 74·0%<br>OR 0.79 [0·60 - 1·06]<br>77·1%<br>65·8%			Indirect evidence that vascular malformations are as prognostic as LEATs						Lamberink, Otte (62)
Tumour, HS > Rasmussen > MCD, TS > HH	Proportions only				-1<br>SF not clearly defined, some individual studies included Engel Classes I and II		Funnel plots and trim and fill test to impute bias effect estimates: difference between observed and imputed <10%<br>higher quality was associated with reduced SF percentages - although not statistically significant, the magnitude of effect was significant<br>(-0.31				+<br>*Widjaja, Jain (60)*<br>*2020*
Astro vs non astrocytoma	Low grade gliomas in adults<br><2641 (<23)	NS NA									+<br>Shan, Fan (53) 2018
hippocampal sclerosis 2948 vs Low grade neuroepithelial tumours	Children and adults<br>2948 (<37)	71·5%<br>OR 0.79 [0·65 - 0·89]									+++<br>Lamberink, Otte (62)

FCD type I or MCD<br>Vs LEAT<br>426	Children and adults	Negative<br>50·0%<br>OR 0.38 [0·28 - 0·49]									+++<br>Lamberink, Otte (62)
Other MCD<br>(Hypothalamic hamartomas, tubers and others)<br>405	Children and adults	Negative<br>52·3%<br>OR 0.44 [0·29 - 0·63]									++<br>Lamberink, Otte (62)
No histopathological lesion (comprised of gliosis and normal tissue)<br>740	Children and adults	Negative<br>53·5%<br>OR 0.36 [0·30 - 0·46]									+++<br>Lamberink, Otte (62)
FCD type II<br>796	Children and adults	64·9%<br>NS<br>OR 0.8 [0·61 - 1·09]			Indirectly supports type II as + prognostic feature, as not significantly worse than LEAT						++<br>Lamberink, Otte (62)
Encephalitis<br>(Rasmussen's and limbic, herpes, neurocysticercosis)<br>124<br><br>Encephalitis - Rasmussen's subgroup<br>72	Children and adults	59·7%<br>OR 0.43 [0·22 - 0·73]<br><br>72·2%									++<br>Lamberink, Otte (62)

Glial scar<br>261	Children and adults	59·4%<br>OR 0.53 [0·39 - 0·70]									+++<br>Lamberink, Otte (62)
Non-LEAT (astrocytoma, oligodendroglioma, cysts, ependymoma, meningioma, neurocytoma, and pleomorphic xanthoastrocytoma)<br>310	Children and adults	68·4%<br>NS<br>OR 0.75 [0·54 - 1·02]									++<br>Lamberink, Otte (62)

[1]**Study limitations (risk of bias or internal validity):** Differential surveillance for outcome between studies, failure of accurate measurement of all known prognostic factors and to match for prognostic factors and/or lack of adjustment in statistical analysis. Includes selective reporting bias. [12 14]

[2]**Inconsistency of results:** If some studies suggest substantial prognostic value using relative measures while others suggest no effect or negative prognostic value then it may be appropriate to rate down for quality. Criteria for evaluating consistency include similarity of point estimates, extent of overlap of confidence intervals, and statistical criteria including tests of heterogeneity and I^2. If inconsistent results cannot be explained by differences in subgroups (populations undergoing surgery, surgical intervention, or outcome definitions and follow-up), then the quality of the body of evidence is rated down. [15]

[3]**Indirectness of evidence:** If there are differences in the populations, interventions and/or outcomes being studied compared to what we are interested in, or if interventions are compared without direct head-to-head comparisons, studies can be rated down [16]. We only rate down if there is a compelling reason to believe the populations studied differ from the population of interest that the magnitude of effect would differ significantly e.g. because of the presumed differences in the maturing brain, paediatric and adult epilepsy surgery populations should be investigated separately or as subgroups to avoid reduced population applicability. We also rate down if there is an outcomes discrepancy, whereby the seizure-freedom duration of follow-up is less than that of interest (at least 12months) e.g. in the inclusion criteria. We consider ILAE 1 and 2 seizure free, which is equivalent to Engel Ia/Ib and thus also rate down inclusion criteria that include undifferentiated Engel I.

[4]**Imprecision:** e.g. for a single meta-analysis, effect sizes which overlap the neutral point (for RR and OR, 1) suggesting the feature is not prognostic, but the boundaries of the confidence interval are skewed significantly in one direction such that the largest plausible effect is that the feature is either positively or negatively correlated with outcomes. [11]

[5]**Publication bias:** clinical features that are non-prognostic and smaller effect sizes are less likely to be published and these can be assessed by funnel plots. Cumulative iterative meta-analyses could be indirectly inferred from the publication dates to ascertain time-lag bias. Risk of publication bias is probably larger for small, observational, and industry-funded studies. [14]

[6]**Rating up:** relative risks above 2 (below 0.5) are rated up one level, and above 5 (below 0.2) are rate up two levels unless the CI overlaps significantly with these thresholds. If the baseline proportion of outcomes is low, odds ratios are treated similarly, otherwise a higher threshold is used. Studies were also rated up if a dose response was present or if all plausible residual confounders or biases would reduce a demonstrated effect, or suggest a spurious effect when results show no effect [17]

TL(E): Temporal Lobe (Epilepsy). ETE: Extratemporal lobe Epilepsy. APOS: Acute Postoperative Seizures. NS: Not Significant. CI: confidence aura. HS: Hippocampal Sclerosis. MCD: Malformations of Cortical Development. FCD: Focal Cortical Dysplasia. HH: Hypothalamic Hamartoma. TS: Tuberous Sclerosis. OR: Odds Ratio. RR: Relative Risk Ratio. SF: Seizure Freedom. NMA: Network Meta-Analysis. ATL: Anterior Temporal Lobectomy. SAH: Selective Amygdalohippocampectomy. NA: Not Available. HS: Hippocampal Sclerosis. ECoG: Electrocorticography. PLS: partial least squares. NOS: Newcastle-Ottawa Scale.

*: Our calculated CI from their data. [C]: Effect size derived from article data. [u]: Univariate analyses. [m]: Multivariate analyses. †A weakness of the Cochrane review is that they “did not class any of the pre-operative prognostic factors of interest…as confounders” so in general our GRADE score is one lower.

2.3 Supplementary Table 3: Essential Prognostic Features for Epilepsy Surgery (EPF)

EPF		Prognostic Value and Supporting Evidence Base						
**Feature**	**Population(s) or Subgroup(s)**	**Range of Effect Sizes for Seizure-Freedom**	**Comments**	**Units of Analysis (Individual Patients*)**	**Individual Studies***	**Meta-Analytical References**	**Publication Year of meta-analysis (first, last)**	**GRADE score**
**1. Clinical Features**								
***Severe* developmental delay/learning disability and IQ ≤75**	≥16 yrs TLE<br><br>≥16 yrs TLE non-HS structural lesions<br><br>Children TS<br><br>Children and Adults with hemispherectomy	RR 0.66 [0.54, 0.94]<br><br>RR 0.26 [0.14, 0.50]<br><br>OR 0.14 [0.04, 0.48]<br><br>OR 0.61 [0.46, 0.82]	Five meta-analyses evaluated developmental delay and learning disability as a negative prognostic factor, three of which were significant,[20, 35, 46] while two others were not significant for "*moderate* to severe developmental delay" in paediatric Rasmussen's,[56] *undifferentiated* "mental retardation" in tuberous sclerosis[33] or *continuous* pre-operative IQ scores.[35] The presence of moderate developmental delay in the dichotomised category and continuous IQ scores (on average only 2.3 lower in not seizure-free group, p<0.009)[20] may have masked the subgroup significance for IQ<75.	2256	54	Chelune, Naugle (20) 1998<br><br>Fallah, Guyatt (35) 2013<br><br>Hu, Zhang (46) 2016	1998 – 2019	++<br>Low<br><br>Favours absence of severe learning disability
**Febrile Convulsions (FC)**	TL and ET in both Children and Adults	OR 2.08 [1.2, 3.7]<br>RR 1.09 [1.01, 1.17]	>1 yr SF subgroup had good outcomes with febrile seizures but the other subgroups did not (subgroup I^2 = 49%, p 0.14).[3]<br>Non-Engel outcomes in more than half of individual studies.[24] It is expected for FC to be favourable in TLE and unfavourable in ET.	4879	20	Tonini, Beghi (24) 2004,<br><br>West, Nevitt (3) 2019	2004 – 2019	+<br>Very Low<br><br>Favours presence of FC

**Postsurgical:**<br><br>**Without Acute Postoperative Seizures (APOS) within 30 days**	Children and Adults, TLE and ET<br><br>Paediatric subgroup	Overall OR 4.2 [2.97, 5.93]<br><br>OR 5.71 [3.32, 9.8]	There was a rather large effect: without APOS 73.5% seizure-free, vs with APOS 39%. Results of subgroup analyses were similar. A metaregression showed earlier onset of APOS within 24hrs was NS 1.87 [0.89, 3.95]. Note however, like most meta-analyses, although over 64% had presurgical lesions, no adjustment was made. Although they reported no significant bias, we note asymmetry in overall and paediatric APOS funnel plots (their Fig 1e).<br><br>The clinical significance of the absence APOS is questionable, as it is both postsurgical, and it is logically expected that seizure-free patients would be a subset of those without APOS.	1983	17	Giridharan, Horn (45) 2016	2016	++<br>Low<br><br>Favours absence of APOS
**2. Imaging Features**								
**Mesial Temporal Sclerosis (MTS) or Hippocampal Sclerosis (HS)**	Adults and Children with TLE	OR 2.13 [1.57, 2.86]<br>RR 1.17 [1.12, 1.23]	12 out of 15 individual studies from Tonini, Beghi (24) had point estimates favouring HS; 5 of the 15 were prospective and a further sixth study was combined retrospective and prospective in design.<br><br>Amongst those with MTS, 74% were seizure free, compared to 62% of those without MTS. This included patients with MTS on imaging or pathology.[3]	4430	61	Tonini, Beghi (24) 2004<br><br>West, Nevitt (3) 2019	2004 – 2019	++<br>Low<br><br>Favours presence of HS
**Abnormal or Lesional MRI**	Adults and Children with TLE and ET<br><br><br><br>TL subgroups<br><br>ET subgroups<br><br>Adults and Children with FCD<br><br>Adults and Children with FLE	OR 2.27 [1.54, 3.45]<br>OR 2.5 [2.1, 3.0]<br>OR 2.03 [1.67, 2.47]<br>RR 1.28 [1.20, 1.37]<br><br>OR 2.7 [2.1, 3.5]<br>OR 1.76 [1.34, 2.32]<br><br>OR 2.9 [1.6, 5.1]<br>OR 2.88 [1.53, 5.43]<br><br>OR 1.67 [1.33, 2.16]<br><br>RR 1.64 [1.32, 2.08]	An odds ratio of 2.5 for abnormal MRI in a meta-analysis from 2010 of any population translates to a relative risk of RR 1.4,[5] which is comparable to the RR effect size of 1.28 from the 2019 Cochrane review and a RR of 1.64 in patients with FLE.[3, 30]<br><br>Non lesional cases were significantly more frequent in ET (45%) than in TL (24%),[5] however, when funnel plots and trim and fill were performed, either no publication bias was observed,[29, 31] or the difference between observed and imputed results varied by under 10% (for the paediatric population).[60]	18076	193	Tonini, Beghi (24) 2004<br><br>Téllez-Zenteno, Ronquillo (5) 2010<br><br>Yin, Kang (31) 2013<br><br>West, Nevitt (3) 2019	2004 – 2020	++<br>Low<br><br>Favours abnormal MRI, see comments on two borderline meta-analyses.

	Children with TLE<br><br>Adults and Children with Occipital Lobe and Posterior Quadrant Epilepsy<br><br>Children<br><br>Children with hemispherectomy	OR 1.27 [1.16, 1.40]<br><br>OR 3.24 [2.03, 6.55]<br><br>OR 1.85 [1.14, 2.94]<br><br>OR 4.6 [1.27, 16.62]	The only groups for which meta-analyses seemed to be non-significant were in 196 patients with repeat surgery from 7 studies done in 2017, and 506 children with extratemporal resections from 23 studies performed in 2013. Yet in both of these, the effect sizes and skewed confidence intervals still favoured abnormal MRI with odds ratios of 1.9 [0.6, 5.4] and an unweighted OR of 1.44 respectively.[37, 50]			Rowland, Englot (29) 2012<br><br>Englot, Wang (30) 2012<br><br>Englot, Rolston (32) 2013<br><br>Harward, Chen (49) 2017<br><br>Widjaja, Jain (60) 2020<br><br>Cao, Liu (43) 2016		
**SPECT: Subtraction Ictal and Inter-ictal SPECT co-registered to MRI (SISCOM)**	TL and ET 275 (11)<br><br>ET subgroup 209 (11)	OR 3.28 [1.90, 5.67]<br><br>OR 2.44 [1.34, 4.43]	Odds ratios were favourable for SISCOM abnormalities, for both TL and ET patients across 11 studies. Overall heterogeneity was non-significant (I2 = 16.6%, p=0.29)	275	11	Chen and Guo (47) 2016	2016	++<br>Low<br><br>Favours SPECT-SISCOM abnormality
**3. Neurophysiological Features**								
**Focal Ictal or Interictal or Invasive EEG**<br><br>Interictal EEG localised to temporal lobe<br><br>Localised/unifocal ictal (scalp) EEG	Repeat resective surgery for focal DRE (n=192)<br><br>MRI neg TLE (n=149)[44]<br><br>Tuberous Sclerosis in Children<br><br>MRI neg TLE (n=125)[44]	OR 3.6 [1.6, 8.2]<br><br>OR 3.38 [1.57, 7.25]<br><br>OR 3.21 [1.35, 7.58] Positive prognostic value on PLS also.<br><br>OR 3.89 [1.66, 9.08]	Favours focal EEG changes at any point and via any method with comparable odds ratios between 3 and 4 (lower in paediatric extratemporal epilepsy, OR<1.93).<br><br>The two earliest meta-analyses from 2012 and 2013 – in children and adults with FLE, and children with TLE – did not find unifocal ictal scalp EEG to be significant for seizure-freedom.[30, 32] The former's GRADE score was "very low" while the latter's was "low" with 445 participants across 14 studies and OR[c] 1.23 [0.73, 2.06]. These discrepancies bring the overall quality of evidence down to very weak.	878	54	Krucoff, Chan (50) 2017<br><br>Wang, Zhang (44) 2016<br><br>Fallah, Guyatt (35) 2013<br><br>Ibrahim, Morgan (36) 2015<br><br>Englot, Breshears (37) 2013	2013 – 2017<br><br>Notable exceptions from 2012-2013[30, 32]	+<br>Very Low<br><br>Favours focal EEG changes, with notable inconsistency

Ictal EEG localised to temporal lobe	Children ET	OR 1.55 [1.24, 1.93]	Other meta-analyses with combined unusual dichotomised features (unifocal interictal scalp EEG abnormality **or** no interictal abnormality at all) were non-significant on both weighted OR 1.54 [0.73, 3.26] and partial least squares.[35, 36]					
**4. Multimodal Concordance**								
**EEG-MRI Concordance**<br><br><br><br><br><br><br>**Ipsilateral**	TL and ET<br>Children and adults<br><br>Children with Tuberous Sclerosis<br><br>Children and adults hemispherectomy	OR 2.36 [1.07, 5.26][24]<br>RR 1.25 [1.15, 1.37][3]<br><br>OR 4.9 [1.8–13.5]<br>Positive prognostic value on PLS<br><br>OR 2.17 [1.30, 3.7]	One of the highest qualities of evidence ratings and most consistent results was for EEG and MRI concordance. However, concordance between other modalities, such as semiology and neurophysiology or imaging have not been investigated in meta-analyses. Note that the largest effect size (OR 4.9) and widest confidence interval belongs to the earliest meta-analysis (2013) with the fewest number of patients.[35]<br><br>For full limitations and GRADE scores and comments for individual meta-analysis see Supplementary Table 2.	2296	55	Tonini, Beghi (24) 2004<br><br>West, Nevitt (3) 2019<br><br>Fallah, Guyatt (35) 2013<br><br>Ibrahim, Morgan (36) 2015<br><br>Hu, Zhang (46) 2016	2013 – 2019	+++<br>Moderate<br><br>Favours EEG and MRI concordance
**5. Genetics: none**								
**6. Surgical Technique or Anatomic Features**								
**Temporal Lobe (vs ET) resections**	Adults and children with FCD<br><br>Repeat surgery in focal DRE (n=943)<br><br>Children<br><br>Low grade gliomas in adults (n<2641)	OR 1.35 [1.13, 1.61]<br>OR 1.92 [1.06, 3.45]<br><br>NS OR 1.5 [0.8, 3.0]<br><br>OR 2 [1.4, 2.9]<br><br>NS NA	It is well established from numerous individual studies from many centres that surgery for TLE carries the best prognosis. This is only true as far as the diagnosis is correct, and like other surgical features, is more about patient selection and diagnosis than which lobe is resected. Nevertheless, meta-analyses have confirmed that surgery for TLE carries a favourable prognosis.[29, 58, 60]	15012	127	Rowland, Englot (29) 2012<br><br>Chen, Chen (58) 2019<br><br>Krucoff, Chan (50) 2017	2012 – 2020	+<br>Very Low<br><br>Favours surgery for TLE

			This only applies to TLE, and meta-analyses investigating other lobes or ET vs hemispherectomy did not find significant results.[36, 60 28, 37]<br><br>Nevertheless, TLE is **not** associated with better outcomes in low grade gliomas in adults, nor in cases where the first surgery for presumed TLE fails, i.e. the second surgery does not carry better prognosis (although the point estimate confirms this trend), presumably due to diagnosis and patient selection rather than the lobe of resection per se.[50]			Widjaja, Jain (60) 2020<br><br>Shan, Fan (53) 2018<br><br>Lamberink, Otte (62) 2020		
**Complete Excision**<br><br>Gross total resection vs subtotal resection	Adults and children with FCD (n<2581)<br><br>Adults and children with FLE (n=345)<br><br>Repeat resective surgery for focal DRE (n=273)<br><br>Adults and children (n=2930)[3]<br><br>Adults and children TL subgroup[3] (n=1266)<br><br>Children (n=893)<br><br>Low grade gliomas in adults (n=1379)	OR 3.91 [3.03, 5.32] OR 12.5 [7.14, 20]<br><br>RR 1.99 [1.47, 2.84]<br><br>OR 2.6 [1.3, 5.3]<br><br>RR 1.41 [1.32, 1.50]<br><br>TL subgroup RR 1.11 [1.03, 1.2]<br><br>OR 7.69 [4.76, 12.5]<br><br>RR 1.47 [1.37, 1.59]	Complete excision of lesions or structural abnormalities are unanimously associated with better outcomes in both adults and children, irrespective of the nature of the lesion, across 7 meta-analyses. The definition of complete excision wasn't always specified, but is usually interpreted through imaging and histology.<br>The largest effect sizes (OR 12.5 and 7.69) belong to two of the latest published in 2019 and 2020, the former also used trial sequential and sensitivity analyses.[58, 60] Trial sequential analysis can prevent over 90% of false positive results in conventional meta-analyses.[64] However, Engel II was also considered SF.[52]	8401	119	Rowland, Englot (29) 2012<br><br>Chen, Chen (58) 2019<br><br>Englot, Wang (30) 2012<br><br>Krucoff, Chan (50) 2017<br><br>West, Nevitt (3) 2019<br><br>Widjaja, Jain (60) 2020<br><br>Shan, Fan (53) 2018	2012 – 2020	+++<br>Moderate<br><br>Favours complete excision

7. Pathological Features

**Presence of Tumours**<br><br>Low-grade epilepsy associated neuroepithelial tumours (LEAT) vs HS<br><br>LEAT vs FCD type I or MCD<br><br>LEAT vs Hypothalamic hamartomas, tubers and other MCD<br><br>LEAT vs Encephalitis<br><br>Vs Glial scars	Children and Adults TLE and ET<br>| | Children and adults (mainly gangliogliomas and DNET)<br>| | | | | | | | | |	OR 1.74 [1.25, 2.5]<br>RR 1.23 [1.14, 1.32]<br>OR 2.78 [2.17, 3.33]<br><br>OR 1.27 [1.12, 1.54]<br><br>OR 2.63 [2.04, 3.57]<br><br>OR 2.27 [1.59, 3.45]<br><br>OR 0.43 [0·22 - 0·73]<br><br>OR 0.53 [0·39 - 0·70]	Epilepsy surgery for tumours has good outcomes, compared to non-tumour causes, or even compared to HS or multiple other pathologies but not compared to glial scars and encephalitis.[62]	8261	91	Tonini, Beghi (24) 2004<br><br>West, Nevitt (3) 2019<br><br>Lamberink, Otte (62) 2020	2004 – 2020	+++<br>Moderate<br><br>Favours Tumours
**FCD**<br><br>Presence of FCD (vs absence)<br><br>Type II vs Other<br><br>Type II vs Type I<br><br>Type IIb in Network Meta-Analysis of Subtypes	Adults and children TLE and ET (n=3572)<br><br>Adults and Children with FCD (n<2014)<br><br>Children and adults FCD (n=1580)[58]<br><br>Adults and children with FCD[58]	RR 0.90 [0.85, 0.95]<br><br>OR 1.38 [1.22, 1.57]<br><br>OR 1.92 [1.54, 2.44]<br><br>OR 1.89 [1.01, 3.57]	FCD Type III was developed by ILAE in 2011, and one study investigated pre-2011 Palmini Classification.[29]<br><br>Overall there has been better outcomes in the absence of FCD (vs presence) amongst 3572 adults and children in 2019 in the Cochrane review,[3] while in 2010 there was no significant difference between FCD vs gliosis amongst 115 extratemporal adults.[18] When FCD is present, two reports have shown direct evidence that Type II has better prognosis than other subtypes,[29, 58] and another shows indirect evidence as type I is associated with worse outcomes compared to neuroepithelial tumours, whereas type II is not. [62]	Presence or absence of FCD<br>3572<br><br>FCD subtypes<br>3594	Presence or absence of FCD<br>46<br><br>FCD subtypes<br>51	Rowland, Englot (29) 2012<br><br>Chen, Chen (58) 2019<br><br>West, Nevitt (3) 2019<br><br>Lamberink, Otte (62) 2020	2012 – 2019	++<br>Low<br><br>Favours the absence of FCD, otherwise favours FCD type II(b)

**Lesional Pathology vs Non-Lesional**	FLE<br>Mixed Adults and Children (n=825)<br><br>TL in Children (n=945)<br><br>Repeat resective surgery for focal DRE (n=507)<br><br>MRI neg TLE (n=167)<br><br>Occipital Lobe and posterior quadrant. Mixed adult and paediatric (n=167)<br><br>Paediatric ET (n=695)	RR 1.67 [1.36, 28.6]<br><br>OR 1.08 [1.02, 1.15]<br><br>OR 3.2 [1.9, 5.3]<br><br>NS (p=0.36)<br>OR 1.36 [0.7, 2.63]<br><br>OR 2.08 [1.58, 2.89]<br><br>OR 1.34 [1.19, 1.49]	Lesional was defined as positive or focal pathology e.g., tumours, MCD, tubers or vascular malformation vs non-lesional which included traumatic and infectious causes (Englot, Rolston (32) included hippocampal sclerosis in non-lesional). Despite the heterogeneity, all studies except for one favoured positive pathology, and this study was in MRI negative TLE.[44] This suggests the causal prognostic pathway of imaging abnormality and presence of pathological abnormality are shared, and adjusting for one may render the other non-prognostic.<br><br>Within lesion on pathology in children, tumours and hippocampal sclerosis predict better outcomes than Rasmussen's, which in turn has better postsurgical prognosis than malformations of cortical development and tuberous sclerosis, followed by hypothalamic hamartomas.[60]	3306	101	Englot, Wang (30) 2012<br><br>Englot, Rolston (32) 2013<br><br>Krucoff, Chan (50) 2017<br><br>Wang, Zhang (44) 2016<br><br>Harward, Chen (49) 2017<br><br>Englot, Breshears (37) 2013<br><br>Widjaja, Jain (60) 2020	2012 – 2017	++<br>Low<br><br>Favours presence of focal pathological lesion

Supplementary Table 3: The essential prognostic features (EPF). OR/RR=Odds Ratios and Relative Risks over 1 indicate better outcomes. *=upper bound of estimate. NS=Not-significant. [c]=calculated (usually unweighted) effect size. MCD=malformations of cortical development. [u]=univariate. [m]=multivariate. TL=Temporal Lobe. ET=Extratemporal. FLE=Frontal Lobe Epilepsy. PLS=Projection to Latent Space.

2.4 Supplementary Table 4: Uncertain Prognostic Features (UPF)

UPF		Mixed Results Evidence Base						
**Feature**	**Population(s) or Subgroup(s)**	**Range of Effect Sizes for Seizure-Freedom**	**Comments**	**Individual Patients***	**Individual Studies***	**Meta-Analytical References**	**Publication Year (first, last)**	**GRADE score**
**1. Clinical Features**								
**History of Head Injury**	Adults and Children	NS<br>RR 0.99 [0.86, 1.13]	Although there was no overall effect, subgroup analyses by outcome were very different and inconsistent.[3]	551	7	West, Nevitt (3) 2019	2019	+<br>Very Low<br><br>Unclear
**CNS Infections**	TL and ET in Children and Adults	NS<br>OR 0.73 [0.29, 1.82]	Non-Engel outcomes in more than half of individual studies.[24]	<<3511	2	Tonini, Beghi (24) 2004	2004	+<br>Very Low<br><br>Unclear

**Focal (partial) Seizure Semiology<br>vs<br>Generalised**	ET non-lesional Adults, Adults and Children with FCD<br><br>Adults and Children with FLE<br><br>TL in Children<br><br>TS<br><br>TS in Children<br><br>Repeat Surgery<br><br>Children with Rasmussen's<br><br>ET in Children<br><br>Adults and Children with hemispherectomy<br><br>Adults with supratentorial low grade gliomas<br><br>Children with hemispherectomy	NS<br>OR 1.46<br><br>NS (p=0.05)<br><br>OR 1.36 [1.20, 1.56]<br><br>NS OR 1.15 [0.42, 3.11]<br><br>OR = 3.1 [1.2, 8.2]<br><br>NS<br><br>NS HR 0.8 [0.43, 1.51]<br><br>OR 1.61 [1.18, 2.35]<br><br>OR 1.84, [1.18, 2.89]<br><br>RR 0.76 [0.67, 0.85]<br><br>NS	When significant, focal seizures were always a positive predictor of seizure-freedom, except for one study on gliomas in which even TL surgery wasn't associated with better outcomes.[53]<br><br>One group used two separate techniques in two different studies for the same set of patients. In bivariate logistic regression, OR was 3.1;[35] whereas using partial least squares, the effect was still positive but the bootstrapped CI just crossed point of non-significance; instead, focal ictal EEG was significant on PLS,[36] suggesting a correlation between focal seizure semiology and focal ictal EEG as if observing the same factor from different points in their causal pathway.<br><br>Those with less than ~250 individual participants were less likely to show significance.<br>On balance, likely to be a positive prognostic feature with low power and in need of adjusting for other confounders.	Total = 4965<br><br>Significant studies = 4025<br><br>NS studies = 940	Total = 124<br><br>Significant = 70<br><br>NS = 54	Ansari, Tubbs (18) 2010<br><br>Rowland, Englot (29) 2012<br><br>Englot, Wang (30) 2012<br><br>Englot, Rolston (32) 2013<br><br>Zhang, Hu (33) 2013<br><br>Fallah, Guyatt (35) 2013<br><br>Ibrahim, Morgan (36) 2015<br><br>Krucoff, Chan (50) 2017<br><br>Harris, Phillips (56) 2019<br><br>Englot, Breshears (37) 2013<br><br>Hu, Zhang (46) 2016<br><br>Shan, Fan (53) 2018<br><br>Cao, Liu (43) 2016	2010 – 2019	+<br>Very Low<br><br>Possible positive prognostic feature, given the right patient selection and circumstances

**Epileptic (Infantile) Spasms**	TS<br><br>TS in Children	OR 0.45 [0.24, 0.85]<br><br>NS OR 0.84 [0.35, 2.03]<br>NS on PLS	Data for TS patients only.<br><br>Heterogenous definitions of seizure freedom without sensitivity analyses and no funnel plots to investigate publication bias.[33]<br>Small samples (median 7, IQR [3,25]), did not assess heterogeneity nor bias.[35]<br>One meta-analysis had a moderate GRADE score for spasms.[36]	343	27	Zhang, Hu (33) 2013<br><br>Fallah, Guyatt (35) 2013<br><br>Ibrahim, Morgan (36) 2015	2013 – 2015	+<br>Very Low<br><br>Unclear
**Low Seizure Frequency or Without daily seizures**	Adults and Children with FLE<br><br>Paediatric ET<br><br>TL in Children	NS<br><br><br>NS<br><br>unclear	Although two meta-analyses found seizure frequency to be non-significant, another in TLE in Children included 103 patients from 5 individual studies and the unweighted effect size seemed promising OR[c] 2.98 [1.24, 7.16],[32] but no further attempt at inverse variance weighting was performed and it is unclear if this would have remained significant.	1357	25	Englot, Wang (30) 2012<br><br>Englot, Breshears (37) 2013	2012 – 2013	+<br>Very Low<br><br>Unclear

**Age at Seizure Onset**<br><br>Dichotomised <1yr<br><br>Log10 age at onset<br><br>Dichotomised <18yrs<br><br>Younger age at onset<br><br>Meta Regression	Adults with non-lesional ET<br><br>TS<br><br>TS in Children<br><br>MRI negative TLE<br><br>Children with Rasmussen's<br><br>Children and children with TLE subgroup<br><br>Children with non lesional ET<br><br>Children with hemispherectomy	NS<br><br>OR 0.47 [0.24, 0.92]<br><br>NS OR 1.52 [0.77, 2.99]; also NS on PLS<br><br>NS OR 1.09 [0.38, 3.07]<br><br>HR^{u} 0.91 [0.85, 0.96] NS HR^{m} [0.87, 1.04]<br><br>overall: $OR^{c} = e^{0.346} = 1.41$ (p<0.001)<br>TLE: $OR^{c} = e^{0.144} = 1.15$ (p=0.023)<br><br>NS<br><br>SMD = 0.26, [0.03, 0.49] P = 0.028	Age at onset was higher in seizure free patients in a metaregression of children with and without TLE but not ET, hemispherectomy, tumours or MCD.[60] Age over 1 year was also associated with better outcomes in TS.[33]<br>However, it was non-significant in at least 5 other meta-analyses.<br><br>One study had a significant HR of 0.91 (favouring younger age at onset) on univariate, but NS on multivariate testing adjusted for the length of follow-up[56].<br><br>Even when results were significant, they weren't too dissimilar e.g., seizure onset age tended to be younger by about 5 months in the Engel Class II to IV group compared with the Engel Class I group (median 3.6 months vs 8.4 months, p=0.006).[43]	1252	115	Ansari, Tubbs (18) 2010<br><br>Zhang, Hu (33) 2013<br><br>Fallah, Guyatt (35) 2013<br><br>Ibrahim, Morgan (36) 2015<br><br>Wang, Zhang (44) 2016<br><br>Harris, Phillips (56) 2019<br><br>Widjaja, Jain (60) 2020<br><br>Ansari, Maher (28) 2010<br><br>Cao, Liu (43) 2016	2010 – 2020	+<br>Very Low<br><br>Unclear
**Age at Surgery**<br><br><18 yrs at surgery<br><br><18 yrs at surgery<br><br>Continuous<br><br>Log base 10 or <5yrs<br><br><18 yrs at surgery<br><br>Metaregression of mean age and outcomes<br><br>Age < 18 yrs	Adults non lesional ET<br><br>Adults and Children FCD<br><br>Adults and Children FLE<br><br>Children with TLE<br><br>TS<br><br>MRI negative TLE<br><br>Children and Adults TLE/ET<br><br>Occipital Lobe and Posterior Adults and Children	NS (ANOVA)<br><br>NS<br><br>NS<br><br>NS (t-test)<br><br>NS<br><br>NS OR 1.09 [0.38, 3.07]<br><br>NS<br><br>OR 1.54 [1.13, 2.18]	Many results from multiple meta-analyses are non-significant.<br><br>The only significant results were on unadjusted univariate hazard ratios for children with Rasmussen's,[56] metaregression on paediatric epilepsies where younger age at surgery was associated with better outcomes overall and for TL and ET subgroups (but not for hemispherectomy, tumours or MCD)[60], and older age at surgery for low grade gliomas in adults with a cut-off value of 45 yrs.[53]<br><br>Two studies looked at hemispherectomy / Rasmussen's in	10798	221	Ansari, Tubbs (18) 2010<br><br>Rowland, Englot (29) 2012<br><br>Englot, Wang (30) 2012<br><br>Englot, Rolston (32) 2013<br><br>Zhang, Hu (33) 2013	2010 – 2020	++<br>Low<br><br>Probably not prognostic

Metaregression<br><br>Age > 45 yrs<br><br>Dichotomised age >18 yrs	Repeat Surgery in focal DRE<br>first or last surgery<br><br>Children Rasmussen's<br><br>Children overall<br>TL<br>ET<br><br>Children ET<br><br>Low grade gliomas in Adults<br><br>Children non lesional ET<br><br>Children hemispherectomy<br><br>Cavernomas Adults and Children	NS<br><br>HR 0.93[u] [0.89, 0.97]<br>NS[m] HR 0.95[m] [0.90, 1.0]<br><br>Overall: $OR^{c} = e^{-0.189} = 0.83$ (p<0.001)<br>TL: $OR^{c} = e^{-0.093} = 0.91$ (p=0.031)<br>ET: $OR^{c} = e^{-0.173} = 0.84$ (p 0.004)<br><br>NS<br><br>RR 1.12 [1.01, 1.23]<br><br>NS<br><br>NS 0.95 [0.38, 2.37]	children, one was NS[43] and the other had a significant HR of 0.93 on univariate, but NS on multivariate testing adjusted for the length of follow-up[56]; a third study was NS in the hemispherectomy subgroup.[60]<br><br>Most other studies were variations on adults or children, TL or ET, lesional or non-lesional and all were non-significant. Two studies looked at TS, although both NS, both also very weak on quality of evidence rating.[33, 35]<br><br>On balance, it is probable that the lack of adjusting for known prognostic factors and heterogeneous follow-up times has resulted in falsely significant results.			Fallah, Guyatt (35) 2013<br><br>Ibrahim, Morgan (36) 2015<br><br>Wang, Zhang (44) 2016<br><br>Giridharan, Horn (45) 2016<br><br>Harward, Chen (49) 2017<br><br>Krucoff, Chan (50) 2017<br><br>Harris, Phillips (56) 2019<br><br>Widjaja, Jain (60) 2020<br><br>Englot, Breshears (37) 2013<br><br>Shan, Fan (53) 2018<br><br>Ansari, Maher (28) 2010<br><br>Cao, Liu (43) 2016<br><br>Shang-Guan, Wu (54) 2018		

**Duration of Epilepsy Prior to Surgery**	Adults non-lesional ET	NS	Upper bound estimates for participants were nearly half-and-half split between significant and non-significant meta-analyses. All significant studies favoured shorter duration.<br><br>Studies that favoured shorter duration had relative risk point estimates between 1.2 – 1.32 and odds ratios between 1.52 – 2.57 which are compatible as odds ratios tend to overestimate. Although one study showed possible increases in the effect sizes when longer durations of 10 and 20 years until surgery were considered,[57] another subgroup metaregression was non-significant.[45]<br><br>Both age at onset and age at surgery from Harris, Phillips (56) lost their significance when adjusted for outcome follow up variability, however, they did not seem to report the same multivariate result for duration of epilepsy. Given the univariate HR approaches 0.97 in children with Rasmussen's, this feature would, we suspect, also become non-significant on adjusting and we therefore include the patients and individual studies in the non-significant category.[56]<br><br>There were no clear patterns to the populations or ages studied, and no clear adjustments or interactions with TL resections made. Given TL connectivity and involvement in propagation, interaction between duration of epilepsy in TL and ET would be useful. On balance, there may be better prognosis with shorter duration	Overall<br>18645<br><br>NS<br>5786<br><br>Favours Shorter Duration<br>12859<br><br>Favours Longer Duration<br>0	Overall<br>185<br><br>NS<br>98<br><br>Favours Shorter Duration<br>87<br><br>Favours Longer Duration<br>0	Ansari, Tubbs (18) 2010<br><br>Englot, Wang (30) 2012<br><br>Englot, Rolston (32) 2013<br><br>Wang, Zhang (44) 2016<br><br>Giridharan, Horn (45) 2016<br><br>Krucoff, Chan (50) 2017<br><br>Krucoff, Chan (50) 2017<br><br>Harris, Phillips (56) 2019<br><br>Bjellvi, Olsson (57) 2019<br><br>Englot, Breshears (37) 2013<br><br>Shan, Fan (53) 2018<br><br>Lamberink, Otte (62) 2020<br><br>Ansari, Maher (28) 2010<br><br>Cao, Liu (43) 2016<br><br>Shang-Guan, Wu (54) 2018	2010 – 2020	+<br>Very Low<br><br>Likely favours shorter duration
	Adults and Children with FLE	NS						
Mean duration	TLE in Children	NS (t-test)						
Shorter duration	MRI neg TLE	OR 2.57 [1.21, 5.47]						
Duration or time between surgeries	Repeat surgery for focal DRE	NS (t-test)						
Shorter duration	Paediatric Rasmussen's	HR 0.92^{u} [0.88, 0.97]						
<2 vs >2yrs	Children and adults all lobes	RR 1.20 [1.05, 1.39]						
<5 vs >5yrs		RR 1.24 [1.08, 1.42]						
<10 vs >10yrs		RR 1.25 [1.09, 1.43]						
<20 vs <20yrs		RR 1.33 [1.08; 1.65]						
<5 vs >10yrs		RR 1.32 [1.19; 1.46]						
Subgroup Metaregression		NS						
≤ 7 years	Paediatric ET	OR 1.52 [1.07, 2.14]						
Duration ≥ 1 year	Adults with low grade gliomas	RR 0.82 [0.75, 0.91]						
	Children and Adults with low-grade epilepsy associated neuroepithelial tumour	0.97 [0·96 – 0·99]						
	ET nonlesional children	NS						
	Children hemispherectomy	NS						
	Cavernomas adults and children	NS						

			of epilepsy but this is confounded by selection bias of clearer diagnoses of focal epileptogenic zones.					
**Postsurgical:**<br><br>**Postoperative Semiology Different to Presurgical Semiology**	Adults and Children, TL and ET	NS 4.24 [0.93, 19.25]	Although results suggest when semiology changes postoperatively there is a higher chance of seizure freedom, this depends on the definition of "seizure-freedom" and with 109 participants across only 3 studies, was not statistically significant.	109	3	Giridharan, Horn (45) 2016	2016	+<br>Very Low<br><br>Unclear
**2. Imaging Features**								
**FDG-PET Focal Interictal Hypometabolism**	Adults with TLE and ET<br><br>Adults TLE? (35, 1)<br><br>Adults and Children with FLE<br><br>MRI negative TLE	NS<br><br>NS<br><br>NS<br><br>NS<br>OR 2.11 [0.95, 4.65]	PET does not appear to add value in patients localized by ictal scalp EEG and/or MRI. One meta-analysis looked at MRI negative TLE, in which 127 patients across 5 studies was NS, however, the OR was 2.11 and the confidence interval was skewed (p=0.06), favouring a positive prognostic effect.[44]	1479	107	Willmann, Wennberg (27) 2007<br><br>Englot, Wang (30) 2012<br><br>Wang, Zhang (44) 2016	2007 – 2016	+<br>Very Low<br><br>PET may have prognostic value for MRI negative TLE
**3. Neurophysiological Features**								
**Postoperative interictal discharges**	TL and ET<br>Children and adults<br><br>TLE subgroup	OR 0.28 [0.08, 0.95]<br>NS Adjusted for outcomes RR 0.91 [0.68, 1.22]<br><br>RR 0.81 [0.70, 0.94]	Although we would expect postoperative discharges to be correlated with seizures and poor outcomes, and this is reflected in the overall OR of 0.28, RR <1, and TLE subgroup effect size, when adjusted for outcomes, the overall effect was not statistically significant.[3] 2019	1547	9	Tonini, Beghi (24) 2004<br><br>West, Nevitt (3) 2019	2004 – 2019	+<br>Very Low<br><br>Probably favours lack of postoperative discharges in at least TLE

**Preoperative intracranial (invasive) EEG Monitoring**<br><br>**(EcoG, electrico-corticography, includes subdural grids and stereoEEG – see below for comparison between invasive methods)**	TL and ET<br>Children and adults 1547 (27, 2)<br><br>ET, Adults, Non-lesional<br><br>Children and adults with FLE<br><br>Tuberous Sclerosis<br><br>Children with TLE<br><br>Repeat surgery on focal DRE<br><br>Paediatric ET<br><br>ET nonlesional children <95 (<17)	OR 0.37 [0.22, 0.63], RR 0.85 [0.78, 0.93]<br><br>NS<br><br>NS<br><br>NS<br>OR 1.6 [0.76, 3.37]<br><br>NS OR[c] crude 1.31 [0.84, 2.04]<br><br>OR = 0.4, [0.2, 0.9]<br><br>NS<br>OR[c] 0.77 [0.50, 1.19]<br><br>NS	Of the 9 meta-analyses investigating the presence or absence of invasive monitoring, only 2 were of ++ "low" quality on the GRADE score, compared with 7 with + "very low" rating. Both of the higher rated meta-analyses found that performing intracranial EEG was associated with worse outcomes with a relative risk for seizure freedom of 0.85 and odds ratios of 0.4.[3, 50] This may be expected due to selection bias of the most difficult cases. The Cochrane review included the largest number of participants at 1547 across 21 individual studies.[3]<br><br>The other studies suffered from one or more limitations, including no funnel plots to investigate publication bias,[33] having small sample sizes from multiple centres with heterogenous outcome reporting, [18 24] providing limited information on the actual effect size, [30] and most did not adjust for other variables which could also affect the power to detect significance.	4198	105	Tonini, Beghi (24) 2004<br><br>West, Nevitt (3) 2019<br><br>Ansari, Tubbs (18) 2010<br><br>Englot, Wang (30) 2012<br><br>Zhang, Hu (33) (2013)<br><br>Englot, Rolston (32) 2013<br><br>Krucoff, Chan (50) 2017<br><br>Englot, Breshears (37) 2013<br><br>Ansari, Maher (28) 2010	2004 – 2019	+<br>Very Low<br><br>Most likely favours lack of invasive monitoring
**sEEG vs Subdural Grid**	TL and ET adults and children (but not from children only studies)<br><br>Nonlesional (n=237)<br><br>Lesional (n=665)<br><br>TL (n=470)<br><br>ET (n=420)<br><br>Any	Overall RR = 64.7% [59.2, 69.8] / 55.9% [50.9, 60.8] = 1.16[c]<br><br>NS RR = 52% / 54.4% = 0.96<br><br>RR = 71.6% / 57.3% = 1.25<br><br>RR = 73.9% / 56.7% = 1.30<br><br>RR = 61% / 46.7% = 1.31<br><br>SDE 64.3% [61.1, 67.5]<br>SEEG 54% [50.8, 57.3]<br>OR[cu] 0.65 [0.45, 0.95]<br>p=0.025[cu]	While there were significant differences favouring sEEG overall (p = 0.02), in lesional (p = 0.031), and temporal subgroups (p = 0.002), the average follow-up for sEEG was 10 months while for subdural grids was nearly 19months but no adjustment was made for duration of follow up. Furthermore, while there wasn't significant heterogeneity in the sEEG studies (I^2 = 11.86%; p = 0.318), there was in the subdural group (I^2 = 54.47%; p = 0.002)<br><br>Funnel plots and Egger's tests resulted in no overall or subgroup changes.	2461	64	Toth, Papp (59) 2019<br><br>[63] 2020	2019, 2020	+<br>Very Low<br><br>Note that this is a complex feature, likely confounded by many others, and interactions with other clinical features have not been investigated.<br><br>Possibly favours sEEG overall and specifically in

		there was no difference in seizure freedom rates regardless of resection (p = 0.0565)	On balance, although the subdural grid cases were more likely to progress to surgery in both meta analyses, likely to due to more straightforward cases, it's possible that the results favour sEEG specifically for lesional cases when there is considerable uncertainty about the epileptogenic zone localisation.					lesional cases, but uncertain.
**Interictal Spikes (presence of)**	TL and ET<br>Children and adults	NS<br>OR 1.82 [0.86, 3.88]	The evidence base of this feature is uncertain, although the point estimate supports a positive prognostic feature with a somewhat skewed confidence interval, this is not statistically significant. Tonini, Beghi (24) investigated 3511 patients across 47 studies for 13 features, interictal spikes comprised only 3 individual studies and the exact number of cases was not presented but proportionally would be on the order of ~224.	<<3511	3	Tonini, Beghi (24) 2004	2004	+
**Lateralised (Unilateral) Interictal EEG**<br><br>Unilateral vs bilateral interictal spikes	Children and adults with FLE<br><br>Tuberous Sclerosis (n=127)<br><br>Adults and children (n=1414)<br><br>Paediatric ET (n=130)<br><br>Adults and children hemispherectomy (n=413)	NS<br><br>OR 2.42 [1.11, 5.27]<br><br>RR 1.14 [1.05, 1.24],<br><br>NS<br>OR[c] 2.22 [0.98, 5.05]<br><br>OR 1.66, [1.03, 2.67]	Two meta-analyses were non-significant, one without further data and the other raw data presented showing a calculated OR of 2.22.[30, 37] Note the lack of TLE subgroup. The largest was the Cochrane review with 1414 patients with RR 1.14 for unilateral vs bilateral interictal spikes. Let's *assume* 70% seizure-freedom for unilateral spikes, if a presurgical patient's interictal EEG shows bilateral spikes – everything else being equal – we should reduce this expectation from 70% down by a factor of 1/1.14 i.e., 61%. (NNT ~11–25 depending on definition of seizure freedom).	3283	62	Englot, Wang (30) 2012<br><br>Zhang, Hu (33) 2013<br><br>West, Nevitt (3) 2019<br><br>Englot, Breshears (37) 2013<br><br>Hu, Zhang (46) 2016	2012 – 2019	+<br>Very Low<br><br>Likely favours unilateral interictal EEG

**Unilateral vs Bilateral Ictal EEG (Lateralized Ictal EEG)**	Adults and Children with FCD<br><br>Tuberous Sclerosis (n=159)<br><br>Adults and Children hemispherectomy (n=414)	NS<br>OR 1.03 [0.82, 1.31]<br><br>OR 2.48 [1.17, 5.24]<br><br>OR 1.88 [1.15, 3.07]	Two meta-analyses estimated odds ratios around 2 for better seizure free outcomes for lateralised ictal EEG in TS and hemispherectomy at any age.Zhang, Hu (33), Hu, Zhang (46) Another found no significance in FCD.Rowland, Englot (29)<br>As with all other features, it must be remembered that even if EEG lateralises, there are other factors such as correct localisation and complete resection of the epileptogenic zone. Therefore, on balance, probably supports favourable outcomes in unilateral ictal EEG abnormalities.	2587	25	Rowland, Englot (29) 2012<br><br>Zhang, Hu (33) 2013<br><br>Hu, Zhang (46) 2016	2012-2016	+<br>Very Low<br><br>Probably favours unilateral ictal EEG
**4. Multimodal Concordance: None**								
**5. Genetics: None**								
**6. Surgical Technique or Anatomic Features**								
**Extensive Surgical Resection**<br><br>Extensive frontal vs localised<br><br>Lobectomy (extensive) vs tuberectomy<br><br>ATL (extensive) vs SAH (selective)<br><br>ATL vs SAH<br><br>ATL vs SAH	TL and ET Children and adults (n<3511)<br><br>FLE, adults and children (n=651)<br><br>Tuberous sclerosis (n=189)<br><br>TLE children and adults (n=1203)<br><br>TLE and HS subgroup (n=1092)<br><br>TLE mainly adults (n=?)<br><br>TLE (n=626)<br><br>SAH vs ATL in TLE (n=1397)	OR 4.27 [2.06, 8.85]<br><br>RR 0.58 [0.41, 0.79]<br><br>OR 1.96 [1.01, 3.7]<br><br>RR 1.32 [1.12, 1.57]<br><br>RR 1.26 [1.05, 1.51]<br><br>NS OR 1.14 [0.93, 1.39] p=0.201<br><br>NS<br>RR 1.01 [0.54, 1.09]<br><br>Overall OR 0.65 [0.51, 0.82]	As a supercategory comprising of ATL (vs SAH), lobectomy or hemispherectomy (vs lesionectomy), or extended lesionectomy vs limited resections, it isn't clear whether extensive surgical resection results in better SF.<br><br>Meta-analyses supporting extensive resections include those for all patients, [24] tuberous sclerosis,[33] TLE[34 40], and paediatric Rasmussen's [56].<br>If no significant study favoured limited resections, this would not have been unexpected, given at one end of the extreme spectrum, total brain removal might be expected to result in SF. However, extensive frontal lobe resections resulted in worse outcomes compared to limited resections. [30] This was the only result which favoured limited resections.<br>If taken at face value, non-inferiority or worse SF outcomes for selective procedures, except for frontal lobe	9494	120	Tonini, Beghi (24) 2004<br><br>Englot, Wang (30) 2012<br><br>Zhang, Hu (33) 2013<br><br>Josephson, Dykeman (34) 2013<br><br>Jain, Tomlinson (52) 2018<br><br>Kuang, Yang (38) 2013<br><br>Hu, Zhang (40) 2013	2004 – 2019	

Lesionectomy vs multilobar resection surgery)<br><br>Hemispherectomy (vs resective)<br><br>3.5cm (extensive) vs 2.5cm (limited) ATL resection<br><br>Extended vs limited lesionectomy	Tuberous sclerosis children (n=186)<br><br>Rasmussen's Paediatric (n<187)<br><br>TLE Adults >18yrs (n=207)<br><br>Cavernomas in adults and children (n=245)<br><br>Extensive resection of surrounding haemosiderin vs resection of cavernoma only	NS on PLS<br><br>HR 0.28^{u} [0.18, 0.45]<br>HR 0.30^{m} [0.18, 0.49]<br><br>NS RR 0.98 [0.83, 1.16]<br><br>NS OR 0.96 [0.44, 2.08]<br><br>OR 1.61 [1.10, 2.38]	patients, may suggest a role for healthy frontal cortex in seizure inhibition (and conversely indicate the role of TL for seizure propagation), or suggest selection bias where large resections are made when there is less clear localisation, the former which warrants further investigation if the effect persists after adjustment.<br><br>On balance, ATL results in better SF, but has other detrimental outcomes including on cognition, more cortical thinning than SAH especially in the frontal and insular cortices ["Remote effects of epilepsy surgery: long-term morphological changes after surgical resection" Poster, AES 2020]<br>Even for ATL, some studies supported better outcomes, [34 40] whilst other did not. [52 38] There was no clear time or quality of study trend, nor by technique (trans-sylvian, transcortical or subtemporal).			Ibrahim, Morgan (36) 2015<br><br>Harris, Phillips (56) 2019<br><br>West, Nevitt (3) 2019<br><br>Shang-Guan, Wu (54) 2018<br><br>Ruan, Yu (41) 2015		
**7. Pathological Features**								
**Vascular Malformations**	Adults and Children	NS RR 1.07 [0.94, 1.21]<br>NS OR 0.79 [0·60 - 1·06]	Vascular malformations are non-prognostic when adjusted for different outcomes scales, on pathology or imaging.[24] As a group, they are also not statistically significant when compared to low grade neuroepithelial tumours, and this provides indirect evidence that vascular malformations may be prognostic. Cavernomas especially, are likely prognostic (77.1% Engel I) compared to others (65.8%).[62]	1931	56	West, Nevitt (3) 2019<br><br>Lamberink, Otte (62) 2020	2019 – 2020	++<br>Low<br><br>Not Prognostic

Supplementary Table 4: Features with inconclusive or conflicting prognostic value for epilepsy surgery. *=upper bound of estimate, not including subgroup analyses. NS=Not-significant. ^{c}=calculated (unweighted) effect size. MCD=malformations of cortical development. ^{u}=univariate. ^{m}=multivariate. TL=Temporal Lobe. ET=Extratemporal. FLE=Frontal Lobe Epilepsy. PLS=Projection to Latent Space.[36]

2.5 Supplementary Table 5: Non-Prognostic Features (NPF)

NPF Features		Non-Prognostic Evidence Base						
Feature	Population(s) or Subgroup(s)	Range of Effect Sizes for Seizure-Freedom	Comments	Individual Patients*	Individual Studies*	Meta-Analytical References	Publication Years of meta-analyses (first, last)	GRADE score
**1. Clinical Features**								
**Sex: male vs female**	Adults and Children with FLE<br><br>Children with TLE, Tuberous Sclerosis, MRI neg TLE, Repeat surgery in focal DRE, Paediatric ET<br><br>Children and adults hemispherectomy<br><br>Low grade gliomas in adults<br><br>Children with hemispherectomy	All NS	All were non-significant, a large proportion even on weighted univariate tests which otherwise tend to overestimate significance.<br><br>Individual unweighted effect sizes ranged from OR 0.83 [0.42, 1.64][c] in repeat surgery for focal DRE[50] to OR 1.44 [0.86, 2.41] in MRI negative TLE.[44]	5974	148	Englot, Wang (30) 2012<br><br>Englot, Rolston (32) 2013<br><br>Zhang, Hu (33) 2013<br><br>Fallah, Guyatt (35) 2013<br><br>Ibrahim, Morgan (36) 2015<br><br>Wang, Zhang (44) 2016<br><br>Krucoff, Chan (50) 2017<br><br>Englot, Breshears (37) 2013<br><br>Hu, Zhang (46) 2016<br><br>Shan, Fan (53) 2018	2012 – 2018	+++<br>Moderate<br><br>Non-Prognostic

						Cao, Liu (43) 2016		
**Epilepsia Partialis Continua (EPC)**	Children undergoing hemispherectomies	NS	Not significant on unweighted univariate testing which is more likely to make a statistical type I error. Although result is from 1 meta-analysis, the population required to have sufficient numbers with EPC is unlikely to be found in vast numbers elsewhere.	127	7	Cao, Liu (43) (2016)	2016	++<br>Low<br><br>Non-Prognostic
**2. Imaging Features**								
**Number of Cortical Tubers**<br><br>**≤ 4 vs > 4 tubers**<br><br>**"Less tuber burden"**	Tuberous Sclerosis	NS<br>OR 1.12 [0.49, 2.57]<br><br>NS<br>OR 1.01 [0.96, 1.07]<br>Also NS on PLS.	Note that the patients from two of these meta-analyses were the same patients, albeit in one they were reported to be 186 and in another 181, the main difference was in the methodology, where the 2015 paper used PLS. Overall there were few sample sizes, no adjustment except in PLS, and they either did not perform funnel plots,[33, 35] or did not assess heterogeneity,[35] or used heterogenous seizure-freedom definitions without sensitivity analyses.Zhang, Hu (33)	286	24	Zhang, Hu (33) 2013<br><br>Fallah, Guyatt (35) 2013<br><br>Ibrahim, Morgan (36) 2015	2013 – 2015	++<br>Low<br><br>Non-Prognostic
**Magnetic ^{1}H Spectroscopy Abnormality:**<br><br>**Ipsilateral to Resected Lobe**	TLE adults and children<br><br>TLE, adults and children, normal MRI	OR 4.9 [1.97, 12.17]<br><br>NS	There was a single meta-analysis that we found and it showed that there was no more value to spectroscopy compared to conventional MRI for patients 3 to 66 years of age. "Fifteen centers performed chemical shift imaging and seven centers used single-voxel spectroscopy. Most studies were obtained at 1.5 T"	121	22	Willmann, Wennberg (26) 2006	2006	+<br>Very Low<br><br>Probably no more valuable than conventional MRI abnormality
**Encephalomalacia**	Adults and children	NS<br>RR 0.78 [0.52, 1.17]	Encephalomalacia was NS in the Cochrane meta-analysis, it was also not significant on subgroup analyses.[3]	317	5	West, Nevitt (3) 2019	2019	+<br>Very Low<br><br>Not Prognostic
**Enhancement, oedema, and/or mass effect**	Low grade gliomas in adults	NS	These combined features are not clinically prognostic of low-grade glioma resection for seizure freedom. Although NS, the point estimate and confidence interval are unavailable.	2641	23	Shan, Fan (53) 2018	2018	+<br>Very Low<br><br>Not Prognostic

**Vascular Lesions**	Adults and Children TL and ET	NS<br>OR 0.66 [0.30, 1.46]	Only 1 meta-analysis investigated this in 2004, comprising only 3 individual studies, its pathological counterpart was also NS.[3]	<<3511	3	Tonini, Beghi (24) 2004	2004	+<br>Very Low<br><br>Not Prognostic
**3. Neurophysiological Features**								
**Intraoperative Invasive EEG (EcoG, electrico-corticography)**	Children and adults with FLE	NS p=0.14<br>OR[c] 1.23 [0.95, 1.62]		1024	21	Englot, Wang (30) 2012	2012	+++<br>Moderate<br><br>NotPrognostic
**Video Telemetry and Long Term Monitoring**	Children and adults with FLE<br><br>Lesional and non-lesional TLE and ET	NS (chi-squared)<br><br>All confidence intervals overlap with 1, with only a trend for OR>1 in lesional TLE subgroup (confounder)	Limited information on effect size provided.<br><br>Lesional TLE cases do well, and this was the only subgroup in which long term monitoring had a point effect size estimate greater than 1.	<<1199<br><br>539	<21<br><br>44	Englot, Wang (30) 2012<br><br>Kobulashvili, Kuchukhidze (55) 2018	2012, 2018	+<br>Very Low<br><br>Not Prognostic
**4. Multimodal Concordance: None**								
**5. Genetics: None**								
**6. Surgical Technique or Anatomic Features**								
**Mesial vs Lateral TL focus**	MRI neg TLE 92	NS<br>OR 1.39 [0.61, 3.2]	Mesial or lateral TLE, as determined by sEEG, subdural grids, or ATL/SAH vs neocortectomy, are not significant.	92	8	Wang, Zhang (44) 2016	2016	+<br>Very Low<br><br>Not Prognostic

**Side of Resection (Left vs Right)**	TL and ET Children and adults<br><br>TLE adults<br><br>ET, Adults, Non-lesional<br><br>Adults and children with FLE<br><br>Children with TLE<br><br>MRI neg TLE<br><br>Repeat surgery for focal DRE Surgery #1<br><br>Surgery #2<br><br>Paediatric ET<br><br>Children and adults hemispherectomy<br><br>ET non lesional children	NS<br>OR 0.85 [0.54, 1.34]<br>RR 1.04 [0.99, 1.1]<br><br>NS[u] OR 0.57 [0.26, 1.24]<br><br>NS<br><br>NS<br><br>NS[u]<br>OR[c] 1.07 [0.72, 1.60]<br><br>NS<br>OR 1.33 [0.84, 2.08]<br><br>NS[u]<br>OR[c] 0.73 [0.43, 1.25]<br><br>NS[u]<br>OR[c] 0.77 [0.44, 1.33]<br><br>NS<br>OR[uc] 0.99 [0.64, 1.53]<br><br>NS<br>OR 1.17, [0.79, 1.73], p = 0.43<br><br>NS[u]	Although there are some methodological issues which result in the quality of evidence GRADE score for side of resection for each meta-analysis not exceeding ++ "low", such as heterogenous outcome reporting, 11 meta-analysis spanning 15 years were unanimous in not finding significance in side of resection, given a low prior for theoretically considering that there may be better outcomes base on left or right sided surgery, this feature is unlikely to be prognostic irrespective of how many further analyses investigate it.	6550	188	Tonini, Beghi (24) 2004<br><br>West, Nevitt (3) 2019<br><br>Willmann, Wennberg (27) 2007<br><br>Ansari, Tubbs (18) 2010<br><br>Englot, Wang (30) 2012<br><br>Englot, Rolston (32) 2013<br><br>Wang, Zhang (44) 2016<br><br>Krucoff, Chan (50) 2017<br><br>Englot, Breshears (37) 2013<br><br>Hu, Zhang (46) 2016<br><br>Ansari, Maher (28) 2010	2004 – 2019	+++<br>Moderate<br><br>Not Prognostic
**Frontal, Central, or Posterior Resections vs Other**	ET, Adults, Non-lesional	NS		81	?	Ansari, Tubbs (18) 2010	2010	+<br>Very Low<br><br>Not Prognostic
**Geographical Location of Surgery: N America vs Elsewhere**	Tuberous Sclerosis in Children	NS on PLS	Only one meta-analysis, and so the GRADE score reflects the quality of the investigated feature from this meta-analysis alone.	186	20	Ibrahim, Morgan (36) 2015	2015	+++<br>Moderate<br><br>Not Prognostic

7. Pathological Features								
**Neuro-migrational defects**	TL and ET<br>Children and adults	NS<br>OR 0.66 [0.42, 1.03]	There was a trend whereby neuromigrational deficits were negative prognostic factors, but the number of participants in this analysis is unclear.	? (<<3511)	6	Tonini, Beghi (24) 2004	2004	+<br>Very Low<br><br>Not Prognostic
**Astrocytoma vs non-astrocytoma**	Low grade gliomas in adults	NS NA	The exact numbers of patients were not provided for this particular analysis.	<2641	<23	Shan, Fan (53) 2018	2018	+<br>Very Low<br><br>Not-Prognostic

Supplementary Table 5: Non-prognostic features. *=upper bound of estimate, not including subgroup analyses. NS=Not-significant. ^{c}=calculated (usually unweighted) effect size. MCD=malformations of cortical development. ^{u}=univariate. ^{m}=multivariate. TL=Temporal Lobe. ET=Extratemporal. FLE=Frontal Lobe Epilepsy. PLS=Projection to Latent Space.

2.6 Structural Causal Models

The attached text file, "SCM dagitty v5 super simplified" generates the simplified SCM when used on http://www.dagitty.net/dags.html (Supplementary Fig. 1, colour coded).

Similarly, "SCM dagitty v4" generates the complete SCM with all of the prognostic, non-prognostic and uncertain features and their relationships (Supplementary Fig. 2, colour coded).

The R codes can also be obtained from dagitty after pasting the contents of the text files. The output of dagitty also states which relationships are direct or biases, and when a specific model is specified, which variables are independent and no adjustment would be necessary.

2.6.1 A Simplified SCM

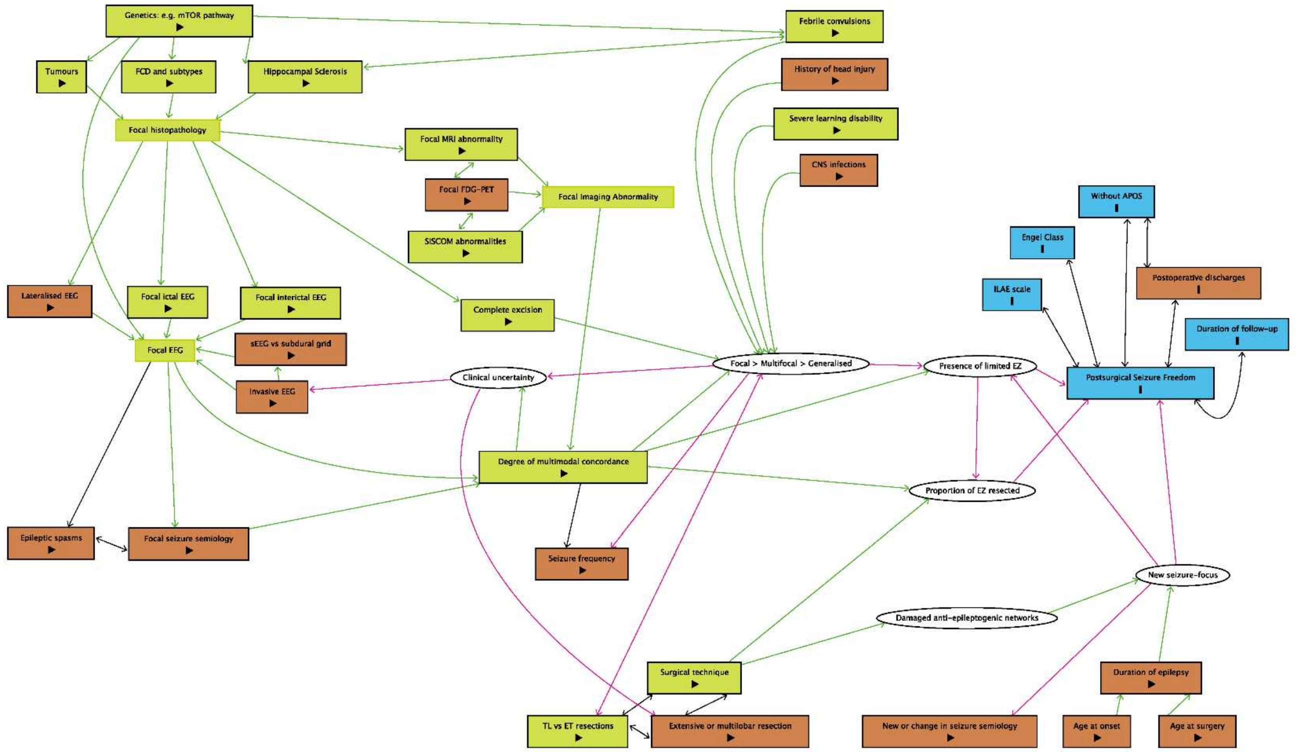

Supplementary Figure 1: Simplified structural causal model outline for adjusting between variables. In blue are the outcome variables. Latent variables are in oval shapes. Essential prognostic factors are in green and uncertain prognostic factors are in orange. Non-prognostic factors have been omitted for simplicity. Green arrows are causal paths, red arrows are biasing paths. Image created by authors using dagitty v3 [(http://www.dagitty.net/dags.html)](http://www.dagitty.net/dags.html) and can be recreated by pasting the text of supplementary file "SCM dagitty v5 super simplified" onto this website.

2.6.2 A More Complete SCM

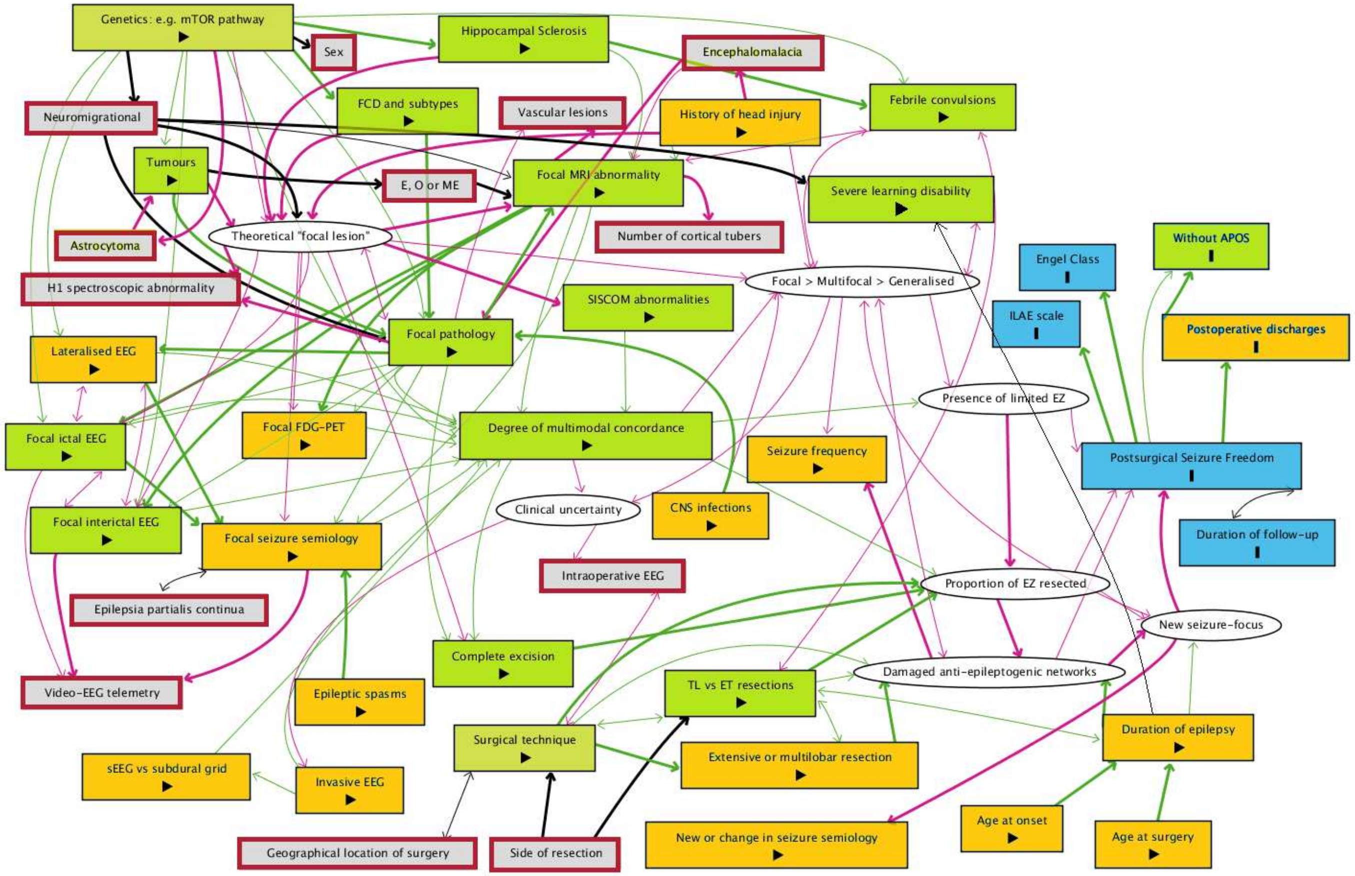

Supplementary Figure 2: A more complete structural causal model outline for adjusting between variables. In blue are the outcome variables. Latent variables are in oval shapes. Essential prognostic factors are in green, uncertain prognostic factors in amber, and non-prognostic factors have red borders. Green arrows are causal paths, red arrows are biasing paths. Image created by authors using dagitty v3 [(http://www.dagitty.net/dags.html)](http://www.dagitty.net/dags.html) and can be recreated by pasting the text of supplementary file "SCM dagitty v4" onto this website.

Supplementary References

1. Nevitt SJ, Staba R, Gloss D. The role of high-frequency oscillations in epilepsy surgery planning. The Cochrane Database of Systematic Reviews. 2017;2017(10).
2. West S, Nolan SJ, Cotton J, Gandhi S, Weston J, Sudan A, et al. Surgery for epilepsy. Cochrane Database Syst Rev. 2015(7):Cd010541.
3. West S, Nevitt SJ, Cotton J, Gandhi S, Weston J, Sudan A, et al. Surgery for epilepsy. Cochrane Database of Systematic Reviews. 2019(6).
4. Téllez-Zenteno JF, Dhar R, Wiebe S. Long-term seizure outcomes following epilepsy surgery: a systematic review and meta-analysis. Brain. 2005;128(5):1188-98.
5. Téllez-Zenteno JF, Ronquillo LH, Moien-Afshari F, Wiebe S. Surgical outcomes in lesional and non-lesional epilepsy: a systematic review and meta-analysis. Epilepsy research. 2010;89(2-3):310-8.
6. Höller Y, Kutil R, Klaffenböck L, Thomschewski A, Höller PM, Bathke AC, et al. High-frequency oscillations in epilepsy and surgical outcome. A meta-analysis. Frontiers in human neuroscience. 2015;9:574.
7. Stevelink R, Sanders MW, Tuinman MP, Brilstra EH, Koeleman BP, Jansen FE, et al. Epilepsy surgery for patients with genetic refractory epilepsy: a systematic review. Epileptic Disorders. 2018;20(2):99-115.
8. Pellino G, Gencarelli J, Bertelli S, Russo A, Fiumana E, Faggioli R. Epilepsy in isolated parenchymal neurocutaneous melanosis: A systematic review. Epilepsy & Behavior. 2020;107:107061.
9. Pilipović-Dragović S, Ristić AJ, Bukumirić Z, Trajković G, Sokić D. Long-term seizure outcome following epilepsy surgery in the parietal lobe: a meta-analysis. Epileptic Disorders. 2018;20(2):116-22.
10. Nakagawa S, Noble DW, Senior AM, Lagisz M. Meta-evaluation of meta-analysis: ten appraisal questions for biologists. BMC biology. 2017;15(1):1-14.
11. Guyatt GH, Oxman AD, Kunz R, Brozek J, Alonso-Coello P, Rind D, et al. GRADE guidelines 6. Rating the quality of evidence—imprecision. Journal of clinical epidemiology. 2011;64(12):1283-93.
12. Guyatt GH, Oxman AD, Vist G, Kunz R, Brozek J, Alonso-Coello P, et al. GRADE guidelines: 4. Rating the quality of evidence—study limitations (risk of bias). Journal of clinical epidemiology. 2011;64(4):407-15.
13. Balshem H, Helfand M, Schünemann HJ, Oxman AD, Kunz R, Brozek J, et al. GRADE guidelines: 3. Rating the quality of evidence. Journal of clinical epidemiology. 2011;64(4):401-6.
14. Guyatt GH, Oxman AD, Montori V, Vist G, Kunz R, Brozek J, et al. GRADE guidelines: 5. Rating the quality of evidence—publication bias. Journal of clinical epidemiology. 2011;64(12):1277-82.
15. Guyatt GH, Oxman AD, Kunz R, Woodcock J, Brozek J, Helfand M, et al. GRADE guidelines: 7. Rating the quality of evidence—inconsistency. Journal of clinical epidemiology. 2011;64(12):1294-302.
16. Guyatt GH, Oxman AD, Kunz R, Woodcock J, Brozek J, Helfand M, et al. GRADE guidelines: 8. Rating the quality of evidence—indirectness. Journal of clinical epidemiology. 2011;64(12):1303-10.
17. Guyatt GH, Oxman AD, Sultan S, Glasziou P, Akl EA, Alonso-Coello P, et al. GRADE guidelines: 9. Rating up the quality of evidence. Journal of clinical epidemiology. 2011;64(12):1311-6.
18. Ansari SF, Tubbs RS, Terry CL, Cohen-Gadol AA. Surgery for extratemporal nonlesional epilepsy in adults: an outcome meta-analysis. Acta Neurochir (Wien). 2010;152(8):1299-305.
19. Chandra PS, Vaghania G, Bal CS, Tripathi M, Kuruwale N, Arora A, et al. Role of concordance between ictal-subtracted SPECT and PET in predicting long-term outcomes after epilepsy surgery. Epilepsy Res. 2014;108(10):1782-9.
20. Chelune GJ, Naugle RI, Hermann BP, Barr WB, Trenerry MR, Loring DW, et al. Does presurgical IQ predict seizure outcome after temporal lobectomy? Evidence from the Bozeman Epilepsy Consortium. Epilepsia. 1998;39(3):314-8.
21. Altman DG. Practical statistics for medical research: CRC press; 1990.

22. Textor J, van der Zander B, Gilthorpe MS, Liśkiewicz M, Ellison GT. Robust causal inference using directed acyclic graphs: the R package ‘dagitty’. International journal of epidemiology. 2016;45(6):1887-94.
23. Devous MD, Sr., Thisted RA, Morgan GF, Leroy RF, Rowe CC. SPECT brain imaging in epilepsy: a meta-analysis. J Nucl Med. 1998;39(2):285-93.
24. Tonini C, Beghi E, Berg AT, Bogliun G, Giordano L, Newton RW, et al. Predictors of epilepsy surgery outcome: a meta-analysis. Epilepsy research. 2004;62(1):75-87.
25. Téllez-Zenteno JF, Hernández Ronquillo L, Moien-Afshari F, Wiebe S. Surgical outcomes in lesional and non-lesional epilepsy: a systematic review and meta-analysis. Epilepsy Res. 2010;89(2-3):310-8.
26. Willmann O, Wennberg R, May T, Woermann F, Pohlmann-Eden B. The role of 1H magnetic resonance spectroscopy in pre-operative evaluation for epilepsy surgery: a meta-analysis. Epilepsy research. 2006;71(2-3):149-58.
27. Willmann O, Wennberg R, May T, Woermann F, Pohlmann-Eden B. The contribution of 18F-FDG PET in preoperative epilepsy surgery evaluation for patients with temporal lobe epilepsy: a meta-analysis. Seizure. 2007;16(6):509-20.
28. Ansari SF, Maher CO, Tubbs RS, Terry CL, Cohen-Gadol AA. Surgery for extratemporal nonlesional epilepsy in children: a meta-analysis. Childs Nerv Syst. 2010;26(7):945-51.
29. Rowland NC, Englot DJ, Cage TA, Sughrue ME, Barbaro NM, Chang EF. A meta-analysis of predictors of seizure freedom in the surgical management of focal cortical dysplasia. Journal of neurosurgery. 2012;116(5):1035-41.
30. Englot DJ, Wang DD, Rolston JD, Shih TT, Chang EF. Rates and predictors of long-term seizure freedom after frontal lobe epilepsy surgery: a systematic review and meta-analysis. Journal of neurosurgery. 2012;116(5):1042-8.
31. Yin Z-r, Kang H-c, Wu W, Wang M, Zhu S-q. Do neuroimaging results impact prognosis of epilepsy surgery? A meta-analysis. Journal of Huazhong University of Science and Technology [Medical Sciences]. 2013;33(2):159-65.
32. Englot DJ, Rolston JD, Wang DD, Sun PP, Chang EF, Auguste KI. Seizure outcomes after temporal lobectomy in pediatric patients: a systematic review. Journal of Neurosurgery: Pediatrics. 2013;12(2):134-41.
33. Zhang K, Hu W-h, Zhang C, Meng F-g, Chen N, Zhang J-g. Predictors of seizure freedom after surgical management of tuberous sclerosis complex: a systematic review and meta-analysis. Epilepsy research. 2013;105(3):377-83.
34. Josephson CB, Dykeman J, Fiest KM, Liu X, Sadler RM, Jette N, et al. Systematic review and meta-analysis of standard vs selective temporal lobe epilepsy surgery. Neurology. 2013;80(18):1669-76.
35. Fallah A, Guyatt GH, Snead OC, 3rd, Ebrahim S, Ibrahim GM, Mansouri A, et al. Predictors of seizure outcomes in children with tuberous sclerosis complex and intractable epilepsy undergoing resective epilepsy surgery: an individual participant data meta-analysis. PLoS One. 2013;8(2):e53565.
36. Ibrahim GM, Morgan BR, Fallah A. A partial least squares analysis of seizure outcomes following resective surgery for tuberous sclerosis complex in children with intractable epilepsy. Child's Nervous System. 2015;31(2):181-4.
37. Englot DJ, Breshears JD, Sun PP, Chang EF, Auguste KI. Seizure outcomes after resective surgery for extra–temporal lobe epilepsy in pediatric patients: A systematic review. Journal of Neurosurgery: Pediatrics. 2013;12(2):126-33.
38. Kuang Y, Yang T, Gu J, Kong B, Cheng L. Comparison of therapeutic effects between selective amygdalohippocampectomy and anterior temporal lobectomy for the treatment of temporal lobe epilepsy: a meta-analysis. British Journal of Neurosurgery. 2014;28(3):374-7.
39. Zhang J, Liu W, Chen H, Xia H, Zhou Z, Mei S, et al. Identification of common predictors of surgical outcomes for epilepsy surgery. Neuropsychiatric disease and treatment. 2013;9:1673.

40. Hu W-H, Zhang C, Zhang K, Meng F-G, Chen N, Zhang J-G. Selective amygdalohippocampectomy versus anterior temporal lobectomy in the management of mesial temporal lobe epilepsy: a meta-analysis of comparative studies: a systematic review. Journal of Neurosurgery. 2013;119(5):1089-97.
41. Bonney PA, Glenn CA, Ebeling PA, Conner AK, Boettcher LB, Cameron DM, et al. Seizure Freedom Rates and Prognostic Indicators After Resection of Gangliogliomas: A Review. World Neurosurg. 2015;84(6):1988-96.
42. Ruan D, Yu XB, Shrestha S, Wang L, Chen G. The Role of Hemosiderin Excision in Seizure Outcome in Cerebral Cavernous Malformation Surgery: A Systematic Review and Meta-Analysis. PLoS One. 2015;10(8):e0136619.
43. Cao K, Liu M, Wang C, Liu Q, Yang K, Tao L, et al. Five-year long-term prognosis of epileptic children after hemispheric surgery: A systematic review and meta-analysis. Medicine. 2016;95(23).
44. Wang X, Zhang C, Wang Y, Hu W, Shao X, Zhang J-g, et al. Prognostic factors for seizure outcome in patients with MRI-negative temporal lobe epilepsy: A meta-analysis and systematic review. Seizure. 2016;38:54-62.
45. Giridharan N, Horn PS, Greiner HM, Holland KD, Mangano FT, Arya R. Acute postoperative seizures as predictors of seizure outcomes after epilepsy surgery. Epilepsy Res. 2016;127:119-25.
46. Hu W-H, Zhang C, Zhang K, Shao X-Q, Zhang J-G. Hemispheric surgery for refractory epilepsy: a systematic review and meta-analysis with emphasis on seizure predictors and outcomes. Journal of neurosurgery. 2016;124(4):952-61.
47. Chen T, Guo L. The role of SISCOM in preoperative evaluation for patients with epilepsy surgery: a meta-analysis. Seizure. 2016;41:43-50.
48. Ampie L, Choy W, DiDomenico JD, Lamano JB, Williams CK, Kesavabhotla K, et al. Clinical attributes and surgical outcomes of angiocentric gliomas. J Clin Neurosci. 2016;28:117-22.
49. Harward SC, Chen WC, Rolston JD, Haglund MM, Englot DJ. Seizure outcomes in occipital lobe and posterior quadrant epilepsy surgery: a systematic review and meta-analysis. Neurosurgery. 2017;82(3):350-8.
50. Krucoff MO, Chan AY, Harward SC, Rahimpour S, Rolston JD, Muh C, et al. Rates and predictors of success and failure in repeat epilepsy surgery: A meta-analysis and systematic review. Epilepsia. 2017;58(12):2133-42.
51. Gloss D, Nolan SJ, Staba R. The role of high-frequency oscillations in epilepsy surgery planning. Cochrane Database of Systematic Reviews. 2014(1).
52. Jain P, Tomlinson G, Snead C, Sander B, Widjaja E. Systematic review and network meta-analysis of resective surgery for mesial temporal lobe epilepsy. Journal of Neurology, Neurosurgery & Psychiatry. 2018;89(11):1138-44.
53. Shan X, Fan X, Liu X, Zhao Z, Wang Y, Jiang T. Clinical characteristics associated with postoperative seizure control in adult low-grade gliomas: a systematic review and meta-analysis. Neuro-oncology. 2018;20(3):324-31.
54. Shang-Guan H-C, Wu Z-Y, Yao P-S, Chen G-R, Zheng S-F, Kang D-Z. Is Extended Lesionectomy Needed for Patients with Cerebral Cavernous Malformations Presenting with Epilepsy? A Meta-Analysis. World Neurosurgery. 2018;120:e984-e90.
55. Kobulashvili T, Kuchukhidze G, Brigo F, Zimmermann G, Höfler J, Leitinger M, et al. Diagnostic and prognostic value of noninvasive long-term video-electroencephalographic monitoring in epilepsy surgery: A systematic review and meta-analysis from the E-PILEPSY consortium. Epilepsia. 2018;59(12):2272-83.
56. Harris WB, Phillips HW, Chen JS, Weil AG, Ibrahim GM, Fallah A. Seizure outcomes in children with Rasmussen's encephalitis undergoing resective or hemispheric epilepsy surgery: an individual participant data meta-analysis. Journal of Neurosurgery: Pediatrics. 2019;1(aop):1-10.
57. Bjellvi J, Olsson I, Malmgren K, Ramsay KW. Epilepsy duration and seizure outcome in epilepsy surgery: A systematic review and meta-analysis. Neurology. 2019;93(2):e159-e66.

58. Chen J, Chen X, Huang C, Zhu H, Hou Z, An N, et al. Predictors of seizure recurrence in patients with surgery for focal cortical dysplasia: pairwise and network meta-analysis and trial sequential analysis. Child's Nervous System. 2019;35(5):753-67.
59. Toth M, Papp KS, Gede N, Farkas K, Kovacs S, Isnard J, et al. Surgical outcomes related to invasive EEG monitoring with subdural grids or depth electrodes in adults: A systematic review and meta-analysis. Seizure. 2019;70:12-9.
60. Widjaja E, Jain P, Demoe L, Guttmann A, Tomlinson G, Sander B. Seizure outcome of pediatric epilepsy surgery: Systematic review and meta-analyses. Neurology. 2020;94(7):311-21.
61. Brændholt M, Jensen M. Evidence From Meta-Analysis Supports Ictal Magnetoencephalographic Source Imaging as an Accurate Method in Presurgery Evaluation of Patients With Drug-Resistant Epilepsy. Clinical EEG and Neuroscience. 2020:1550059420921534.
62. Lamberink HJ, Otte WM, Blümcke I, Braun KP, Aichholzer M, Amorim I, et al. Seizure outcome and use of antiepileptic drugs after epilepsy surgery according to histopathological diagnosis: a retrospective multicentre cohort study. The Lancet Neurology. 2020;19(9):748-57.
63. Remick M, Ibrahim GM, Mansouri A, Abel TJ. Patient phenotypes and clinical outcomes in invasive monitoring for epilepsy: An individual patient data meta-analysis. Epilepsy Behav. 2020;102:106652.
64. Imberger G, Thorlund K, Gluud C, Wetterslev J. False-positive findings in Cochrane meta-analyses with and without application of trial sequential analysis: an empirical review. BMJ open. 2016;6(8):e011890.
65. Gleichgerrcht E, Keller SS, Drane DL, Munsell BC, Davis KA, Kaestner E, et al. Temporal lobe epilepsy surgical outcomes can be inferred based on structural connectome hubs: a machine learning study. Annals of Neurology. 2020;88(5):970-83.
66. Galovic M, Baudracco I, Wright-Goff E, Pillajo G, Nachev P, Wandschneider B, et al. Association of piriform cortex resection with surgical outcomes in patients with temporal lobe epilepsy. JAMA neurology. 2019;76(6):690-700.
