# Supplementary material for: Multimodal prognostic features of seizure freedom in epilepsy surgery
Source: J Neurol Neurosurg Psychiatry. 2022 Mar 4;93(5):499–508. doi: 10.1136/jnnp-2021-327119 (PMC9016256; doi:10.1136/jnnp-2021-327119)
Supplement: Supplementary data [file jnnp-2021-327119supp002.pdf]

```
dag {
bb="0,0,1,1"
"Age at onset" [exposure,pos="0.746,0.924"]
"Age at surgery" [exposure,pos="0.850,0.942"]
"CNS infections" [exposure,pos="0.524,0.574"]
"Clinical uncertainty" [latent,pos="0.419,0.576"]
"Complete excision" [exposure,pos="0.371,0.740"]
"Damaged anti-epileptogenic networks" [latent,pos="0.730,0.757"]
"Degree of multimodal concordance" [exposure,pos="0.432,0.484"]
"Duration of epilepsy" [exposure,pos="0.869,0.823"]
"Duration of follow-up" [outcome,pos="0.927,0.604"]
"E, O or ME" [adjusted,pos="0.317,0.213"]
"Engel Class" [outcome,pos="0.787,0.296"]
"Epilepsia partialis continua" [adjusted,pos="0.125,0.688"]
"Epileptic spasms" [exposure,pos="0.266,0.783"]
"Extensive or multilobar resection" [exposure,pos="0.590,0.854"]
"FCD and subtypes" [exposure,pos="0.301,0.121"]
"Febrile convulsions" [exposure,pos="0.695,0.117"]
"Focal > Multifocal > Generalised" [latent,pos="0.636,0.321"]
"Focal FDG-PET" [exposure,pos="0.225,0.484"]
"Focal MRI abnormality" [exposure,pos="0.441,0.201"]
"Focal ictal EEG" [exposure,pos="0.049,0.497"]
"Focal interictal EEG" [exposure,pos="0.078,0.590"]
"Focal pathology" [exposure,pos="0.333,0.380"]
"Focal seizure semiology" [exposure,pos="0.215,0.609"]
"Genetics: e.g. mTOR pathway" [exposure,pos="0.135,0.027"]
"Geographical location of surgery" [adjusted,pos="0.264,0.961"]
"H1 spectroscopic abnormality" [adjusted,pos="0.097,0.328"]
"Hippocampal Sclerosis" [exposure,pos="0.386,0.040"]
"History of head injury" [exposure,pos="0.546,0.134"]
"ILAE scale" [outcome,pos="0.764,0.359"]
```

"Intraoperative EEG" [adjusted,pos="0.452,0.650"]

"Invasive EEG" [exposure,pos="0.258,0.881"]

"Lateralised EEG" [exposure,pos="0.069,0.399"]

"New or change in seizure semiology" [exposure,pos="0.573,0.943"]

"New seizure-focus" [latent,pos="0.893,0.705"]

"Number of cortical tubers" [adjusted,pos="0.508,0.270"]

"Postoperative discharges" [outcome,pos="0.926,0.374"]

"Postsurgical Seizure Freedom" [outcome,pos="0.879,0.518"]

"Presence of limited EZ" [latent,pos="0.740,0.452"]

"Proportion of EZ resected" [latent,pos="0.749,0.659"]

"SISCOM abnormalities" [exposure,pos="0.478,0.341"]

"Seizure frequency" [exposure,pos="0.602,0.510"]

"Severe learning disability" [exposure,pos="0.665,0.220"]

"Side of resection" [adjusted,pos="0.410,0.961"]

"Surgical technique" [exposure,pos="0.387,0.834"]

"TL vs ET resections" [exposure,pos="0.546,0.772"]

"Theoretical \"focal lesion\"" [latent,pos="0.222,0.270"]

"Vascular lesions" [adjusted,pos="0.415,0.132"]

"Video-EEG telemetry" [adjusted,pos="0.076,0.781"]

"Without APOS" [outcome,pos="0.893,0.272"]

"sEEG vs subdural grid" [exposure,pos="0.123,0.866"]

Astrocytoma [pos="0.078,0.280"]

Encephalomalacia [pos="0.555,0.064"]

Neuromigrational [adjusted,pos="0.068,0.137"]

Sex [adjusted,pos="0.246,0.063"]

Tumours [exposure,pos="0.126,0.188"]

"Age at onset" -> "Duration of epilepsy"

"Age at surgery" -> "Duration of epilepsy"

"CNS infections" -> "Focal > Multifocal > Generalised" [pos="0.576,0.374"]

"CNS infections" -> "Focal pathology" [pos="0.576,0.385"]

"Clinical uncertainty" -> "Intraoperative EEG"

"Clinical uncertainty" -> "Invasive EEG" [pos="0.169,0.713"]

"Complete excision" -> "Proportion of EZ resected"

"Damaged anti-epileptogenic networks" -> "New seizure-focus"

"Damaged anti-epileptogenic networks" -> "Postsurgical Seizure Freedom"

"Damaged anti-epileptogenic networks" -> "Seizure frequency"

"Damaged anti-epileptogenic networks" <-> "Focal > Multifocal > Generalised" [pos="0.665,0.562"]

"Degree of multimodal concordance" -> "Clinical uncertainty"

"Degree of multimodal concordance" -> "Complete excision" [pos="0.344,0.598"]

"Degree of multimodal concordance" -> "Focal > Multifocal > Generalised"

"Degree of multimodal concordance" -> "Presence of limited EZ"

"Degree of multimodal concordance" -> "Proportion of EZ resected"

"Duration of epilepsy" -> "Damaged anti-epileptogenic networks"

"Duration of epilepsy" -> "New seizure-focus"

"Duration of epilepsy" -> "Severe learning disability" [pos="0.780,0.591"]

"Duration of epilepsy" <-> "TL vs ET resections" [pos="0.665,0.787"]

"Duration of follow-up" <-> "Postsurgical Seizure Freedom" [pos="0.921,0.556"]

"E, O or ME" -> "Focal MRI abnormality"

"Epilepsia partialis continua" <-> "Focal seizure semiology" [pos="0.124,0.653"]

"Epileptic spasms" -> "Focal seizure semiology" [pos="0.257,0.705"]

"Extensive or multilobar resection" -> "Damaged anti-epileptogenic networks"

"Extensive or multilobar resection" <-> "TL vs ET resections" [pos="0.609,0.797"]

"FCD and subtypes" -> "Focal pathology"

"FCD and subtypes" -> "Theoretical \"focal lesion\"" [pos="0.205,0.169"]

"Febrile convulsions" -> "Focal > Multifocal > Generalised" [pos="0.573,0.171"]

"Febrile convulsions" <-> "Focal MRI abnormality"

"Febrile convulsions" <-> "TL vs ET resections" [pos="0.774,0.366"]

"Focal > Multifocal > Generalised" -> "Clinical uncertainty" [pos="0.583,0.498"]

"Focal > Multifocal > Generalised" -> "Presence of limited EZ"

"Focal > Multifocal > Generalised" -> "Seizure frequency"

"Focal > Multifocal > Generalised" <-> "New seizure-focus" [pos="0.638,0.566"]

"Focal > Multifocal > Generalised" <-> "Severe learning disability" [pos="0.717,0.288"]

"Focal FDG-PET" -> "Degree of multimodal concordance"

"Focal MRI abnormality" -> "Degree of multimodal concordance" [pos="0.390,0.379"]

"Focal MRI abnormality" -> "Focal FDG-PET" [pos="0.269,0.325"]

"Focal MRI abnormality" -> "Focal ictal EEG"

"Focal MRI abnormality" -> "Focal interictal EEG" [pos="0.173,0.414"]

"Focal MRI abnormality" -> "Focal seizure semiology" [pos="0.412,0.402"]

"Focal MRI abnormality" -> "Number of cortical tubers" [pos="0.519,0.203"]

"Focal MRI abnormality" -> "Vascular lesions"

"Focal ictal EEG" -> "Degree of multimodal concordance" [pos="0.194,0.422"]

"Focal ictal EEG" -> "Focal seizure semiology"

"Focal ictal EEG" -> "Video-EEG telemetry" [pos="0.001,0.606"]

"Focal ictal EEG" <-> "Focal interictal EEG"

"Focal ictal EEG" <-> "Lateralised EEG"

"Focal interictal EEG" -> "Degree of multimodal concordance"

"Focal interictal EEG" -> "Video-EEG telemetry" [pos="0.036,0.641"]

"Focal interictal EEG" <-> "Lateralised EEG" [pos="0.110,0.470"]

"Focal pathology" -> "Complete excision" [pos="0.300,0.619"]

"Focal pathology" -> "Degree of multimodal concordance" [pos="0.275,0.434"]

"Focal pathology" -> "Focal FDG-PET"

"Focal pathology" -> "Focal MRI abnormality"

"Focal pathology" -> "Focal ictal EEG"

"Focal pathology" -> "Focal interictal EEG"

"Focal pathology" -> "Focal seizure semiology"

"Focal pathology" -> "H1 spectroscopic abnormality"

"Focal pathology" -> "Lateralised EEG"

"Focal pathology" -> "Vascular lesions" [pos="0.353,0.238"]

"Focal pathology" <-> "Theoretical \"focal lesion\""

"Focal seizure semiology" -> "Degree of multimodal concordance" [pos="0.334,0.548"]

"Focal seizure semiology" -> "Video-EEG telemetry" [pos="0.224,0.729"]

"Genetics: e.g. mTOR pathway" -> "Degree of multimodal concordance" [pos="0.267,0.465"]

"Genetics: e.g. mTOR pathway" -> "FCD and subtypes"

"Genetics: e.g. mTOR pathway" -> "Febrile convulsions" [pos="0.660,0.002"]

"Genetics: e.g. mTOR pathway" -> "Focal ictal EEG" [pos="0.001,0.162"]

"Genetics: e.g. mTOR pathway" -> "Focal interictal EEG" [pos="0.124,0.480"]

"Genetics: e.g. mTOR pathway" -> "Focal pathology" [pos="0.309,0.255"]

"Genetics: e.g. mTOR pathway" -> "Hippocampal Sclerosis"

"Genetics: e.g. mTOR pathway" -> "Lateralised EEG" [pos="0.014,0.199"]

"Genetics: e.g. mTOR pathway" -> "Theoretical \"focal lesion\""

"Genetics: e.g. mTOR pathway" -> Astrocytoma [pos="0.181,0.263"]

"Genetics: e.g. mTOR pathway" -> Neuromigrational

"Genetics: e.g. mTOR pathway" -> Sex

"Genetics: e.g. mTOR pathway" -> Tumours

"Geographical location of surgery" <-> "Surgical technique"

"Hippocampal Sclerosis" -> "Febrile convulsions"

"Hippocampal Sclerosis" -> "Focal MRI abnormality" [pos="0.488,0.082"]

"Hippocampal Sclerosis" -> "Theoretical \"focal lesion\"" [pos="0.170,0.090"]

"History of head injury" -> "Focal > Multifocal > Generalised"

"History of head injury" -> "Focal MRI abnormality"

"History of head injury" -> "Theoretical \"focal lesion\"" [pos="0.234,0.154"]

"History of head injury" -> Encephalomalacia

"Intraoperative EEG" <-> "Surgical technique" [pos="0.477,0.695"]

"Invasive EEG" -> "Degree of multimodal concordance" [pos="0.169,0.770"]

"Invasive EEG" -> "sEEG vs subdural grid"

"Lateralised EEG" -> "Degree of multimodal concordance" [pos="0.206,0.402"]

"Lateralised EEG" -> "Focal seizure semiology"

"New seizure-focus" -> "New or change in seizure semiology" [pos="0.855,0.767"]

"New seizure-focus" -> "Postsurgical Seizure Freedom" [pos="0.850,0.627"]

"Postoperative discharges" -> "Without APOS" [pos="0.867,0.342"]

"Postsurgical Seizure Freedom" -> "Engel Class"

"Postsurgical Seizure Freedom" -> "ILAE scale"

"Postsurgical Seizure Freedom" -> "Postoperative discharges"

"Postsurgical Seizure Freedom" -> "Without APOS" [pos="0.832,0.352"]

BMJ Publishing Group Limited (BMJ) disclaims all liability and responsibility arising from any reliance placed on this supplemental material which has been supplied by the author(s)


```
"Presence of limited EZ" -> "Postsurgical Seizure Freedom" [pos="0.790,0.480"]
"Presence of limited EZ" -> "Proportion of EZ resected"
"Proportion of EZ resected" -> "Damaged anti-epileptogenic networks"
"Proportion of EZ resected" -> "New seizure-focus"
"Proportion of EZ resected" -> "Postsurgical Seizure Freedom"
"SISCOM abnormalities" -> "Degree of multimodal concordance"
"Side of resection" -> "Surgical technique"
"Side of resection" -> "TL vs ET resections" [pos="0.479,0.850"]
"Surgical technique" -> "Damaged anti-epileptogenic networks" [pos="0.518,0.684"]
"Surgical technique" -> "Extensive or multilobar resection"
"Surgical technique" -> "Proportion of EZ resected" [pos="0.484,0.652"]
"Surgical technique" <-> "TL vs ET resections"
"TL vs ET resections" -> "Damaged anti-epileptogenic networks"
"TL vs ET resections" -> "Proportion of EZ resected"
"Theoretical \"focal lesion\"" -> "Complete excision" [pos="0.299,0.516"]
"Theoretical \"focal lesion\"" -> "Focal > Multifocal > Generalised"
"Theoretical \"focal lesion\"" -> "Focal FDG-PET"
"Theoretical \"focal lesion\"" -> "Focal MRI abnormality"
"Theoretical \"focal lesion\"" -> "Focal ictal EEG" [pos="0.231,0.372"]
"Theoretical \"focal lesion\"" -> "Focal interictal EEG" [pos="0.191,0.324"]
"Theoretical \"focal lesion\"" -> "Focal seizure semiology"
"Theoretical \"focal lesion\"" -> "H1 spectroscopic abnormality"
"Theoretical \"focal lesion\"" -> "SISCOM abnormalities"
"sEEG vs subdural grid" -> "Degree of multimodal concordance" [pos="0.345,0.553"]
Astrocytoma -> Tumours
Encephalomalacia -> "Focal MRI abnormality" [pos="0.486,0.095"]
Encephalomalacia -> "Focal pathology" [pos="0.496,0.074"]
Neuromigrational -> "Focal MRI abnormality" [pos="0.292,0.154"]
Neuromigrational -> "Focal pathology" [pos="0.095,0.297"]
Neuromigrational -> "Severe learning disability" [pos="0.559,0.164"]
Neuromigrational -> "Theoretical \"focal lesion\"" [pos="0.219,0.168"]
```

```
Tumours -> "E, O or ME" [pos="0.193,0.207"]

Tumours -> "Focal pathology" [pos="0.119,0.283"]

Tumours -> "Theoretical \"focal lesion\""

}
```
