# Supplementary material for: Multimodal prognostic features of seizure freedom in epilepsy surgery
Source: J Neurol Neurosurg Psychiatry. 2022 Mar 4;93(5):499–508. doi: 10.1136/jnnp-2021-327119 (PMC9016256; doi:10.1136/jnnp-2021-327119)
Supplement: Supplementary data [file jnnp-2021-327119supp003.pdf]

```
dag {
bb="0,0,1,1"
"Age at onset" [exposure,pos="0.828,0.888"]
"Age at surgery" [exposure,pos="0.904,0.888"]
"CNS infections" [exposure,pos="0.633,0.208"]
"Clinical uncertainty" [latent,pos="0.376,0.466"]
"Complete excision" [exposure,pos="0.383,0.384"]
"Damaged anti-epileptogenic networks" [latent,pos="0.730,0.757"]
"Degree of multimodal concordance" [exposure,pos="0.425,0.568"]
"Duration of epilepsy" [exposure,pos="0.869,0.823"]
"Duration of follow-up" [outcome,pos="0.934,0.407"]
"Engel Class" [outcome,pos="0.787,0.296"]
"Epileptic spasms" [exposure,pos="0.038,0.660"]
"Extensive or multilobar resection" [exposure,pos="0.551,0.888"]
"FCD and subtypes" [exposure,pos="0.127,0.095"]
"Febrile convulsions" [exposure,pos="0.630,0.034"]
"Focal > Multifocal > Generalised" [latent,pos="0.597,0.449"]
"Focal EEG" [pos="0.124,0.433"]
"Focal FDG-PET" [exposure,pos="0.352,0.238"]
"Focal Imaging Abnormality" [pos="0.459,0.247"]
"Focal MRI abnormality" [exposure,pos="0.348,0.177"]
"Focal histopathology" [pos="0.126,0.166"]
"Focal ictal EEG" [exposure,pos="0.126,0.368"]
"Focal interictal EEG" [exposure,pos="0.219,0.369"]
"Focal seizure semiology" [exposure,pos="0.142,0.661"]
"Genetics: e.g. mTOR pathway" [exposure,pos="0.135,0.027"]
"Hippocampal Sclerosis" [exposure,pos="0.230,0.095"]
"History of head injury" [exposure,pos="0.630,0.092"]
"ILAE scale" [outcome,pos="0.764,0.359"]
"Invasive EEG" [exposure,pos="0.205,0.483"]
"Lateralised EEG" [exposure,pos="0.037,0.367"]
```

"New or change in seizure semiology" [exposure,pos="0.717,0.888"]

"New seizure-focus" [latent,pos="0.893,0.705"]

"Postoperative discharges" [outcome,pos="0.905,0.345"]

"Postsurgical Seizure Freedom" [outcome,pos="0.861,0.466"]

"Presence of limited EZ" [latent,pos="0.740,0.452"]

"Proportion of EZ resected" [latent,pos="0.734,0.603"]

"SISCOM abnormalities" [exposure,pos="0.351,0.301"]

"Seizure frequency" [exposure,pos="0.439,0.685"]

"Severe learning disability" [exposure,pos="0.631,0.152"]

"Surgical technique" [exposure,pos="0.524,0.823"]

"TL vs ET resections" [exposure,pos="0.436,0.888"]

"Without APOS" [outcome,pos="0.843,0.246"]

"sEEG vs subdural grid" [exposure,pos="0.219,0.425"]

Tumours [exposure,pos="0.046,0.095"]

"Age at onset" -> "Duration of epilepsy"

"Age at surgery" -> "Duration of epilepsy"

"CNS infections" -> "Focal > Multifocal > Generalised" [pos="0.554,0.201"]

"Clinical uncertainty" -> "Extensive or multilobar resection" [pos="0.292,0.686"]

"Clinical uncertainty" -> "Invasive EEG"

"Complete excision" -> "Focal > Multifocal > Generalised"

"Damaged anti-epileptogenic networks" -> "New seizure-focus"

"Degree of multimodal concordance" -> "Clinical uncertainty"

"Degree of multimodal concordance" -> "Focal > Multifocal > Generalised"

"Degree of multimodal concordance" -> "Presence of limited EZ"

"Degree of multimodal concordance" -> "Proportion of EZ resected"

"Degree of multimodal concordance" -> "Seizure frequency"

"Duration of epilepsy" -> "New seizure-focus"

"Duration of follow-up" <-> "Postsurgical Seizure Freedom" [pos="0.932,0.570"]

"Engel Class" <-> "Postsurgical Seizure Freedom"

"Epileptic spasms" <-> "Focal seizure semiology"

"Extensive or multilobar resection" <-> "Surgical technique"

BMJ Publishing Group Limited (BMJ) disclaims all liability and responsibility arising from any reliance placed on this supplemental material which has been supplied by the author(s)

"Extensive or multilobar resection" <-> "TL vs ET resections"

"FCD and subtypes" -> "Focal histopathology"

"Febrile convulsions" -> "Focal > Multifocal > Generalised" [pos="0.453,0.102"]

"Febrile convulsions" <-> "Hippocampal Sclerosis"

"Focal > Multifocal > Generalised" -> "Clinical uncertainty"

"Focal > Multifocal > Generalised" -> "Presence of limited EZ"

"Focal > Multifocal > Generalised" -> "Seizure frequency"

"Focal > Multifocal > Generalised" <-> "TL vs ET resections"

"Focal EEG" -> "Degree of multimodal concordance" [pos="0.164,0.599"]

"Focal EEG" -> "Epileptic spasms"

"Focal EEG" -> "Focal seizure semiology"

"Focal FDG-PET" -> "Focal Imaging Abnormality"

"Focal FDG-PET" <-> "Focal MRI abnormality"

"Focal FDG-PET" <-> "SISCOM abnormalities"

"Focal Imaging Abnormality" -> "Degree of multimodal concordance"

"Focal MRI abnormality" -> "Focal Imaging Abnormality"

"Focal histopathology" -> "Complete excision"

"Focal histopathology" -> "Focal MRI abnormality"

"Focal histopathology" -> "Focal ictal EEG"

"Focal histopathology" -> "Focal interictal EEG"

"Focal histopathology" -> "Lateralised EEG"

"Focal ictal EEG" -> "Focal EEG"

"Focal interictal EEG" -> "Focal EEG"

"Focal seizure semiology" -> "Degree of multimodal concordance"

"Genetics: e.g. mTOR pathway" -> "FCD and subtypes"

"Genetics: e.g. mTOR pathway" -> "Febrile convulsions"

"Genetics: e.g. mTOR pathway" -> "Focal EEG" [pos="0.007,0.232"]

"Genetics: e.g. mTOR pathway" -> "Hippocampal Sclerosis"

"Genetics: e.g. mTOR pathway" -> Tumours

"Hippocampal Sclerosis" -> "Focal histopathology"

"History of head injury" -> "Focal > Multifocal > Generalised" [pos="0.480,0.109"]

BMJ Publishing Group Limited (BMJ) disclaims all liability and responsibility arising from any reliance placed on this supplemental material which has been supplied by the author(s)


```
"ILAE scale" <-> "Postsurgical Seizure Freedom"
"Invasive EEG" -> "Focal EEG"
"Invasive EEG" -> "sEEG vs subdural grid"
"Lateralised EEG" -> "Focal EEG"
"New seizure-focus" -> "New or change in seizure semiology"
"New seizure-focus" -> "Postsurgical Seizure Freedom"
"New seizure-focus" -> "Presence of limited EZ"
"Postoperative discharges" <-> "Postsurgical Seizure Freedom"
"Postoperative discharges" <-> "Without APOS" [pos="0.866,0.292"]
"Postsurgical Seizure Freedom" <-> "Without APOS" [pos="0.850,0.338"]
"Presence of limited EZ" -> "Postsurgical Seizure Freedom"
"Presence of limited EZ" -> "Proportion of EZ resected"
"Proportion of EZ resected" -> "Postsurgical Seizure Freedom"
"SISCOM abnormalities" -> "Focal Imaging Abnormality"
"Severe learning disability" -> "Focal > Multifocal > Generalised" [pos="0.519,0.142"]
"Surgical technique" -> "Damaged anti-epileptogenic networks"
"Surgical technique" -> "Proportion of EZ resected"
"Surgical technique" <-> "TL vs ET resections"
"sEEG vs subdural grid" -> "Focal EEG"
Tumours -> "Focal histopathology"
}
```
